# Effect of telemedicine on the quality of life of people with heart disease: a systematic review

**DOI:** 10.1590/1518-8345.7243.4566

**Published:** 2025-07-11

**Authors:** Gabriele Cardoso Gonçalves Alves, Fabiola Leticia Damascena Amador, Vagner Rogério dos Santos, Rita Simone Lopes Moreira

**Affiliations:** 1 Universidade Federal de São Paulo, Escola Paulista de Enfermagem, São Paulo, SP, Brazil.; 2 Universidade Federal de São Paulo, Escola Paulista de Medicina, São Paulo, SP, Brazil.

**Keywords:** Telemedicine, Heart Diseases, Self-Management, Quality of Life, Telerehabilitation, Self Care

## Abstract

to synthesize the scientific evidence on the effectiveness of telemedicine-based interventions in improving health-related quality of life and self-management of patients with heart disease.

systematic review of effectiveness, following the recommendations of the Joanna Briggs Institute and the reporting guideline Preferred Reporting Items for Systematic Reviews and Meta-Analyses Checklist. The search was conducted in six databases: Cochrane Library, Virtual Health Library, PubMed, CINAHL, Web of Science Core Collection and Scopus, without period restriction, in English, Portuguese or Spanish. The methodological quality and risk of bias of the studies were assessed using the JBI critical appraisal tool and the certainty of the evidence was classified using the GRADE tool.

of the 44 randomized clinical trials included, the main interventions analyzed were telemonitoring, telephone contact and telerehabilitation. Out of the studies evaluated, 88.63% demonstrated an improvement in health-related quality of life, with 45.45% of these showing a statistically significant difference.

telemedicine shows promise as a valuable tool for the care and self-management of individuals with cardiac conditions. However, further studies are needed to confirm its effectiveness and impact on health outcomes.

## Highlights

**(1)** The main resources observed were: telemonitoring, tele-connection and telerehabilitation. **(2)** Telemedicine can be an effective tool in the care of cardiac conditions. **(3)** The real impact on quality of life in cardiac diseases is still uncertain. **(4)** Studies related to aspects of quality of life are still needed.

## Introduction

Chronic non-communicable diseases (NCDs) represent a significant portion of the causes of death worldwide, accounting for approximately 74% of deaths globally^(1)^. Among them, cardiovascular diseases (CVDs) are an alarming factor, having been responsible for approximately two million deaths in the Americas in 2019^(2)^.

In this scenario, addressing the challenge of CVDs has become a priority, especially due to the continuous aging of the world’s population and the multifactorial complexity that characterizes these diseases^(3)^. In addition, NCDs, especially heart disease, are becoming a growing marker of global inequalities, being highly prevalent in developing countries^(4)^.

Heart diseases are pathological conditions involving the heart, in terms of its structure and function^(5)^. The development, progression and worsening of these conditions are directly related to risk factors, which can be classified as potentially modifiable, such as controlling blood pressure, blood glucose and lipid profile, or modifiable, such as smoking cessation, reducing excessive alcohol consumption, combating obesity, sedentary lifestyle, among others^(6)^. Thus, the use of educational initiatives that seek to promote health literacy is recommended, since these actions have the potential to act on such risk factors, mitigating adverse events and hospital readmissions, while at the same time increasing the health-related quality of life (HRQoL) of affected individuals^(7)^. In this context, it is observed that populations at high risk of CVD have more unfavorable HRQoL results^(7)^. Thus, this variable emerges as a crucial indicator, being a strong predictor of both mortality and hospitalization for heart failure (HF), regardless of the severity of symptoms or ejection fraction^(8)^. Thus, in order to address this reality, telemedicine emerges as a promising strategy to reduce mortality in individuals with heart problems and promote HRQoL and self-care^(9)^. Furthermore, as shown in a systematic review on the effectiveness, acceptability and costs of telemedicine carried out in 2015, when considering strategies to improve risk factors, there was an improvement in HRQoL, a decrease in levels of glycated hemoglobin, low-density lipoproteins and blood pressure in individuals with NCDs^(10)^.

It is undeniable that during the coronavirus pandemic there was widespread adoption of this remote care format as an alternative to in-person consultations, in response to restrictions on direct contact, demonstrating the system’s ability to adapt to emerging challenges^(11)^.

Telemedicine is defined as the provision of health services remotely^(12)^, i.e., the patient and the provider are separated by distance, mediated by a technological tool^(13)^. There are several resources in this type of health care, such as consultations with health professionals (teleconsultation, hotlines and support lines), telemonitoring, telerehabilitation, storage and forwarding of health data (such as images, notes and videos) to care providers and teleconsultation between health professionals who provide care, communicating in search of other opinions for case management^(13-14)^.

In view of this, telemedicine plays an essential role in global health care, being able to structure therapeutic initiatives that encompass educational objectives, accurate diagnoses and continuous monitoring^(15-16)^. Furthermore, by overcoming geographic limitations^(15)^, it has the potential to increase access to care, reduce costs and improve overall health outcomes^(16)^. Thus, there is concern among health professionals regarding the ability of individuals to self-manage their own conditions in this care format^(9)^.

Self-management is the “ability to manage symptoms, treatment, physical and psychosocial changes, and lifestyle changes that patients develop when dealing with chronic diseases”^(17)^, usually requiring the support of a health professional^(18)^. In this context, it is believed that telemedicine has a positive impact on supporting self-management, consequently improving the HRQoL of patients with heart disease.

To date, previous systematic reviews that addressed heart disease and telemedicine have not focused specifically on self-management and changes in HRQoL over time, addressing other outcomes such as mortality, hospitalization, and others^(19-23)^. Furthermore, studies related to HRQoL perceived by patients have shown divergent results^(19-20,23-24)^ and, as shown in a systematic review, studies using standardized measures to assess HRQoL, self-care and satisfaction are needed^(10)^.

In view of this, this review aimed to synthesize the scientific evidence on the effectiveness of telemedicine-based interventions in improving HRQoL and self-management of patients with heart disease.

## Method

This is a systematic review, conducted according to the recommendations of the Joanna Briggs Institute (JBI): Evidence Synthesis Groups^(25)^ and reported according to the recommendations of the Preferred Reporting Items for Systematic Reviews and Meta-Analyses PRISMA Checklist^(26)^. The protocol was previously published in the Open Science Framework^(27)^ on January 22^nd^, 2021, under DOI number 10.17605/OSF.IO/HQWGT. The search was conducted in March 2023.

The research was guided by the acronym PICOS (P – Population; I – Intervention; C – Comparison; O - Outcomes; S -Study)^(25)^, with P (individuals aged 18 years or older with heart disease), I (telemedicine resources that support self-management), C (usual care, based on outpatient consultations), O (HRQoL) and S (Randomized controlled clinical trials). Based on this acronym, the following guiding question was developed: How effective are telemedicine interventions based on self-management compared to usual care on the HRQoL of adult patients with heart disease?

## Eligibility criteria

Randomized controlled trials (RCTs) that compared usual care with the use of telemedicine in adult patients (≥ 18 years) with heart disease, evaluating the outcomes in HRQoL and self-management of these individuals, were included. The exclusion criteria were: RCTs that did not detail the methodology used and articles that included other clinical conditions (e.g., cancer and diabetes).

## Data sources

To select the articles, the search strategy was implemented by the main researcher in the following databases: Cochrane Library (Wiley), Virtual Health Library (*Portal BVS*), Medical Literature Analysis and Retrieval System Online (MEDLINE, PubMed), CINAHL (EBSCOhost), Web of Science Core Collection and Scopus (via *Portal Periódicos CAPES* website).

## Search strategy

The search strategy was specific to each database using two controlled vocabularies in health: Medical Subject Headings (MeSH) and Health Sciences Descriptors (DeCS). The descriptors used were: “Telemedicine”, “Heart Diseases”, “Self-Management”, “Cardiac Rehabilitation” and “Health Education”, appropriate for each database. The complete search strategies can be found in the Supplementary Material (https://doi.org/10.48331/scielodata.MI2JBD).

## Data selection and extraction

Two independent reviewers selected the studies, and a third reviewer resolved disagreements. First, titles and abstracts related to the research question and study objective were identified, and those potentially eligible were pre-selected. In the second stage, two independent reviewers evaluated the full texts of the pre-selected studies to confirm their eligibility. The selection process was performed using the Rayyan QCRI platform^(28)^.

Data were extracted from the studies included in the review by two independent reviewers using a predetermined data extraction form. The form included the following axes: methodological details, intervention, and results [the form can be found in the Supplementary Material (https://doi.org/10.48331/scielodata.MI2JBD)]. Due to the complexity of the interventions, the selected studies were categorized according to the population studied and the intervention performed, considering the resources used. The extracted data included specific details about the participants, study methods, interventions, and results significant for the purpose of the review. Any disagreements that arose between reviewers were resolved through discussion or with a third reviewer. It was not necessary to contact the authors of the articles for additional information.

## Data synthesis

The findings were presented in narrative form. The narrative synthesis included a detailed description of the included studies, categorized according to the study population, questionnaires used to measure HRQoL, follow-up time and the telemedicine interventions performed. Tables and figures were used to assist in the presentation of the data.

## Critical evaluation of studies

Two independent reviewers performed a critical appraisal of all studies that met the inclusion criteria. Any discrepancies between the reviewers were resolved by consensus or by the intervention of a third reviewer. The methodological quality of the studies was assessed using the JBI critical appraisal checklist for randomized controlled trials. This checklist consists of 13 questions whose answers can be “yes”, “no”, “unclear” or “not applicable”^(29)^. All studies were classified into five different domains, resulting in classifications of low risk of bias, moderate risk of bias or high risk of bias. The certainty of the evidence for the HRQoL outcome was assessed using the Grading of Recommendations Assessment, Development and Evaluation (GRADE) instrument^(30)^.

## Results

The initial literature search identified a total of 2,341 studies from databases, in addition to four additional studies found through manual searches of reference lists of included articles. Of these, two studies were included in the primary search to develop the search strategy, while the other two were identified after analysis of the study protocols found in the final search.

After removing duplicates and reviewing the titles and abstracts, 80 studies were selected for full reading. The reasons for exclusion at this stage were mainly related to criteria such as population, intervention, comparison or outcome. Details on the reasons for exclusion can be found in the Supplementary Material (https://doi.org/10.48331/scielodata.MI2JBD).

**Figure 1 -:**
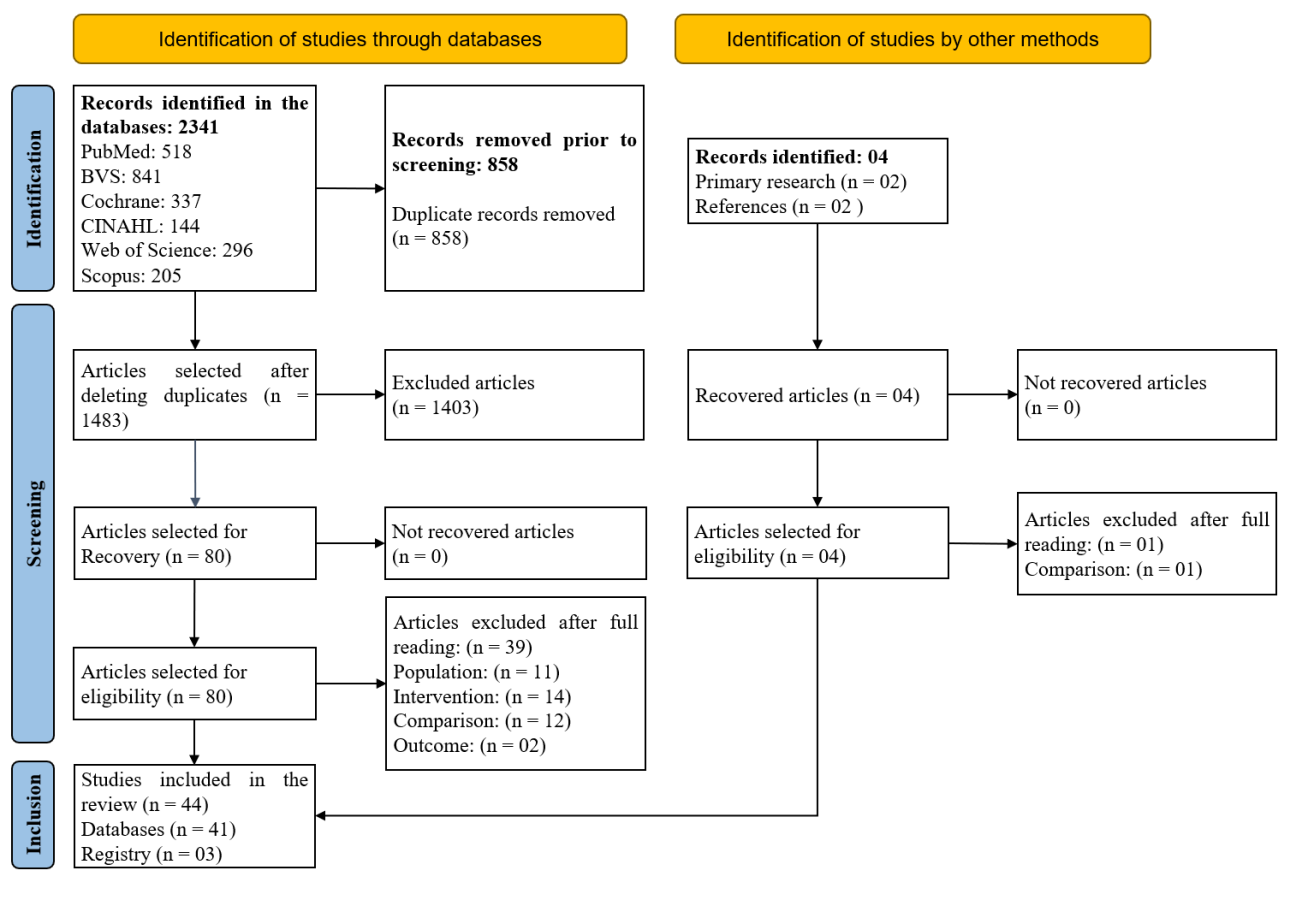
PRISMA flowchart^(26)^

Based on the full reading and analysis of the methodological quality of the studies, considering the eligibility criteria, the final sample was constituted, which included 44 randomized clinical trials. Figure 1 presents the detailed flowchart of the selection process of the studies included in this systematic review.

The descriptive summary of the RCTs included in this review is presented in following Figure 2.

The 44 RCTs included in this review involved a total of 12,732 patients, of whom 6,233 were allocated to the control group and 6,499 to the intervention group. The research was conducted on four continents, with 40.91% (n = 18) of the studies conducted in Europe^(32,34-35,38-42,44,48,52,54,56-57,63-64,67,71)^, 36.36% (n = 16) in North America^(37,43-46,51-53,59-60,62,65-66-67,63-64,66,68-70,72-74)^, 13.64% (n = 6) in Oceania^(33,36,50,55,58,61)^ and 9.09% (n = 4) in Asia^(31,45,47,49)^. The follow-up period ranged from one to 26 months, with an average follow-up of 8.4 months.

**Figure 2 -:** Description of the characteristics of the studies included in the systematic review (n = 44)

**Author, year, country**	**Population, n, follow-up**	**Intervention**	**Resources**	**Comparation**	**Main outcomes**	**Main results**
Choi, et al. ^( 31 )^ 2023 Korea	Individuals diagnosed with HF* n:76 (CG ^†^ :38| IG ^‡^ : 38) Follow-up: 3 months	Self-management program via mobile application (“Heart Failure-Smart Life”)	“Heart Failure-Smart Life” app: Educational content One-on-one chat with healthcare professional Daily health records for personalized and interactive monitoring, with features tailored to individual needs Self-management feedback from nurse Distinct app for healthcare professionals	Usual care: consultations with a cardiologist and cardiology nurse; brief information about medications and the progression of the disease	Anthropometric measurements; NYHA functional classification ^§^ Depression (Geriatric Depression scale) HRQoL ^||^ (MacNew Heart Disease Health-Related QoL) Medication adherence (Hill-Bone Medication Adherence) Self-care (European Heart Failure Self-Care Behaviour Scale)	In the comparison between groups, there were significant improvements in the NYHA ^§^ functional class (p = 0.003) in IG ^‡^ . HRQoL ^||^ showed no significant differences between the groups (CG ^†^ =5.34; IG ^‡^ =5.62). Self-care behavior improved over time (p < 0.001), but without significant differences between the groups.
Dalli Peydró, et al. ^( 32 )^ 2022 Spain	Individuals after hospital discharge due to ACS ^¶^ n: 59 (CG ^†^ : 28 | IG ^‡^ : 31) Follow-up: 10 months	Telerehabilitation, preceded by in-hospital training for 2 weeks, after adjustments to pace and goals, the app guided participants through a daily exercise program	App: Daily physical activity program General health record Vital signs Medication adherence HR** monitor	Center-based cardiac rehabilitation	Increased reported physical activity (International Physical Activity Questionnaire) VO _2_^††^ max HRQoL ^||^ (EQ-5D ^‡‡^ 5-levels)	The IG ^‡^ showed a greater increase in physical activity (1,726 vs. 636 multiples of metabolic equivalents -min/week, p = 0.045) and VO _2_^††^ max (1.62 vs. 0.60 ml/kg min), p < 0.004). Adherence to the Mediterranean diet, psychological distress and HRQoL ^||^ showed greater improvement in the IG ^‡^ than in the CG ^†^ . Self-rated health improved in both groups, but was only significant in the IG ^‡^ (p = 0.008)
Chow, et al. ^( 33 )^ 2022 Australia	Individuals diagnosed with SCA¶ n:1424 (CG ^†^ :708| IG ^‡^ :716) Follow-up: 12 months	Personalized SMS ^§§^ program aimed at improving medication adherence and secondary prevention	Sending motivational and educational SMS ^§§^ divided into three main modules: lifestyle (diet, exercise, smoking cessation), medications (information on use and side effects), general secondary prevention (health goals, mental health support and health services) Health advisor	Usual care was not described	Self-reported medication adherence Anthropometric and laboratory measurements HRQoL ^||^ (SF ^||||^ -12)	There was no significant difference in self-reported medication adherence between IG ^‡^ and CG ^†^ 0.93 [95% confidence interval, 0.84–1.03]; P=0.15). There was no difference in depression and anxiety scores, but there was a slight improvement in HRQoL ^§^ (physical component) score for IG ^‡^ (mean difference [95% confidence interval], 1.1 [0.0, 2.2]; P=0.045).
Voller, et al. ^( 34 )^ 2022 Germany	Individuals diagnosed with HF* n:621 (CG ^†^ :319 | IG ^‡^ :302) Follow-up: 12 months	Home telemonitoring via the Motiva ^®^ system, which enables patients to manage their health condition and facilitates daily contact between doctors and patients	Motiva ^®^ System Regular recording of vital parameters (BP ^¶¶^ , HR** and weight) Educational material, training material, questionnaires, reminders and feedback on health status via Motiva ^®^ Telephone monitoring of possible decompensations	Usual care, based on the guidelines of the European Society of Cardiology	Incremental cost-effectiveness Mortality HRQoL ^||^ (SF ^||||^ -36 V2; WHO-5***; KCCQ ^†††^ ) Functional capacity	The intervention had no impact on mortality risk. All HRQoL scales improved consistently and significantly in the IG ^‡^ at 12 months compared to the CG ^†^ (all p < 0.01)
Brouwers, et al. ^( 35 )^ 2021 Netherlands	Individuals in rehabilitation (phase 2) for CAD ^‡‡‡^ n: 300 CG ^†^ : 147 | IG ^‡^ : 153) Follow-up: 12 months	Telerehabilitation, whose program included 6 group exercise sessions, weekly video consultations carried out by physiotherapists until the completion of individual or program goals, in addition to weekly telemonitoring	App: Vital signs monitoring HR monitor** Accelerometer Physical training module Weekly video consultations	Center-based cardiac rehabilitation	HRQOL ^||^ (EQ-5D ^‡‡^ 5-levels; EQ-VAS ^§§§^ ; MacNew Heart Disease Health-Related Quality of Life Questionnaire) Healthcare costs	Patients in IG ^‡^ and CG ^†^ had comparable HRQoL ^||^ (mean difference in EQ-5D-5L ^‡‡^ : −0.004; p = 0.82; mean difference in EQ-VAS ^§§§^ : −0.001). Although intervention costs were higher, there were no differences in total cardiac healthcare costs between the two groups (€4,787 vs. €5,507, p = 0.36)
Maddison, et al. ^( 36 )^ 2021 New Zealand	Adults with SCA ^¶^ n:306 (CG ^†^ :153| IG ^‡^ :153) Follow-up: 13 months	Automated SMS ^§§^ program called Text 4 HartII, promoting self-management and health education	Sending personalized SMS ^§§^ Telephone calls to administer questionnaires	Usual outpatient care, without details	Medication adherence (Morisky Medication Adherence Scale) HRQoL ^||^ (EQ-5D ^‡‡^ )	Medication adherence in three classes was lower in the IG ^‡^ compared with the CG ^†^ , both at 24 weeks (56.8% vs 68.6%) and at 52 weeks (67.9% vs 54.2%). Self-reported medication adherence scores reflected this trend at 52 weeks
Collins, et al. ^( 37 )^ 2021 The United States of America	Individuals diagnosed with HF* n:491 (CG ^†^ :245 | IG ^‡^ :246) Follow-up: 12 months	Usual care and personalized discharge plan, telephone self-care coaching, with intervention consisting of home visit within 7 days of discharge and telephone self-care training	Telephone calls Telehealth consultation	Usual care consisting of a structured discharge process, including reconciliation and medication prescription; and medical consultation for follow-up	Death associated with cardiovascular disease Events related to HF* HRQoL ^||^ (KCCQ ^†††^ )	There was no significant difference between the groups in mortality, HF-related events*, and changes in KCCQ ^†††^ summary score at 90 days. Although the KCCQ ^†††^ summary score was higher in the IG ^‡^ , the difference was not statistically significant (95% confidence interval -1.9 to 7.2; P = 0.25)
Clays, et al. ^( 38 )^ 2021 Belgium and Italy	Individuals diagnosed with HF* n:56 (CG ^†^ :22 | IG ^‡^ :34) Follow-up: 6 months	Home telemonitoring system consisting of a combination of monitoring devices and a mobile application, designed to provide comprehensive support for patient health management	HeartMan System: Wearable HR** and BP monitor ^¶¶^ Scale Pill organizer Smartphone with HeartMan mobile app, with features for physical management, psychological support and HF* education Operational telephone support service	Usual care with standard treatment according to clinical guidelines provided by the cardiologist, general practitioner and IC nurse*	HRQoL ^||^ (MLHFQ ^||||||^ ) Exercise capacity (6MWT ^¶¶¶^ ) Perception of illness and mental health (Brief Illness Perception. Questionnaire) Self-care (Self-Care of Heart Failure Index)	In IG ^‡^ , there was a significant reduction in depression and anxiety (p < 0.001), while in CG ^†^ the need for sexual counseling decreased (p < 0.05). Only in IG ^‡^ , self-care increased (p < 0.05) and sexual problems decreased (p < 0.05), but there was no significant impact on HRQoL ^||^ confidence in self-care, perception of illness or exercise capacity between the groups.
Batalik, et al. ^( 39 )^ 2020 Czech Republic	Individuals with cardiovascular disease undergoing cardiac revascularization n: 56 (CG ^†^ : 28 | IG ^‡^ : 28) Follow-up: 3 months	Telerehabilitation, preceded by supervised outpatient training	App: Vital signs monitoring HR** monitor Physical training module Weekly telephone reassessment	Center-based cardiac rehabilitation	Physical fitness (Cardiopulmonary Exercise Test) HRQoL ^||^ (SF ^||||^ -36) Adherence to training	Both groups showed significant improvements in physical fitness (P < 0.001), with similar adherence between them. HRQoL ^||^ improved significantly in both groups, with no significant difference between them (P < 0.01)
Lunde, et al. ^( 40 )^ 2020 Norway	Individuals undergoing rehabilitation (phase 2) for CAD ^‡‡‡^ n: 300 CG ^†^ : 147 |IG ^‡^ :153) Follow-up: 12 months	Individualized monitoring via app, with goal setting, reminders, progress assessment and personalized feedback	App: Behavioral guidelines (physical activity and diet) Healthy habits Setting individual goals Reminders Individual feedback	Usual care was not detailed	Difference in peak VO _2_^††^ Exercise performance Body weight Resting BP ^††^ Exercise habits HRQoL ^†^ (HeartQoL****) Health status (EQ-5D ^‡‡^ 5-levels)	In IG ^‡^ , there was a significant difference in peak VO _2_^††^ compared to CG ^†^ (mean difference of 2.2 ml/kg/min, 95% confidence interval 0.9–3.5, p = 0.001). In addition, IG ^‡^ showed better exercise performance, exercise habits and self-perception of goal achievement, compared to CG ^†^ . No significant differences were found between the groups in blood pressure, HRQoL ^||^ and health status.
Piotrowicz, et al. ^( 41 )^ 2020 Poland	Individuals diagnosed with HF* n:850 (CG ^†^ :425|IG ^‡^ :425) Follow-up: 14 to 26 months	Telerehabilitation started with hospital training, followed by home training. It included medical supervision, education, and personalized exercise planning for each patient.	Monitoring center, with cell phone availability for: Recording of vital signs and clinical data Individualized physical training Educational program Portable ECG ^††††^ BP monitor ^¶¶^ Scale Cardioverter defibrillator monitor (only for patients with the implant)	Usual care included regular clinical assessments, participation in rehabilitation programs, remote monitoring of implantable cardiac devices, and advice on lifestyle changes and self-management according to standard clinical guidelines.	Percentage of out-of-hospital survival Mortality Change in cardiopulmonary exercise test duration HRQoL ^||^ (SF ^||||^ -36)	There were no significant differences in mortality rate (12.5% vs 12.4%, respectively; hazard ratio, 1.03 [95% confidence interval, 0.70-1.51]) or hospitalization (mean [SD] days, 91.9 [19.3] vs 92.8 [18.3], respectively; P = .74) between groups. However, IG ^‡^ showed significant benefits in terms of improved maximal oxygen uptake and HRQoL ^||^ (SF score ^||||^ -36, 1.58 [95% confidence interval, 0.74-2.42] vs 0.00 [95% confidence interval, -0.84 to 0.84]; P = .008), and it was well tolerated with no serious adverse events during exercise.
Ávila, et al. ^( 42 )^ 2020 Belgium	Individuals with CAD ^‡‡‡^ n:90 (CG ^†^ usual care:30 | CG ^†^ rehabilitation:30 | IG ^‡^ : 30) Follow-up: 3 months	Telerehabilitation program associated with telemonitoring and individualized exercise prescription	App Wearable (watch) Feedback by phone or email	Center-based rehabilitation with outpatient training Usual care: encouraged to maintain a physically active lifestyle and invited for follow-up appointments	Cardiorespiratory fitness (VO ^2††^ peak). Physical activity Traditional cardiovascular risk factors HRQoL ^||^ (SF ^||||^ -36)	All groups maintained high scores for all HRQoL ^||^ parameters, with no significant differences between groups (p = 0.70). Exercise capacity and secondary outcomes were preserved in all three groups over time (p > 0.05 for all), regardless of intervention (p > 0.05 for all).
Cáceres, et al. ^( 43 )^ 2020 The United States of America	Individuals diagnosed with AF ^‡‡‡‡^ or Atrial Flutter n:238 (CG ^†^ :123|IG ^‡^ :115) Follow-up: 6 months	iHEART intervention for telemonitoring and guidance via SMS ^§§^	Use of iPhone with AliveCor ^®^ Kardia mobile ECG ^††††^ system for remote cardiac monitoring Sending SMS ^§§^ with guidance on AF management ^‡‡‡‡^ and lifestyle.	Usual care with standard treatment in accordance with current guidelines	AF recurrence ^‡‡‡‡^ HRQoL ^||^ (SF ^||||^ -36; EQ-5D-5L ^‡‡^ ; Atrial Fibrillation Effect on Quality of Life) AF symptom severity ^‡‡‡‡^ (Atrial Fibrillation Severity Scale)	Improvement in both groups from baseline to follow-up in AF Effect Scale scores ^‡‡‡‡^ on HRQoL ^||^ (p < 0.05), with greater impact in IG ^‡^ . There were no statistically significant differences in HRQoL ^||^ or severity of AF symptoms ^‡‡‡‡^ between groups.
Cichosz, et al. ^( 44 )^ 2019 Denmark	Individuals diagnosed with HF* n:299 (CG ^†^ :154|IG ^‡^ :145) Follow-up: 12 months	Telekits intervention, with a central clinical system that included vital signs monitoring, nursing assessment, telephone contacts for management and referral to medical appointments, if necessary	Tablets Digital BP ^¶¶^ monitors Scales Telephone contact	Rehabilitation, dietary counseling, coaching, medication monitoring, risk factor screening, and lifestyle change discussions	HRQoL ^||^ (SF ^||||^ -36): physical and mental score HF*-specific questionnaire score (KCCQ ^†††^ )	The IG ^‡^ showed significant improvement in mental scores compared to the CG ^†^ (p < 0.01). There was no significant effect on the improvement of the physical score (SF ^||||^ -36) or on the quality of life related to HF* (KCCQ ^†††^ )
Mizukawa, et al. ^( 45 )^ 2019 Japan	Individuals diagnosed with HF* n: 59 (CG ^†^ :19| IG ^‡^ 1:20| IG ^‡^ 2: 20) IG ^‡^ 1: Collaborative management IG ^‡^ 2: Education for self-management Follow-up: 24 months	Monitoring and self-management system, with daily recording of vital signs, doctor visits, monthly self-management education sessions and remote telephone monitoring by a nurse for the IG ^‡1^	HR** monitor BP ^¶¶^ monitor Weight monitoring Educational intervention Telephone management via nurse, only for IG ^‡1^	Physician visits every 2-4 weeks, an education session at discharge, and guidance on daily recording of weight, BP ^¶¶^ , and pulse in a self-management notebook	HRQoL ^||^ (MLHFQ ^||||||^ ) Self-efficacy (Chronic Disease Self-Efficacy Scale) Self-care (European Heart Failure Self-care Behaviour ScaleS) Readmission due to HF* All-cause mortality	HRQoL ^||^ significantly improved in the IG ^‡1^ group compared with the CG ^†^ at 18 and 24 months (P < 0.05). The IG ^‡1^ group also showed significant improvements in self-efficacy and self-care (P < 0.01), and had lower rates of rehospitalization (20.0% vs. 57.9% in the CG ^†^ ) with greater survival without readmission (P = 0.020).
Pekmezaris, et al. ^( 46 )^ 2019 The United States of America	Black and Hispanic individuals from underserved communities with a primary diagnosis of HF* n: 104 (CG ^†^ :58 | IG ^‡^ :46) Follow-up: 3 months	Self-monitoring via telehealth, focused on daily self-management and weekly telehealth visits with intensive and continuous support from a nurse	HR** monitor BP ^¶¶^ monitor Weight monitoring Oxygen saturation monitor Weekly video consultation with a nurse	Comprehensive outpatient management based on usual care in an outpatient setting, with adherence to American Heart Association guidelines	Hospitalization and emergency service utilization HRQoL ^||^ (MLHFQ ^||||||^ ) Anxiety and depression (Patient Health Questionnaire -4)	The results indicated that there was no significant difference between IG ^‡^ and CG ^†^ in emergency room visits (Relative Risk = 1.37, confidence interval = 0.83–2.27), hospitalizations (Relative Risk = 0.92, confidence interval = 0.57–1.48) or length of hospital stay (IG ^‡^ = 0.54 vs. CG ^†^ = 0.91). Both groups had an increase in HRQoL ^||^ values, with no statistical differences
Peng, et al. ^( 47 )^ 2018 China	Individuals diagnosed with HF* and their caregivers n:98 (CG ^†^ :49 | IG ^‡^ :49) Follow-up: 4 months	Telerehabilitation, a telehealth physical training program with exercise sessions monitored by physiotherapists and regular follow-up by cardiac nurses via telephone or SMS ^§§^	Telehealth fitness training program Educational brochure Exercise sessions, with video supervision Follow-up with nurses via phone calls or instant messages (Wechat)	Usual care was based on an educational session at hospital discharge and regular follow-up visits to the clinic. Patients in this group did not receive specific instructions on physical exercise.	HRQoL ^||^ (MLHFQ ^||||||^ ) Distance covered in the 6MWT ^¶¶¶^ Physiological measures Anxiety and depression (Hospital Anxiety and Depression Scale)	The IG ^‡^ demonstrated statistically significant improvements in HRQoL ^||^ (Fb = 8.272, P = 0.005), with a maintenance up to 4 months after the post-test (Fin = 79.73, P = 0.000) and in the distance walked in the 6MWT ^¶¶¶^ compared to the CG ^†^ in the post-test. No significant improvements were observed in relation to the other parameters
Wagenaar, et al. ^( 48 )^ 2018 Netherlands	Individuals diagnosed with HF* n:450 (CG ^†^ :150 | IG ^‡^ site:150 | IG ^‡^ e-Vita:150) Follow-up: 12 months	The intervention consisted of a website containing educational health content. In addition, IG ^‡^ e-Vita participants followed an e-health care plan with telemonitoring and personalized adjustments.	Website Nursing guidelines e-Vita platform Regular recording of vital parameters (weight, BP ^¶¶^ and HR**) Alerts triggered in case of values outside the pre-established limits Updates on comorbidities and medications Monthly reminders by email for monitoring and adherence to the care plan	Usual care from nine HF* outpatient teams, including routine consultations with a cardiologist and an HF* nurse, on average four times per year.	Patient self-care (European Heart Failure Self-Care Behavior Scale) HRQoL ^||^ (MLHFQ ^||||||^ ) Disease-specific knowledge (Dutch Heart Failure knowledge Scale) Patient satisfaction with HF* care Mortality	After 3 months, IG ^‡^ showed better self-care compared to CG ^†^ (IG ^‡^ site 73.5 vs. 70.8 and IG ^‡^ e-Vita 78.2 vs. 70.8, respectively). Furthermore, after 3 and 6 months, there were significant differences in HRQoL ^||^ between IG ^‡^ e-Vita and CG ^†^ (median IG ^‡^ e-Vita 19.0 vs. CG ^†^ 22.8, p = 0.029 and IG ^‡^ e-Vita 21.0 vs. CG ^†^ 24.0, p = 0.003), respectively. These differences diminished over time, with no differences after 1 year.
Guo, et al. ^( 49 )^ 2017 China	Individuals with AF ^‡‡‡‡^ n: 209 (CG ^†^ :96 | IG ^‡^ :113) Follow-up: 3 months	Self-management system based on a mobile application, offering clinical decision support, educational programs, health monitoring, and structured follow-up	Clinical decision support application: Automatic calculation of risk scores Educational programs Self-care engagement components Structured follow-up components Personal health record	Usual care was not described	Patient knowledge about AF ^‡‡‡‡^ HRQoL ^||^ (EQ-5D ^‡‡^ ) Medication adherence (Pharmacy Quality Alliance) Usability, feasibility and acceptability of the application	Over 90% of patients reported that the app was easy to use and useful, associated with significant improvements in knowledge (p < 0.05). Medication adherence and satisfaction with anticoagulants were significantly better in the IG ^‡^ compared to the CG ^†^ (p < 0.05). HRQoL ^||^ scores significantly improved with the app, with reductions in anxiety and depression (p < 0.05)
Hwang, et al. ^( 50 )^ 2017 Australia	Individuals diagnosed with HF* n:53 (CG ^†^ :29 | IG ^‡^ :24) Follow-up: 6 months	Telerehabilitation, with a program carried out twice a week with video consultations, supervision by a physiotherapist, real-time exercises and educational sessions and remote support	Telerehabilitation via video consultation platform Interaction between the physiotherapist and participants, feedback and modifications of therapeutic plans Educational slides with embedded audio files Online group discussions Collaborative design Chat functions Equipment manual with written and pictorial instructions	Traditional center-based rehabilitation	HRQoL ^||^ (MLHFQ ^||||||^ ; ^‡‡^ EQ-5D) Distance covered in the 6MWT ^¶¶¶^ Satisfaction (Client Satisfaction Questionnaire) Rate of adverse events	The analyses revealed no significant differences between the groups regarding HRQoL ^||^ , indicating a similarity in the results. Both groups showed statistically significant improvements in their HRQoL ^||^ , which were maintained during the follow-up period. Regarding the distance covered in the 6MWT ^¶¶¶^ , no significant differences were observed between the groups, with a mean difference of 15 meters (95% confidence interval –28 to 59).
Jayaram, et al. ^( 51 )^ 2017 The United States of America	Individuals diagnosed with HF* n:1521 (CG ^†^ :765 | IG ^‡^ :756) Follow-up: 6 months	Tele-HF telemonitoring program based on telephone calls	Educational materials Scale Daily phone calls with questionnaires Medical feedback, if needed Reminders	Usual care was not detailed, cited as standard recommendations from guidelines for the treatment of HF*, with educational materials and provision of a scale if necessary	HRQoL ^||^ (KCCQ ^†††^ )	During the 6-month follow-up, patients in IG ^‡^ had a mean overall KCCQ ^†††^ summary score 2.5 points higher (95% confidence interval = 0.38, 4.67; p = 0.02) than those receiving usual care. This difference was driven primarily by improvements in symptoms (3.5 points; 95% confidence interval = 1.18, 5.82; p = 0.003) and social functioning (3.1 points; 95% confidence interval = 0.30, 6.00; p = 0.03).
Piotrowicz, et al. ^( 52 )^ 2020 Poland	Individuals diagnosed with HF* n:111 (CG ^†^ :34| IG ^‡^ : 77) Follow-up: 2 months	Telerehabilitation program associated with telemonitoring and individualized exercise prescription, based on Nordic walking	Telerehabilitation and remote monitoring equipment: Mini EHO Device BP Monitor ^¶¶^ Scale ECG ^††††^ Cell phone Questionnaires Telephone support (psychological support and additional instructions)	Usual care, without formal prescription of physical training or supervised rehabilitation. Recommendations for lifestyle changes and self-management according to the European Society of Cardiology guidelines	Functional capacity (VO _2_^††^ peak) HRQoL ^||^ (SF ^||||^ -36) Extended assessment of rehabilitation effectiveness Safety and adherence to training	In IG ^‡^ , a significant improvement in functional capacity was observed (p = 0.0001) with positive repercussions on HRQoL ^||^ , without statistical differences. All participants completed the rehabilitation program, with high adherence to home Nordic walking training, with 94.7% of patients considered adherent
Bekelman, et al. ^( 53 )^ 2015 The United States of America	Individuals diagnosed with HF* n:392 (CG ^†^ :193| IG ^‡^ :199) Follow-up: 12 months	The Patient-Centered Disease Management Program combined multidisciplinary collaborative care, telemonitoring, and joint management of HF* and depression	Multidisciplinary collaborative care Screening and treatment of depression Telemonitoring and patient self-care support: BP ^¶¶^ and HR** monitor Weight monitoring and self-reported symptoms Medication reminders Education material on HF* and depression Dietary counseling	Follow-up with usual care healthcare team, information at enrollment visit describing self-care for HF* and a scale if needed, physicians notified if depressive symptoms developed	HRQoL ^||^ (KCCQ ^†††^ ) Depressive symptoms (PHQ-9 ^§§§§^ ) Hospitalizations Mortality	There were no significant differences in baseline characteristics between the intervention and usual care groups. After 1 year, both groups showed similar improvement in KCCQ ^†††^ scores (mean change 13.5 points, p = 0.97). The intervention was associated with fewer deaths at 1 year (4.3% vs 9.6%, p = 0.04)
Frederix, et al. ^( 54 )^ 2015 Belgium	Individuals with CAD ^‡‡‡^ in cardiac rehabilitation or HF* n: 140 (CG ^†^ : 70 | IG ^‡^ :70) Follow-up: 6 months	Telerehabilitation, consisting of telemonitoring and telecoaching, added to the conventional 12-week center-based cardiac rehabilitation program.	Remote monitoring of physical activity Accelerometer Remote coaching via website Guidance on smoking cessation, diet and physical activity Feedback via email or SMS ^§§^	Traditional center-based rehabilitation	Maximum aerobic capacity (VO _2_^††^ peak) Daily physical activity Glycated hemoglobin, glycemic control and lipid profile HRQoL ^||^ (HeartQoL****)	Patients in IG ^‡^ showed a significant increase in mean peak VO _2_^††^ from baseline (mean 22.46) to 24 weeks (mean 24.46, P<0.01), while in CG ^†^ there was no significant change. Furthermore, self-reported physical activity and global HRQoL ^||^ score improved more in IG ^‡^ compared to CG ^†^ at 24 weeks (P=0.01)
Maddison, et al. ^( 55 )^ 2015 New Zealand	Individuals with ischemic heart disease n: 171 (CG ^†^ :86 | IG ^‡^ :85) Follow-up: 6 months	HEART Program, which consisted of exercise prescription and behavioral support via SMS ^§§^ and internet, aiming to increase physical activity in patients	Prescription of regular exercise Provision of behavior change strategies Website with information about the disease and possibility of self-monitoring of progress Daily SMS ^§§^ sending	Usual care was not described	Maximal aerobic capacity (VO _2_^††^ peak) Self-reported physical activity (International Physical Activity Questionnaire) Self-efficacy and motivation for exercise HRQoL ^||^ (SF ^||||^ -36 version 2; EQ-5D ^‡‡^ )	There was a significant increase in self-reported leisure-time physical activity (p = 0.05) and walking (p = 0.02) in IG ^‡^ . There were also significant improvements in self-efficacy to be active and the general health domain of the SF ^||||^ -36 in IG ^‡^ , with a difference of 2.1, 95% confidence interval: 0.1, 4.1; p = 0.03) at 24 weeks for HRQoL ^||^
Piotrowicz, et al. ^( 56 )^ 2015 Poland	Individuals diagnosed with HF* n: 152 (CG ^†^ :75 | IG ^‡^ :77) Follow-up: 2 months	Telerehabilitation, based on walking, using a telemonitoring system and supervised training	Telerehabilitation with pre-programmed training sessions for each individual Telemonitoring Sending of resting ECG ^††††^ and answering a health questionnaire before physical activity Psychological support	Traditional center-based rehabilitation	HRQOL ^||^ (SF ^||||^ -36)	Total HRQoL ^||^ was improved in both groups, but was not significant. IG ^‡^ had an improvement mainly in the mental categories and CG ^†^ improved their general physical well-being.
Kraal, et al. ^( 57 )^ 2014 Netherlands	Individuals with CAD ^‡‡‡^ who entered cardiac rehabilitation after hospitalization for AMI ^||||||||^ , unstable angina or revascularization (low to moderate risk) n: 55 (CG ^†^ :26 | IG ^‡^ :29) Follow-up: 3 months	Telerehabilitation, which began with monitored training sessions, goal setting and motivational interviews, with telephone feedback from the physiotherapist	Telerehabilitation Telemonitoring with wearable HR monitor** App Feedback by phone	Traditional center-based rehabilitation	VO _2_^††^ peak HRQoL (MacNew Heart Disease Health-Related QoL) Adherence to training	Significant improvement in peak VO _2_^††^ consumption (CG ^†^ : 10% and GI ^‡^ : 14% respectively) in both groups, with no significant difference between groups. HRQoL ^||^ improved significantly in both groups, with no differences between them.
Varnfield, et al. ^( 58 )^ 2014 Australia	Individuals after AMI ^||||||||^ n: 120 (CG ^†^ : 60| IG ^‡^ :60) Follow-up: 6 months	Telerehabilitation via Cardiac Rehabilitation Care Assessment Platform (CAP-CR), based on exercise prescription and delivery of educational content	CAP-CR Platform: Health monitoring Exercise monitoring SMS ^§§^ and audio files with motivational and educational content Web portal Weekly consultations with mentors	Traditional center-based rehabilitation	Adherence, adherence and completion of the cardiac rehabilitation program Biomedical risk factors (BP ^¶¶^ , HR**, weight, among others) HRQoL ^||^ (EQ-5D ^‡‡^ )	The IG ^‡^ had significantly higher adherence (80% vs 62%) and completion (80% vs 47%) rates than the CG ^†^ (p<0.05). Both groups improved significantly in the 6MWT ^¶¶¶^ , which was maintained at 6 months. The IG ^‡^ also showed positive effects on HRQoL ^||^ (EQ-5D ^‡‡^ : median 0.84 to 0.92)
Blum, et al. ^( 59 )^ 2014 The United States of America	Individuals diagnosed with HF* n: 206 (CG ^†^ : 102|IG ^‡^ :104) Follow-up: 12 months	Vital signs telemonitoring system, with nursing monitoring and patient contact Send feedback Translation results available	Philips Electronics E-care system for remote data monitoring; Scale BP ^¶¶^ Monitor ECG ^††††^	Easy access to routine specialist care, with outpatient appointments and access to telephone numbers as appropriate	HRQoL ^||^ (SF ^||||^ -36; MLHFQ ^||||||^ ) Medical costs Rehospitalization within 30 days Mortality	HRQoL ^||^ scores in both groups improved over the year (p<0.001), but there was no significant difference between the groups. There were no differences in mortality (p=0.575), readmission rate (p=0.627), or payments for hospitalizations and emergency department visits between the groups.
Cui, et al. ^( 60 )^ 2013 Canada	Individuals diagnosed with HF* n:174 (CG ^†^ :55 | IG ^‡^ Telephone:61 | IG ^‡^ Telemonitoring:58) Follow-up: 12 months	Health Lines Program, consisting of: nursing care with health guidance for self-management and health education tools. In addition to home monitoring for IG ^‡^ patients Telemonitoring	Nursing phone calls BP ^¶¶^ monitors Scales Health education tools	Usual care was not described	Costs and health care HRQoL ^||^ (SF ^||||^ -36) Self-care (Revised Self-Care Behavior scale) Satisfaction (Client Satisfaction Questionnaire)	Both interventions were more effective and less expensive than standard care. There was a significant improvement in self-care behavior and HRQoL ^||^ (p<0.05), with statistically significant higher utility scores in the intervention groups.
Hawkes, et al. ^( 61 )^ 2012 Australia	Individuals diagnosed with AMI ^||||||||^ n:430 (CG ^†^ :215 | IG ^‡^ :215) Follow-up: 6 months	Telephone coaching on managing heart disease risk factors “My Heart My Life” educational resource	Telemedicine or support telephone lines	“My Heart My Life” educational resource and informative physical newsletter every three months	HRQoL ^||^ (SF ^||||^ -36) Physical activity (Active Australia Survey) Satisfaction Risk factors for heart disease	At 6 months, the IG ^‡^ demonstrated higher HRQoL ^||^ scores compared to the CG ^†^ in the mental (95% confidence interval 0.5 to 4.9; p = 0.02), physical (95% confidence interval 0.1 to 4.5; p = 0.04) and emotional (95% confidence interval 0.2 to 5.2; p = 0.03) components. In addition, the IG ^‡^ were more likely to be sufficiently active (p = 0.02) and maintain a healthy weight (p = 0.05) compared to the CG ^†^ .
Seto, et al. ^( 62 )^ 2012 Canada	Individuals diagnosed with HF* n: 100 (CG ^†^ :50 | IG ^‡^ :50) Follow-up: 6 months	Telemonitoring system and beyond standard service	Telemonitoring system: Daily weight and BP ^¶¶^ and ECG ^††††^ records Symptom questionnaire on cell phone Sending messages Website Reminder phone call Sending alert emails to the cardiologist Contacting the patient by phone	Regular care, including scheduled clinic visits and HF* education during appointments. They also had access to telephone support but did not receive study-specific interventions beyond this usual care	Brain natriuretic peptide levels Self-Care of Heart Failure Index HRQoL ^||^ (MLHFQ ^||||||^ )	The IG ^‡^ showed a significant improvement in HRQOL ^||^ (p = 0.05), and greater maintenance of self-care (p = 0.03), compared to the CG ^†^ . Both groups had improvements in brain natriuretic peptide levels, self-care management, and ventricular ejection fraction.
Blasco, et al. ^( 63 )^ 2012 Spain	SCA ^¶^ survivors n: 203 (CG ^†^ :101 | IG ^‡^ :102) Follow-up: 12 months	Telemonitoring and sending text messages with recommendations	Web app for sending text messages Mobile phone Sending recommendations via mobile phone BP monitor and glucometer	Three consultations with a cardiologist; written and verbal recommendations on prevention of cardiovascular diseases.	HRQoL ^||^ (SF ^||||^ -36) Anxiety (State - Trait Anxiety Inventory) Improvement of cardiovascular risk factors	There were no significant differences between groups in HRQOL ^||^ scores at baseline or end of the study. However, in the “physical health” domain of the SF ^||||^ -36, there was a 2.8-point increase in IG ^‡^ (p = 0.011). IG ^‡^ were more likely to experience an improvement in their cardiovascular risk factor profile (relative risk 1.4; 95% confidence interval 1.1-1.7) than patients in CG ^†^ (p = 0.010).
Koehler, et al. ^( 64 )^ 2011 Germany	Individuals diagnosed with HF* n:710 (CG ^†^ :356|IG ^‡^ :354) Follow-up: 12 months	Remote self-management system based on telemedicine	Remote telemonitoring system, consisting of: Personal digital assistant ECG ^††††^ Scale BP ^¶¶¶^ monitor Telemedicine center, with 24-hour medical support	Usual care was not detailed, cited as standard recommendations of guidelines for the treatment of HF*	Mortality Hospitalizations Depression (PHQ-9 ^§§§§^ ) HRQoL ^||^ (SF ^||||^ -36)	The study results showed that the intervention had no significant impact on all-cause mortality or cardiovascular mortality or hospitalization for HF* compared with CG ^†^ . Furthermore, there was a significant improvement in the SF ^||||^ -36 physical functioning score in IG ^‡^ throughout the study period (p<0.05).
Baker, et al. ^( 65 )^ 2011 The United States of America	Individuals diagnosed with HF* n: 605 (CG ^†^ : 303 | IG ^‡^ : 302) Follow-up: 1 month	Telecoaching for self-management, initiated by 40-minutes educational sessions on HF* management	Follow-up phone calls and health guidance and education	40-minute educational session on the management of HF*, other usual care was not detailed	HRQL ^||^ (HFSS ^¶¶¶¶^ ) Knowledge (Improving Chronic Illness Care Evaluation)	There were no significant changes in HRQOL ^||^ of the CG ^†^ from baseline to the end of follow-up, but in the IG ^‡^ there was a difference from 58.6 (±22.2) at baseline to 65.3 (±22.4) at the end of 30 days (p = 0.001). The IG ^‡^ showed a greater increase in general knowledge (0.70 vs. 0.30, p = 0.008) and in self-efficacy (0.4 vs. 1.0, p = 0.006)
Copeland, et al. ^( 66 )^ 2010 The United States of America	Individuals diagnosed with HF* n:458 (CG ^†^ :238|IG ^‡^ :220) Follow-up: 12 months	Disease management program, with scheduled phone calls by nurses, providing education, coaching for behavior change and symptoms monitoring	Scheduled phone calls from nurses Individualized self-management plan 24-hour nurse advice line Fax alerts to physicians about signs of decompensation	Usual care was not described	HRQoL ^||^ (SF ^||||^ -8) Health costs Survival Adherence and satisfaction with care	After one year, there were no significant clinical differences between groups. HF*-related costs and overall costs were higher in IG ^‡^ . This group also reported better adherence to weight monitoring and exercise recommendations. There were no differences in HRQoL ^||^ , use of hospitalization, or survival between groups.
Balk, et al. ^( 67 )^ 2008 Netherlands	Individuals diagnosed with HF* n: 214 (CG ^†^ :113| IG ^‡^ :101) Follow-up: 9.6 months	Motiva ^®^ Health Education and Self-Management System	TV channel with educational material Medication reminders Health-related questionnaires Motivational SMS ^§§^ Telemonitoring of BP ^¶¶^ and weight Telephone contact by nurses	Standard follow-up provided by cardiologists and HF* nurses as per local practice	Days of hospitalization/year HRQoL ^||^ (SF ^||||^ -36; MLHFQ ^||||||^ ) Knowledge of the disease Self-care (European Heart Failure Self-care Behaviour Scale)	There were no significant differences for the primary outcomes, HRQoL ^||^ or self-care behavior. However, there was a greater increase in knowledge about HF* in the IG ^‡^ compared to the CG ^†^ (p<0.001)
Schwarz, et al. ^( 68 )^ 2008 The United States of America	Individuals diagnosed with HF* or caregivers n:102 (CG ^†^ :51| IG ^‡^ :51) Follow-up: 3 months	Remote monitoring system, associated with regular monitoring by healthcare professionals.	Telemonitoring system linked to the patient’s telephone line: Daily weight measurement Questionnaire on symptoms, medication adherence and sodium intake Telephone contact with caregiver, if necessary	Usual care was not detailed, being mentioned only as post-discharge care.	New hospitalizations Emergency visits Costs HRQoL ^||^ (MLHFQ ^||||||^ )	The pilot study found no statistically significant differences in IG ^‡^ regarding hospital readmissions, emergency department visits, costs, or risk of hospital readmission. HRQOL ^||^ improved significantly in both groups at the 90-day follow-up visit (p<0.0001)
Wakefield, et al. ^( 69 )^ 2008 The United States of America	Individuals diagnosed with HF* n:148 (CG ^†^ :49|IG ^‡^ Video:52| IG ^‡^ Phone:47) Follow-up: 12 months	Telehealth program, based on telephone or video follow-up, symptom monitoring and health education	Symptom review checklist Daily monitoring of weight, BP ^¶¶^ and ankle circumference Telephone contact Video call contact Behavioral skills training Strategies to maximize self-monitoring and self-efficacy Review and reinforcement of discharge plans	Standard hospital discharge guidance from the health service and telephone contact with the case manager nurse, if necessary	Number of readmissions Time to first readmission Emergency unit visits Survival HRQoL ^||^ (MLHFQ ^||||||^ )	There was no difference in days of hospitalization or emergency department visits among the 3 groups. For all groups, HRQoL ^||^ scores improved over time (F = 8.90, p = 0.0002). The magnitude of change was greatest in IG ^‡^ Phone, followed by IG ^‡^ Video and CG ^†^
Woodend, et al. ^( 70 )^ 2008 Canada	Individuals diagnosed with HF* or angina n:249 (CG ^†^ :125|IG ^‡^ :124) Follow-up: 12 months Send feedback	Home telemonitoring program consisting of video consultations, monitoring of vital parameters, telephone support and structured educational content	Home monitoring equipment: Scales BP ^¶¶^ monitor 12-lead ECG ^††††^ Video consultations with a nurse Electronic data logging 24-hour nursing advice line	Usual treatment care provided to patients with angina or HF* who are discharged from hospital, without further details	Functional status (MLHFQ ^||||||^ ; Seattle Angina Questionnaire) HRQoL ^||^ (SF ^||||^ -36) Use of health resources (hospitalizations, days of hospitalization, emergency room visits)	HRQOL ^||^ was better in HF* patients in the IG ^‡^ compared to the CG ^†^ in five of the eight SF ^||||^ -36 subscales at 3 months (p < 0.05). The most significant differences in HRQoL ^||^ between the two groups were detected at this time point. Patients in both groups demonstrated significant improvements in HRQOL ^||^ over time in all SF ^||||^ -36 subscales.
López Cabezas, et al.( 71 ) 2006 Spain	Individuals diagnosed with HF* n: 134 (CG ^†^ : 64 | IG ^‡^ :70) Follow-up: 12 months	Pharmacist-led health guidance program with telephone support for questions or problems	Written and audiovisual educational material on the symptoms and pathogenesis of HF*; Telephone calls by the pharmacist	Regular consultations with a cardiologist and pharmacist	Readmissions Treatment adherence HRQoL ^||^ (EQ-5D ^‡‡^ ) Patient satisfaction	In IG ^‡^ , there was greater adherence to treatment (85.0 vs. 73.9%), fewer days of hospitalization (CG ^†^ 9.6 vs. IG ^‡^ 5.9) and greater satisfaction (p = 0.026) when compared to CG ^†^ . There was a significant reduction in hospital readmissions in IG ^‡^ (54% at the beginning vs. 32% at the end), but there were no significant differences in HRQoL ^||^ .
Riegel et al. ^( 72 )^ 2006 The United States of America	Individuals diagnosed with HF* n: 134 (CG ^†^ : 65 | IG ^‡^ : 69) Follow-up: 6 months	Telephone case management, focusing on education, monitoring and guidance, as well as reporting and contact with medical staff	Decision support software (At Home with Heart Failure) Monthly mailing of printed educational material to patients Telephone support	Non-standardized usual care based on education on HF management*	Hospitalizations, hospital days and acute care costs (by HF* and all causes) Multiple readmissions All-cause mortality HRQoL ^||^ (EQ-5D ^‡‡^ )	No significant differences were found between groups in readmission rate, hospitalization days, cost of care, mortality, HRQoL ^||^ or depression
Benatar, et al. ^( 73 )^ 2003 The United States of America	Individuals diagnosed with HF* n:216 (CG ^†^ :108| IG ^‡^ :108) Follow-up: 3 months	Home telemonitoring system, based on self-management devices with telephone assessments, with definition of individual clinical goals	Home monitoring devices to measure weight, BP ^¶¶^ , HR** and oxygen saturation level Remote management team with contact via telephone calls	Usual care consisted of health monitoring via the center and home nursing visits	HRQoL ^||^ (MLHFQ ^||||||^ | 70-item Quality of Life Index – Cardiac) Anxiety and depression (HADS*****) Self-efficacy (Heart Failure Self-Efficacy scale 30) Hospitalizations Health costs	The IG ^‡^ group had fewer HF* readmissions (13 vs. 24; P≤0.001), with a shorter hospital length of stay (49.5 vs. 105.0 days; P≤0.001) and lower hospital costs at 3 months ($65,023 vs. $177,365; p≤0.02). Both groups experienced an improvement in HRQoL ^||^ after the intervention
Barnason, et al. ^( 74 )^ 2003 The United States of America	Individuals with ischemic HF* undergoing myocardial revascularization n:35 (CG ^†^ :17| IG ^‡^ :18) Follow-up: 3 months	Health Buddy: Symptom assessment and education on post-revascularization recovery, symptom management, functioning, and adherence to CAD ^‡‡‡^ risk factor modification	Telecommunication device connected to the patient’s phone (Health Buddy) Website where the collected data is stored	Education and counseling on post-surgical recovery care, self-care and modification of CAD ^‡‡‡^ risk factors, prescription of home exercises, provided to all patients undergoing revascularization prior to hospital discharge	Self-efficacy (Barnason Efficacy Expectancy Scale) HRQoL ^||^ (MOS SF-36 ^†††††^ )	The IG ^‡^ had significantly higher adjusted mean self-efficacy scores (p < 0.05), adjusted mean levels of physical, general, mental and vitality functioning (p < 0.05), significantly higher exercise adherence (p < 0.01) and lower reported stress (p < 0.01) at three months post-surgery compared to the CG ^†^ . There was a significant improvement in pain and emotional functioning scores over time (p < 0.05)

*HF = Heart failure; ^†^CG = Control Group; ^‡^IG = Intervention Group; ^§^NYHA = New York Heart Association; ^||^HRQoL = Health-related quality of life; ^¶^SCA = Acute coronary syndrome; **HR = Heart Rage; ^††^VO_2_ = Oxygen volume; ^‡‡^EQ-5D = EuroQol-5 dimension-5 levels; ^§§^SMS = Text messages; ^||||^SF - Short form health survey; ^¶¶^BP = Blood pressure; ***WHO-5 = Well-Being Index from the World Health Organization-5 itens; ^†††^KCCQ = Kansas City Cardiomyopathy Questionnaire; ^‡‡‡^CAD = Coronary artery disease; ^§§§^EQ-VAS = EuroQol Visual Analogue Scale; ^||||||^MLHFQ = Minnesota Living With Heart Failure Questionnaire; ^¶¶¶^TC6M = 6-Minute Walk Test; ****HeartQoL = Health-related quality of life questionnaire; ^††††^ECG = Electrocardiogram; ^‡‡‡‡^AF = Atrial fibrillation; ^§§§§^PHQ-9 = Patient Health Questionnaire-9 items; ^||||||||^AMI = Acute myocardial infarction; ^¶¶¶¶^HFSS = Heart Failure Symptom Scale; *****HADS = Hospital Anxiety and Depression Scale; ^†††††^MOS SF-36 = Medical Outcomes Short-Form Health Survey-36 items

This systematic review reveals that the scientific literature on the subject is constantly expanding, especially with the inclusion of studies of high methodological quality. The first articles were published in 2003^(73-74)^, and since then there has been no reduction in the number of publications. On the contrary, a significant increase in studies was observed, especially after the advent of the pandemic, when the results related to the use of telemedicine gained even more relevance.

The articles that met the inclusion criteria were subjected to a critical evaluation of their methodological quality (n=44). The results ranged from 53.85% to 92.31% of congruence with the instrument used^(29)^. It is important to note that the main inconsistencies were found in the blinding of the outcome evaluators, the intervention applicators and the patients. Full details of the evaluations can be found in the Supplementary Material (https://doi.org/10.48331/scielodata.MI2JBD).

Regarding the characteristics of the population of the included studies, most patients were followed up with HF (68.18%, n = 30)^(31,34,37,38,41,44-48,50-54,56,59-60,62,64-74)^, acute coronary syndrome (ACS) (15.22%, n = 7)^(32-33,36,40,58,61,63)^ or coronary artery disease (CAD) (10.64%, n = 5)^(35,40,42,54,57)^. In four studies^(40,43,54,71)^, the intervention was aimed at a mixed population of patients with different heart diseases.

With regard to the resources and interventions adopted, the telemedicine modalities varied considerably, with emphasis on hybrid interventions that employ multiple tools to maximize treatment efficacy. Telemonitoring was the most frequently used intervention, appearing in 31 studies (68.89%)^(31-32,34-35,38-46,48-49,51-53,56-60,62-64,67,68,70,73-74)^. In addition to telemonitoring, another intervention that stood out as a widely used support resource was the telephone call present in 23 articles (51.11%)^(34,36,37,39,41-42,45,47,51-52,57,60-62,65-73)^. In most cases, they were used as complementary support^(34,39,41-42,45,47,51-52,57,60-62,67-68,70,73)^, assisting in communication and patient monitoring. In some situations, however, they constituted the main form of intervention^(36-37,65-66,69,71-72)^, highlighting their importance in scenarios where other technologies may not be available or feasible.

In addition to these interventions, telerehabilitation was identified in 13 studies (28.88%)^(32,35,39,41-42,47,50,52,54-58)^. Among the hybrid approaches, the combination of telemonitoring and telerehabilitation stands out, present in nine studies (20%)^(32,35,39,41-42,52,56-58)^. This approach offers real-time monitoring of vital signs and patient progress, in addition to remote exercise prescription. In turn, the use of short message services (SMS) alone is less common, cited in only one article^(33)^, but frequently combined with other interventions^(36,43,47,54-55,62)^ due to its ability to send reminders, instructions and ongoing motivations.

Of the 44 studies analyzed, the primary or secondary outcomes included a pre- and post-intervention comparison of HRQoL between the intervention and control groups. In this sense, a diversity was observed among the instruments used, with 81.81% (n=36) of the studies opting to use a single questionnaire^(31-33,36-42,44-49,51-56-58,60-66,68-72,74)^, while 11.36% (n=5) used two questionnaires^(50,55,59,67,73)^ and 6.81% (n=3) used three questionnaires^(34-35,43)^. Among the scales used, the Short Form Health Survey-36 (SF-36) was the most common, being adopted in 17 (38.63%) of the clinical trials^(34,39,41-44,52,55,59-61,63-64,67,70-74)^, while 12 (26.66%) studies adopted the Minnesota Living With Heart Failure Questionnaire (MLHFQ) scale^(38,45-48,50,59,62,67-69,73)^. Variations of the EuroQol questionnaire were used in ten studies (24.44%)^(32,35-36,43,49-50,55,58,71-72)^.

The GRADE assessment^(30)^ revealed a serious risk of bias, the heterogeneity in the reports of measurement of effects prevented the performance of a pooled analysis. Figure 3 presents the evidence profile, separating the studies by questionnaires used and showing that the certainty of the evidence varied from low to very low.

Regarding the HRQoL results, none of the selected studies showed a worsening effect on the HRQoL of the telemedicine-based IG throughout the follow-up. Furthermore, 45.45% (n = 20) of the studies had a positive, statistically significant relationship with the HRQoL scores in the intervention group^(33-34,41,45,47-49,51,54-55-58-60-65-68-70)^. However, the comprehensive analysis of the studies revealed that 11.36% (n = 5) did not observe any impact on HRQoL throughout the follow-up^(36,38,40,66-67)^. In contrast, 43.18% (n = 19) demonstrated positive improvements in HRQoL, without reaching a statistically significant difference between the groups^(31-32,35,37,39,42-44,46,50,52-53,56,57,59,71,74)^. Table 1 provides a detailed summary of the HRQoL results of the evaluated studies.

**Figure 3 -:** GRADE evidence profile

**Certainty Assessment**							**Number of Patients**		**Effect**		**Garin, et al**	**Importance**
**Number of Studies**	**Study Design**	**Risk of Bias**	**Inconsistency**	**Indirect Evidence**	**Imprecision**	**Other Considerations**	**Telemedicine**	**Usual Care**	Relative (95% CI*)	Absolut (95% CI*)		
Health-related quality of life (follow-up: average 8.9 months; assessed with: EuroQol (EQ-5D ^†^ | EQ-5D-5L ^‡^ | EQ-5D-Y ^§^ ); Scale for: 0 to 100)												
7	Randomized clinical trials	Very critical ^||^	Not critical	Not critical	Not critical	None	649	613	-	**0** (0 to 0)	⨁⨁◯◯ Low	CRITICAL
Health-related quality of life (follow-up: average 7.8 months; assessed with: MLHFQ ^¶^ | HFSS**; Scale from: 0 to 105)												
13	Randomized clinical trials	Very critical ^††^	Not critical	Not critical	critical ^‡‡^	None	1309	1102	-	**0** (0 to 0)	⨁ ◯◯◯ Very Low	CRITICAL
Health-related quality of life (follow-up: mean 8.8 months; assessed with: SF ^§§^ -36 | SF ^§§^ -12 | SF ^§§^ -8; Scale from: 0 to 100)												
16	Randomized clinical trials	Very critical ^||||^	Not critical	Not critical	Not critical	None	2850	2800	-	**0** (0 to 0)	⨁ ⨁ ◯◯ Low	CRITICAL
Health-related quality of life (follow-up: average 10.5 months; assessed with: KCCQ ^¶¶^ ; Scale of: 0 to 100)												
4	Randomized clinical trials	Very critical***	Not critical	Not critical	Not critical	None	1497	1528	-	**0** (0 to 0)	⨁ ⨁ ◯◯ Low	CRITICAL
Health-related quality of life (follow-up: average 9 months; assessed with: HeartQol ^†††^ ; Scale of: 0 to 3)												
2	Randomized clinical trials	Very critical ^‡‡‡^	Not critical	Not critical	Not critical	None	127	126	-	**0** (0 to 0)	⨁ ⨁ ◯◯ Low	CRITICAL
Health-related quality of life (follow-up: average 3 months; assessed with: MacNew; Scale from: 1 to 7)												
2	Randomized clinical trials	Very critical ^‡‡‡^	Not critical	Not critical	Not critical	None	67	64	-	**0** (0 to 0)	⨁ ⨁ ◯◯ Low	CRITICAL

*CI = Confidence interval; ^†^EQ-5D = EuroQol-5 Dimension; ^‡^EQ-5D-5L= EuroQol-5 dimension-5 levels; ^§^EQ-5D-Y = EuroQol-5 dimension-young; ^||^Not critical = 7 studies with some concerns regarding randomization bias (allocation concealment, blinding of professionals who performed the interventions) and 5 studies with bias related to blinding of outcome assessors; ^¶^MLHFQ = Minnesota Living with Heart Failure Questionnaire; **HFSS = Heart Failure Symptom Scale; ^††^Very critical = 12 studies with some concerns regarding randomization bias (allocation concealment, blinding of professionals who performed the interventions and of outcome assessors); ^‡‡^Critical= 7 studies with assay at high risk of imprecision due to lack of outcome data; ^§§^SF = Short form health survey; ^||||^Very critical = 15 studies with some concerns regarding randomization bias (allocation concealment, blinding of professionals who performed the interventions) and 11 studies with bias related to blinding of outcome assessors; ^¶¶^KCCQ= Kansas City Cardiomyopathy Questionnaire; ***Very critical = 4 studies with some concerns regarding randomization bias (allocation concealment, blinding of professionals who performed the interventions) and 3 studies with bias related to blinding of outcome assessors; ^†††^HeartQol = *Health-related Quality of life questionnaire*; ^‡‡‡^Very critical = 2 studies with some concerns regarding randomization bias (allocation concealment, blinding of professionals who performed the interventions)

**Table 1 -:** Summary of HRQoL results (n = 44). São Paulo, Brazil, 2024

**Study**	**Follow-up (months)**	**Instrument**	**Group**	**n**	Initial Score Mean (SD*)	Final Score Average (SD*)	**p-value**	Change Mean (SD*)	**Difference between groups (p-value)**
Choi, et al. ^( 31 )^	3	MacNew	CG ^†^	38	5.45 (0.86)	5.34 (1.09)	-	-	0.771
			IG ^‡^	38	5.62 (0.82)	5.62 (0.78)	-	-	
Dalli Peydró, et al. ^( 32 )^	10	EQ-5D-5L ^§^	CG ^†^	28	70 (65-85)	80 (70-90)	0.008	-	>0.800
			IG ^‡^	31	75 (60-90)	88 (68-90)	0.064	-	
Chow, et al. ^( 33 )^	12	SF ^||^ -12	CG ^†^	708	-	PS ^¶^ : 47.6(-)MCS**: 51.2 (-)	-	-	PS ^¶^ : 0.045
			IG ^‡^	716	-	PS ^¶^ : 47.6 (9.5) MCS**: 48.9 (9.6)	-	-	
Voller, et al. ^( 34 )^	12	KCCQ ^††^	CG ^†^	319	59.3 (24.0)	-	-	-	<0.01
			IG ^‡^	302	59.6 (23.0)	-	-	-	
Brouwers, et al. ^( 35 )^	12	EQ-5D-5L ^§^	CG ^†^	147	0.815 (0.010)	0.848 (0.016)	-	-	0.002
			IG ^‡^	153	0.814 (0.011)	0.851 (0.015)	-	-	
Maddison, et al. ^( 36 )^	13	EQ-5D ^‡‡^	CG ^†^	153	64 (22)	-	-	-	-
			IG ^‡^	153	62 (22)	-	-	-	-
Collins, et al. ^( 37 )^	3	KCCQ ^††^	CG ^†^	245	-	-	-	-	0.25
			IG ^‡^	246	-	-	-	-	
Clays, et al. ^( 38 )^	6	MLHFQ ^§§^	CG ^†^	22	30.0 (13.5)	-	0.58	1.7 (13.8)	0.5
			IG ^‡^	34	32.1 (22.9)	-	0.7	-1.0 (14.4)	
Batalik, et al. ^( 39 )^	3	SF ^||^ -36	CG ^†^	28	50.9 (8.8)	61.5 (7.1)	0.01	-	0.56
			IG ^‡^	28	53.1 (6.7)	62 (7)	0.01	-	
Lunde, et al. ^( 40 )^	12	HeartQoL ^||||^	CG ^†^	147	2.48 (0.54)	2.57 (0.51)	-	-	<0.05
			IG ^‡^	153	2.43 (0.59)	2.64 (0.51)	-	-	
		EQ-5D ^‡‡^	CG ^†^	147	72 (14)	75 (12)	-	-	<0.001
			IG ^‡^	153	69 (18)	78 (16)	-	-	
Piotrowicz, et al. ^( 41 )^	14-26	SF ^||^ -36	CG ^†^	425	88.8 (14.1)	88.9 (14.4)	-	-	0.008
			IG ^‡^	425	89.7 (12.6)	91.2 (12.8)	-	-	
Ávila, et al. ^( 42 )^	3	SF ^||^ -36	CG ^†1^	30	73.3 (15.1)	76.4 (16.4)	-	-	0.06
			CG ^†2^	30	79.8 (16.1)	82.6 (15.8)	-	-	
			IG ^‡^	30	82.2 (13.3)	82.6 (13)	-	-	
Cáceres, et al. ^( 43 )^	6	SF ^||^ -36	CG ^†^	123	PS ^¶^ : 47.6 (9.5) MCS**: 48.9 (9.6)	PS ^¶^ : 50.1 (8.5) MCS**: 52.7 (7.5)	-	-	PS ^¶^ : 0.37 MCS**: 0.74
			IG ^‡^	115	PS ^¶^ : 49.6 (8.6) MCS**: 50.8 (8.5)	PS ^¶^ : 52.5 (9.1) MCS**: 53.1 (8.6)	-	-	
		AFEQT ^¶¶^	CG ^†^	123	64.8 (25.2)	80.2 (20.9)	-	-	0.09
			IG ^‡^	115	66.3 (21)	79 (20.3)	-	-	
		EQ-5D ^‡‡^	CG ^†^	123	0.85 (0.21)	0.91 (0.13)	-	-	0.98
			IG ^‡^	115	0.88 (0.16)	0.94 (0.14)	-	-	
Cichosz, et al. ^( 44 )^	12	SF ^||^ -36	CG ^†^	154	PS ^¶^ : 40.3 (9.2) MCS**: 48.9 (11.4)	PS ^¶^ : 40.67 (10.2) MCS**: 46.65 (12.1)	-	-	NS***
			IG ^‡^	145	PS ^¶^ : 40.3 (9) MCS** 47.4 (10)	PS ^¶^ : 40.58 (9.7) MCS**: 50.01 (11.5)	-	-	< 0.05
		KCCQ ^††^	CG ^†^	154	62.5 (20.4)	63.56 (21.2)	-	-	<0.01
			IG ^‡^	145	64.7 (18.8)	66.31 (20.7)	-	-	
Mizukawa, et al. ^( 45 )^	24	MLHFQ ^§§^	CG ^†^	19	32.2 (27.8)	-	-	-	0.564
			IG ^‡1^	20	37.3 (22.7)	-	-	-	
			IG ^‡2^	20	47.5 (26.8)	-	-	-	
Pekmezaris, et al. ^( 46 )^	3	MLHFQ ^§§^	CG ^†^	58	59.9	27.8	-	-	0.5
			IG ^‡^	46	62.7	36.3	-	-	
Peng, et al. ^( 47 )^	4	MLHFQ ^§§^	CG ^†^	49	48.77 (12.21)	49.63 (12.39)	-	-	0.072
			IG ^‡^	49	49.43 (12.25)	42.32 (8.83)	-	-	
Wagenaar, et al. ^( 48 )^	12	MLHFQ ^§§^	CG ^†^	150	23.0 (32.5)	26.5	-	-	0.003
			IG ^‡1^	150	24.0 (31.0)	28.3	-	-	
			IG ^‡2^	150	23.0 (27.8)	25.5	-	-	
Guo, et al. ^( 49 )^	3	EQ-5D-Y ^†††^	CG ^†^	96	71.3	69.9	-	-	<0.05
			IG ^‡^	113	86.5	87.2	-	-	
Hwang, et al. ^( 50 )^	6	MLHFQ ^§§^	CG ^†^	29	47 (19)	33 (21)	-	-	-
			IG ^‡^	24	41 (22)	34 (23)	-	-	
Jayaram, et al. ^( 51 )^	6	KCCQ ^††^	CG ^†^	765	58.6 (24.9)	68.5 (26.4)	0.11	-	0.01
			IG ^‡^	756	60.7 (24.1)	72.3 (24.4)	0.01	-	
Piotrowicz, et al. ^( 52 )^	2	SF ^||^ -36	CG ^†^	34	73.6 (25.6)	67.4 (25.9)	NS**	-	0.0001
			IG ^‡^	77	79.0 (31.3)	70.8 (30.3)	0.001	-	
Bekelman, et al. ^( 53 )^	12	KCCQ ^††^	CG ^†^	193	36.9 (14.6)	54.2	-	-	0.97
			IG ^‡^	199	37.9 (13.3)	53.6	-	-	
Frederix, et al. ^( 54 )^	6	HeartQoL ^||||^	CG ^†^	70	2.31 (0.59)	2.32 (0.58)	0.21	-	0.01
			IG ^‡^	70	2.27 (0.63)	2.53 (0.44)	0.01	-	
Maddison, et al. ^( 55 )^	6	SF ^||^ -36	CG ^†^	86	PS ^¶^ : 51.9 (5.8) MCS**: 51.53 (8.4)	PS ^¶^ : 51.9(-) MCS**: 54(-)	-	-	PS ^¶^ : 0.2 MCS**: 0.61
			IG ^‡^	85	PS ^¶^ : 51.6 (5.9) MCS**: 52.87 (6.94)	PS ^¶^ : 52.9(-) MCS**: 54.6(-)	-	-	
		EQ-5D ^‡‡^	CG ^†^	86	0.8 (0.1)	0.83(-)	-	-	0.23
			IG ^‡^	85	0.8 (0.1)	0.86(-)	-	-	
Piotrowicz, et al. ^( 56 )^	2	SF ^||^ -36	CG ^†^	75	81.6 (27.3)	62.2 (26.4)	-	-	NS***
			IG ^‡^	77	79.3 (25.6)	70.5 (25.4)	-	-	
Kraal, et al. ^( 57 )^	3	MacNew	CG ^†^	26	5.2 (0.8)	5.8 (0.7)	-	-	0.498
			IG ^‡^	29	5.7 (0.7)	6.1 (0.5)	-	-	
Varnfield, et al. ^( 58 )^	1.5	EQ-5D ^‡‡^	CG ^†^	60	0.83 (0.8–0.9)	0.82 (0.7-0.9)	0.7	-	0.01
			IG ^‡^	60	0.84 (0.8–0.9)	0.92 (0.9–1.0)	<0.001	-	
Blum, et al. ^( 59 )^	12	MLHFQ ^§§^	CG ^†^	102	37 (27)	18 (21)	-	-	NS***
			IG ^‡^	104	42 (23)	24 (24)	-	-	
		SF ^||^ -36	CG ^†^	102	PS ^¶^ : 35 (11) MCS**: 49 (13)	PS ^¶^ : 38 (11) MCS**: 55 (9)	-	-	-
			IG ^‡^	104	PS ^¶^ : 37 (90) MCS**: 49 (12)	PS ^¶^ : 38 (10) MCS**: 52 (11)	-	-	
Cui, et al. ^( 60 )^	12	SF ^||^ -36	CG ^†^	55	44.61 (23.3)	49.05 (19.59)	-	-	0.5341
			IG ^‡1^	61	45.93 (19.47)	55.63 (27.51)	-	-	
			IG ^‡2^	58	44.73 (17.79)	40.17 (23.38)	-	-	
Hawkes, et al. ^( 61 )^	6	SF ^||^ -36	CG ^†^	215	46.7 (9.6)	46.7 (11.1)	-	0.3	0.73
			IG ^‡^	215	45.8 (10.1)	46.4 (11.5)	-	-	
Seto, et al. ^( 62 )^	6	MLHFQ ^§§^	CG ^†^	50	47.8 (22.6)	47.3 (23.4)	0.9	-	0.05
			IG ^‡^	50	50.3 (29.1)	41.4 (26.7)	0.02	-	
Blasco, et al. ^( 63 )^	12	SF ^||^ -36	CG ^†^	101	-	-	-	-	NS***
			IG ^‡^	102	-	-	-	-	
Koehler, et al. ^( 64 )^	12	SF ^||^ -36	CG ^†^	356	-	51.7 (1.4)	-	0.3	<0.05
			IG ^‡^	354	-	53.8 (1.4)	-	-	
Baker, et al. ^( 65 )^	1	HFSS ^‡‡‡^	CG ^†^	303	64.8 (22.4)	64.1 (22.8)	-	-0.6	<0.001
			IG ^‡^	302	58.6 (22.2)	65.3 (22.4)	-	6.7	
Copeland, et al. ^( 66 )^	12	SF ^||^ -8	CG ^†^	238	-	-	-	-	NS***
			IG ^‡^	220	-	-	-	-	
Balk, et al. ^( 67 )^	9.6	MLHFQ ^§§^	CG ^†^	113	-	-	-	-	NS***
			IG ^‡^	101	-	-	-	-	
Schwarz, et al. ^( 68 )^	3	MLHFQ ^§§^	CG ^†^	51	35.8 (21.5)	27.3 (21.6)	-	-	NS***
			IG ^‡^	51	39.5 (23.3)	27.4 (21.7)	-	-	
Wakefield, et al. ^( 69 )^	12	MLHFQ ^§§^	CG ^†^	49	60.6 (19.3)	56.6 (23.9)	-	-	0.0002
			IG ^‡1^	52	58.4 (22.9)	41.5 (26.9)	-	-	
			IG ^‡2^	47	60.2 (24.8)	54.0 (26.0)	-	-	
Woodend, et al. ^( 70 )^	12	SF ^||^ -36	CG ^†^	125	-	-	-	-	-
			IG ^‡^	124	-	-	-	-	
López Cabezas, et al. ^( 71 )^	12	EQ-5D ^‡‡^	CG ^†^	64	65 (17.6)	60.6 (17.8)	-	-	NS***
			IG ^‡^	70	62.3 (17.3)	64 (15.4)	-	-	
Riegel, et al. ^( 72 )^	6	EQ-5D ^‡‡^	CG ^†^	65	57.1 (16.7)	73.7 (17.4)	-	-	-
			IG ^‡^	69	60.4 (19.9)	73.4 (17.4)	-	-	-
		MLHFQ ^§§^	CG ^†^	65	56.1 (16.7)	12.9 (10.9)	-	-	
			IG ^‡^	69	52.7 (19.6)	12.1 (12.3)	-	-	
Benatar, et al. ^( 73 )^	3	MLHFQ ^§§^	CG ^†^	108	77.17 (8.52)	57.72 (16.24)	0.47	-	<0.01
			IG ^‡^	108	77.92 (10.3)	51.64 (17.36)	0.98	-	
Barnason, et al. ^( 74 )^	3	SF ^||^ -36	CG ^†^	17	60.7	69.1	-	-	-
			IG ^‡^	18	60.7	78.3	-	-	

*SD = Standard Deviation; ^†^CG = Control group; ^‡^IG = Intervention group; ^§^EQ-5D-5L = EuroQol-5 dimension-5 levels; ^||^SF = Short form health survey; ^¶^PS = Summary of the physical component of 36-Item Short Form Survey; **MCS = Summary of the mental component of 36-Item Short Form Survey; ^††^KCCQ = Kansas City Cardiomyopathy Questionnaire; ^‡‡^EQ-5D = EuroQol-5 dimension; ^§§^MLHFQ = Minnesota Living With Heart Failure Questionnaire; ^||||^HeartQoL = Health-related quality of life questionnaire; ^¶¶^AFEQT = Atrial Fibrillation Effect on Quality-of-life; ***NS = Not significant; ^†††^EQ-5D-Y= EuroQol-5 dimension young; ^‡‡‡^HFSS =Heart Failure Symptom Scale

## Discussion

Telemedicine interventions focused on self-management demonstrated a positive relationship in improving the HRQoL of patients with heart disease in 45.45% of the included studies^(33-34,41,45,47-49,51,54-55-58-60-65-68-70)^. These interventions were mostly applied to individuals with HF^(31,34,37-38,41,44-48,50-54,56,59-60,62,64-74)^, ACS^(32-33,36,40,58,61,63)^ and CAD^(35,40,42,54,57)^. Among the main resources used were telemonitoring, telephone calls, telerehabilitation, SMS and video consultations^(31-74)^.

The scientific literature on telemedicine has shown steady growth since the first studies were published in 2003, with a significant increase in research during the COVID-19 pandemic^(11)^. This systematic review analyzed 44 randomized clinical trials, involving a total of 12,732 patients. Despite a slight numerical disparity between the control (6,233) and intervention (6,499) groups, the distribution was considered balanced by the authors of the included studies. The studies were conducted mainly in Europe (40.91%) and North America (36.36%), with significant participation also from Oceania (13.64%) and Asia (9.09%). This geographic distribution highlights the broad acceptance and application of telemedicine technologies in diverse cultural and infrastructural contexts.

However, the expansion of telemedicine during the pandemic has also highlighted weaknesses, inequalities, and limitations in health systems that may have previously been less noticeable^(11)^. Despite the positive geographic dissemination across continents, geographic gaps have been identified, especially in regions such as Latin America and Africa. The shortage can be attributed to the limited technological infrastructure and lower investments in telemedicine research in these locations^(11)^. This highlights the need for multifaceted approaches to address these challenges and ensure a more comprehensive representation of telemedicine research on a global scale.

The measurement and assessment of HRQoL are challenges frequently faced in scientific research due to the multidimensional nature and the diverse definitions associated with this concept^(75)^. Thus, considering this complexity, a variety of instruments have been developed and have been used to assess HRQoL^(75)^. Overall, in the present review, the use of 17 different instruments was observed, with the predominance of a single questionnaire^(31-33,36-42,44-49,51-56-58,60-66,68-72,74)^ for the assessment of the HRQoL outcome.

In this sense, the choice between using generic and condition-specific instruments to measure this variable has distinct advantages. Generic instruments allow the comparison of HRQoL between different health conditions, offering a broad and comparative view in different clinical situations. On the other hand, condition-specific measures focus directly on the assessment of HRQoL related to the condition under study, making them clinically more relevant instruments for understanding the specific impact of the disease on patients’ lives^(76)^.

In this way, the use of dual perspectives provides more complete and complex interpretations in the approach to HRQoL in health research, allowing a more precise and informative analysis of the challenges faced by patients in different life contexts^(76)^. Therefore, in this review, only nine studies performed HRQoL analysis based on two or three questionnaires^(34-35,43,50,55,59,67,73-74)^.

In the population studied, a predominance of patients with HF was observed, representing 68.18% of the total^(31,34,37-38,41,44-48,50-54,56,59-60,62,64-74)^. HRQoL in these patients was assessed using 10 questionnaires, including six specific to this population: MLHFQ^(38,45-48,50,59,62,67-69,73)^, KCCQ^(34,51,53,37)^, MacNew^(31)^, HeartQol^(54)^, HFSS^(65)^, 70-item Quality of Life Index – Cardiac^(73)^, and four generic questionnaires: SF-36^(34,41,44,52,56,59-60,64,66,67,70,74)^, SF-8^(66)^, WHO-5^(34)^, and ED-5Q^(50,71-72)^. Considering the context of HF, the use of the MLHFQ instrument is considered positive, as shown in a previous systematic review, which indicated it as the most suitable scale to measure HRQoL in these individuals, with the KCCQ as a secondary option^(77)^.

Regarding the impact on patients’ HRQoL, a significant portion of the included studies (45.45%) showed positive results, with statistical significance, in the intervention group^(33-34,41,45,47-49,51,54-55-58-60-65-68-70)^. Approximately 11.36% of the RCTs indicated no impact whatsoever^(36,38,40,66-67)^, while another 43.18% indicated improvements in HRQoL, although without reaching a statistically significant difference^(31-32,35,37,39,42-44,46,50,52-53,56,57,59,71,74)^. This diversity of results highlights the complexity and multifaceted nature of HRQoL as a health outcome, highlighting the importance of considering contextual and intervention-specific factors when assessing its impact on patients’ lives^(75-76)^. Furthermore, although telemedicine is promising, its effectiveness varies due to methodological differences, characteristics of the populations included, and types of intervention. This finding is in line with a meta-analysis on the effectiveness of telemedicine in the management of NCDs, which indicated an improvement in HRQoL in studies on cardiovascular diseases, although without statistical significance^(78)^.

In this sense, another important point to be highlighted is that the results of telemedicine interventions can be compared to traditional in-person care, without demonstrating inferiority. None of the included studies identified deterioration in HRQoL among participants. This suggests that the implementation of telemedicine in the care of cardiovascular conditions does not have adverse consequences on patients’ HRQoL and may even generate benefits for other health outcomes, such as repercussions on physical activity^(32,34,39-42,47,52,54,57,61)^, use of health resources (hospitalizations, days of hospitalization, emergency room visits)^(45,69,71,73)^, repercussions on mental health^(38,46,53,63,73)^ and self-care^(31,38,48,60,62)^.

Regarding the interventions used in the studies, it was observed that telemonitoring was the most comprehensive resource. When used in isolation, it presented positive repercussions on HRQoL in only 36.36% (4 of 11) of the studies^(48-49,63-64)^. Similarly, RCTs that addressed only telephone calls demonstrated positive repercussions on HRQoL in only 33.33% (1 of 3)^(61)^. However, combined interventions, such as the association of telemonitoring with phone calls, video consultations or SMS, showed statistically significant differences in 7 of 11 studies (63.63%)^(34,45,51,60,62,68,70)^. In the case of telerehabilitation, which was always associated with another listed resource, a positive repercussion on HRQoL was observed in 38.46% (5 of 13) of the RCTs^(41,47,54-55,58)^. These mixed results were also reflected in a systematic review of 19 studies on telemonitoring in heart failure, which identified heterogeneity in the studies that measured HRQoL, as well as questionable methodological quality and sample limitations^(79)^. Regarding telerehabilitation, there was an improvement in HRQoL in eight studies, but without superiority in relation to usual care^(32,35,39,42,50,52,56-57)^, which is corroborated by a systematic review comparing telerehabilitation to center-based rehabilitation, which demonstrated that this intervention is as effective in improving HRQoL^(23)^.

In this sense, considering that cardiac telerehabilitation seems acceptable to patients and comparable to usual care, it may be an effective way to increase the reach and adherence to rehabilitation^(80)^. However, future studies are needed to explore how telerehabilitation can be effectively integrated into health systems and how health professionals can be trained and supported to provide this type of care appropriately.

Regarding the follow-up period of the studies, a significant variation was observed between the RCTs, with periods ranging from one to 26 months, which may contribute to the heterogeneity of the results. Considering the positive and statistically significant impact on HRQoL, 10 studies performed follow-up for up to six months^(47,49,51,54-55,58,61-62,65,68)^. Eight RCTs followed patients for 12 months^(33-34,48,60,63-64,69-70)^, while one study performed follow-up for 24 months^(45)^ and another for 26 months^(41)^.

In a previous systematic review, it was observed that the improvement in HRQoL appears to be more evident in the short term in patients with CAD and in patients with HF over three months^(81)^. This suggests the need for further studies on the relationship between follow-up time, population, and telemedicine resources to help identify the best care for each patient, considering that the literature is still incipient in this regard.

Thus, the use of more robust and improved methodologies, in addition to the standardization of telemedicine intervention protocols for heart disease, should be encouraged, since this will contribute to a more solid understanding of the real benefits and obstacles of the approach in the clinical context of these conditions.

Regarding the limitations of the selected studies, a predominance of sampling issues was observed, evidenced in the critical analysis of the studies. Only one study reported blinding of participants^(63)^, two studies mentioned the blinding of professionals who performed the interventions^(33,47)^, and 12 studies indicated that the outcome evaluators were blinded to the allocation of participants^(32-33,36,40-41,47,50,52-55,64)^. In addition, other limitations were cited, such as sample size^(31-32,37-40,43-45,58,60,62,67,69,74)^, follow-up time^(31,35,41,44,49,56)^ and selection or recruitment bias^(34-35,41,46-47,50-54,66-68,73)^ often due to the predominance of a specific cardiovascular disease, the majority presence of the male population or even the place of recruitment.

As implications for practice, we have that the use of telemedicine resources can be considered essential support tools to provide comprehensive care to the individual in intra- and extra-hospital environments, mainly considering its benefits for various health outcomes. However, robust studies are still needed to better measure the effect of the telemedicine strategy and its influence on health outcomes, such as follow-up time, patient and professional satisfaction, and equity of access, given that the interactions between outcomes are complex and must consider different contexts and populations.

Regarding the assessment of HRQoL, it is important to emphasize the need for studies with multifaceted approaches, considering the complexity of measuring this variable, due to its multidimensional nature, in order to ensure more accurate interpretations^(75-76)^. In addition, the importance of deepening the relationships between follow-up time, population, and telemedicine intervention can also be mentioned.

Likewise, effective public policies and interventions adapted to the needs of each community^(9)^, based on standardized protocols, are extremely important, since they have the potential to expand access to health, overcoming geographical barriers in promoting care^(15)^.

Among the limitations of this review, we highlight the non-inclusion of gray literature, which could have expanded the search for complementary evidence. Furthermore, the review faced challenges related to the heterogeneity of interventions and resources used, as well as the specificity of the cardiac diseases addressed and the outcomes assessed. These variations made it difficult to conduct a meta-analysis, preventing a robust quantitative synthesis. However, the qualitative results remain valid to assess the effectiveness of the interventions in the contexts studied.

## Conclusion

The effectiveness of telemedicine on HRQoL in individuals with CDs is still inconclusive. Although most studies have demonstrated a positive impact, many have not reached statistical significance. The main interventions used in the care and self-management of these conditions include telemonitoring, telephone contact and telerehabilitation. Telemedicine has the potential to be a valuable tool, comparable to face-to-face interventions in health centers. However, more studies are needed to evaluate its safety, cost-effectiveness and other long-term outcomes, especially HRQoL monitoring, to optimize the implementation of these technologies and ensure better outcomes for patients.
