# Supplementary material for: Effect of telemedicine on the quality of life of people with heart disease: a systematic review
Source: Rev Lat Am Enfermagem. 2025 Jul 11;33:e4566. doi: 10.1590/1518-8345.7243.4566 (PMC12259226; doi:10.1590/1518-8345.7243.4566)
Supplement: Translation

# Translation: Efeito da telemedicina na qualidade de vida de pessoas com doenças cardíacas: uma revisão sistemática

**DOI:** 10.1590/1518-8345.7243.4567

**Authors:** Gabriele Cardoso Gonçalves Alves, Fabiola Leticia Damascena Amador, Vagner Rogério dos Santos, Rita Simone Lopes Moreira

**Affiliations:** 1 Universidade Federal de São Paulo, Escola Paulista de Enfermagem, São Paulo, SP, Brasil.; 2 Universidade Federal de São Paulo, Escola Paulista de Medicina, São Paulo, SP, Brasil.

**Keywords:** Telemedicina, Cardiopatias, Autogestão, Qualidade de Vida, Telerreabilitação, Autocuidado

## Abstract

sintetizar as evidências científicas sobre a efetividade das intervenções baseadas em telemedicina na melhoria da qualidade de vida relacionada à saúde e ao autogerenciamento de pacientes com doenças cardíacas.

revisão sistemática de efetividade, seguindo as recomendações *do Joanna Briggs Institute* e do guia de relato *PreferredReportingItems for Systematic Reviews and Meta-Analyses Checklist*. A busca foi conduzida em seis bases de dados: *Cochrane Library*, Biblioteca Virtual em Saúde, PubMed, CINAHL, *Web of Science Core Collection* e Scopus, sem restrição de período, nos idiomas inglês, português ou espanhol. A qualidade metodológica e o risco de viés dos estudos foram avaliados utilizando a ferramenta de avaliação crítica da JBI e a certeza da evidência foi classificada por meio da ferramenta GRADE.

dos 44 ensaios clínicos randomizados incluídos, as principais intervenções analisadas foram telemonitoramento, contato telefônico e telerreabilitação. Dos estudos avaliados, 88,63% demonstraram melhora na qualidade de vida relacionada à saúde, com 45,45% destes apresentando diferença estatisticamente significativa.

a telemedicina mostra-se promissora como ferramenta valiosa para o cuidado e o autogerenciamento de indivíduos com condições cardíacas. No entanto, são necessários mais estudos para confirmar sua efetividade e seu impacto nos desfechos de saúde.

## Destaques

(1) Principais recursos observados foram: telemonitoramento, ligação e telerreabilitação. (2) A telemedicina pode ser uma ferramenta efetiva no cuidado de condições cardíacas. (3) O impacto real na qualidade de vida em relação às doenças cardíacas ainda é incerto. (4) Estudos relacionados aos aspectos da qualidade de vida ainda são necessários.

## Introdução

As doenças crônicas não transmissíveis (DCNT) representam uma parcela significativa das causas de morte no mundo, correspondendo a cerca de 74% dos óbitos no âmbito global^(1)^. Dentre elas, as doenças cardiovasculares (DCVs) assumem uma posição alarmante, sendo responsáveis por aproximadamente dois milhões de óbitos na região das Américas no ano de 2019^(2)^.

Diante desse cenário, enfrentar o desafio das DCVs torna-se uma prioridade, especialmente devido ao contínuo envelhecimento da população mundial e à complexidade multifatorial que caracteriza essas doenças^(3)^. Além disso, as DCNTs, com destaque para as doenças cardíacas, estão tornando-se um marcador crescente das desigualdades globais, pois são altamente incidentes nos países em desenvolvimento^(4)^.

As doenças cardíacas são condições patológicas envolvendo o coração, no que tange à sua estrutura e função^(5)^. O desenvolvimento, a progressão e a piora dessas condições estão diretamente relacionados a fatores de risco, que podem ser classificados como potencialmente modificáveis, como o controle da pressão arterial, glicemia e perfil lipídico; ou modificáveis, como a cessação do tabagismo, redução do consumo excessivo de álcool, combate à obesidade, sedentarismo, entre outros^(6)^. Assim, o uso de iniciativas educativas que busquem fomentar o letramento em saúde é recomendável, visto que essas ações possuem o potencial de agir em tais fatores de risco atenuando eventos adversos e readmissões hospitalares, ao mesmo tempo que podem incrementar a qualidade de vida relacionada à saúde (QVRS) dos indivíduos afetados^(7)^.

Nesse contexto, é observado que populações com elevado risco de DCV apresentam resultados de QVRS mais desfavoráveis^(7)^. Dessa forma, essa variável emerge como um indicador crucial, sendo um forte preditor tanto para mortalidade quanto para hospitalização por insuficiência cardíaca (IC), independentemente da gravidade dos sintomas ou da fração de ejeção^(8)^.

Assim, com o intuito de enfrentar essa realidade, a telemedicina emerge como uma estratégia promissora para diminuir a mortalidade em indivíduos com problemas cardíacos e promover a QVRS e o autocuidado^(9)^. Ademais, conforme mostrado em uma revisão sistemática sobre a eficácia, a aceitabilidade e os custos da telemedicina, realizada em 2015, ao considerar estratégias para melhoria de fatores de risco, houve melhora de QVRS, diminuição de níveis de hemoglobina glicada, lipoproteínas de baixa densidade e pressão arterial em indivíduos com DCNT^(10)^.

É incontestável que durante a pandemia de coronavírus houve ampla adoção desse formato de cuidado à distância como uma maneira alternativa às consultas presenciais, tal fato ocorreu em resposta às restrições ao contato direto, evidenciando a capacidade de adaptação do sistema diante de desafios emergentes^(11)^.

A telemedicina é definida pela prestação de serviços de saúde de forma remota^(12)^, ou seja, o paciente e o prestador estão separados pela distância, mediados por uma ferramenta tecnológica^(13)^. Existem diversos recursos nessa modalidade de cuidado em saúde, como consultas com profissionais de saúde (teleconsulta, linhas diretas e linhas de apoio), telemonitoramento, telerreabilitação, armazenamento e encaminhamento de dados de saúde (como imagens, notas e vídeos) aos prestadores de cuidado e teleconsultoria entre profissionais de saúde que prestam o cuidado, comunicando-se em busca de outras opiniões para o gerenciamento de casos^(13-14)^.

Diante disso, a telemedicina apresenta papel essencial na assistência à saúde global, sendo capaz de estruturar iniciativas terapêuticas que englobam objetivos educacionais, diagnósticos precisos e monitoramento contínuo^(15-16)^. Ainda, ao superar limitações geográficas^(15)^, apresenta potencial para aumentar o acesso aos cuidados, reduzir os custos e melhorar os resultados gerais de saúde^(16)^. Dessa forma, existe a preocupação por parte dos profissionais de saúde em relação à habilidade dos indivíduos de autogerenciar suas próprias condições nesse formato de cuidado^(9)^.

Logo, o autogerenciamento é a “habilidade de gerenciar sintomas, tratamento, mudanças físicas e psicossociais, bem como mudanças de estilo de vida que os pacientes desenvolvem ao lidar com doenças crônicas”^(17)^, geralmente necessitando do suporte de um profissional de saúde^(18)^. Nesse contexto, acredita-se que a telemedicina tenha um impacto positivo no suporte ao autogerenciamento, consequentemente melhorando a QVRS de pacientes com doenças cardíacas.

Até o momento, revisões sistemáticas prévias que abordaram doenças cardíacas e telemedicina não se concentraram especificamente no autogerenciamento e nas mudanças da QVRS ao longo do tempo, abordando outros desfechos como mortalidade, internação e outros^(19-23)^. Ademais, os estudos relacionados à QVRS percebida pelos pacientes têm mostrado divergências nos resultados^(19-20,23-24)^ e conforme indicado em uma revisão sistemática, são necessários estudos utilizando medidas padronizadas para avaliação de QVRS, autocuidado e satisfação^(10)^.

Nesse contexto, esta revisão teve como objetivo sintetizar as evidências científicas sobre a efetividade das intervenções baseadas em telemedicina na melhoria da QVRS e no autogerenciamento de pacientes com doenças cardíacas.

## Método

Trata-se de uma revisão sistemática, conduzida segundo as recomendações do *Joanna Briggs Institute* (JBI): *Evidence Synthesis Groups* ^(25)^ e reportada de acordo com as recomendações do *Preferred Reporting Items for Systematic Reviews and Meta-Analyses* PRISMA *Checklist* ^(26)^. O protocolo foi previamente publicado na *Open Science Framework* ^(27)^, em 22 de janeiro de 2021, sob o DOI 10.17605/OSF.IO/HQWGT. A busca foi realizada em março de 2023.

A pesquisa foi norteada no acrônimo PICOS (P – *Population;* I – *Intervention*; C – *Comparison*; O - *Outcomes*; S -Study)^(25)^, sendo o P (indivíduos com idade maior ou igual a 18 anos e com doenças cardíacas), o I (recursos de telemedicina que suportem o autogerenciamento), C (cuidados usuais, baseados em consultas ambulatoriais), o O (QVRS) e S (ensaios clínicos controlados e randomizados). Com base nesse acrônimo, elaborou-se a seguinte pergunta norteadora: Qual é a efetividade das intervenções de telemedicina que são baseadas no autogerenciamento em comparação aos cuidados usuais na QVRS de pacientes adultos com doenças cardíacas?

## Critérios de elegibilidade

Foram incluídos ensaios clínicos randomizados (ECRs) que compararam cuidados usuais com o uso da telemedicina em pacientes adultos (≥ 18 anos) com doenças cardíacas, avaliando os resultados na QVRS e no autogerenciamento desses indivíduos. Os critérios de exclusão foram: ECRs que não detalharam a metodologia utilizada e artigos que incluíam outras condições clínicas (por exemplo, câncer e diabetes).

## Fontes de dados

Para a seleção dos artigos, a estratégia de busca foi implementada pelo pesquisador principal nas seguintes bases de dados: *Cochrane Library* (*Wiley*), Biblioteca Virtual em Saúde (Portal BVS), *Medical Literature Analysis and Retrieval System Online* (MEDLINE, PubMed), CINAHL (*EBSCOhost*), *Web of Science Core Collection* e *Scopus* (via Portal Periódicos CAPES).

## Estratégia de busca

A estratégia de busca foi específica para cada base de dados a partir do uso de dois vocabulários controlados em saúde: *Medical Subject Headings* (MeSH) e Descritores em Ciências da Saúde (DeCS). Os descritores utilizados foram: “*Telemedicine”*, *”Heart Diseases”*, *”Self-Management”*, *”Cardiac Rehabilitation”* e”*Health Education”,* adequados para cada base de dados. As estratégias de busca completas podem ser consultadas no Material Suplementar (https://doi.org/10.48331/scielodata.MI2JBD).

## Seleção e extração de dados

Dois revisores independentes selecionaram os estudos, e um terceiro avaliador resolveu as divergências. Primeiramente, os títulos e resumos relacionados à questão de pesquisa e ao objetivo do estudo foram identificados, e aqueles potencialmente elegíveis foram pré-selecionados. Na segunda etapa, dois revisores independentes avaliaram os textos completos dos estudos pré-selecionados para confirmar sua elegibilidade. O processo de seleção foi realizado por meio da plataforma *Rayyan QCRI* ^(28)^.

Os dados foram extraídos dos estudos que foram anteriormente incluídos na revisão por dois revisores independentes, que utilizaram um formulário de extração de dados pré-determinados. O formulário incluiu os seguintes eixos: detalhes metodológicos, intervenção e resultados [o documento pode ser consultado no Material Suplementar (https://doi.org/10.48331/scielodata.MI2JBD)]. Devido à complexidade das intervenções, os estudos selecionados foram categorizados segundo a população estudada e a intervenção realizada, considerando os recursos utilizados. Salienta-se ainda que os dados extraídos incluíram detalhes específicos sobre os participantes, métodos do estudo, intervenções e resultados significativos para o objetivo da revisão. Quaisquer discordâncias surgidas entre os revisores foram resolvidas por meio de discussão ou com um terceiro revisor. Não foi necessário contatar os autores dos artigos para dados adicionais.

## Síntese dos dados

Os achados foram apresentados na forma narrativa. A síntese narrativa incluiu uma descrição detalhada dos estudos incluídos, categorizados segundo a população estudada, questionários utilizados para mensuração da QVRS, tempo de seguimento e intervenções de telemedicina realizadas. Ademais, tabelas e figuras foram usadas para auxiliar na apresentação dos dados.

## Avaliação crítica dos estudos

Dois revisores independentes realizaram uma avaliação crítica de todos os estudos que atenderam aos critérios de inclusão. Qualquer discrepância entre os revisores foi resolvida por consenso ou pela intervenção de um terceiro revisor. A qualidade metodológica dos estudos foi verificada utilizando o *checklist* de avaliação crítica do JBI para ensaios clínicos randomizados. Este *checklist* é composto por 13 questões cujas respostas podem ser “sim”, “não”, “incerto” ou “não aplicável”^(29)^. Todos os estudos foram classificados em cinco domínios diferentes, resultando em classificações de baixo risco de viés, risco de viés moderado ou alto risco de viés. A certeza da evidência para o desfecho QVRS foi avaliada usando a ferramenta *Grading of Recommendations Assessment, Development and Evaluation* (GRADE)^(30)^.

## Resultados

A busca na literatura inicial identificou o total de 2.341 estudos provenientes de bases de dados, além de quatro estudos adicionais encontrados por meio de busca manual em listas de referências de artigos incluídos. Destes, dois estudos foram incluídos na busca primária para desenvolvimento da estratégia de pesquisa, enquanto os outros dois foram identificados após a análise dos protocolos de estudos encontrados na busca final.

Após a remoção de duplicatas e a revisão dos títulos e resumos, 80 estudos foram selecionados para leitura integral. As razões para exclusão nesta etapa foram relacionadas principalmente aos critérios como população, intervenção, comparação ou desfecho. Os detalhes sobre os motivos de exclusão podem ser encontrados no Material Suplementar (https://doi.org/10.48331/scielodata.MI2JBD).

A partir da leitura integral e análise da qualidade metodológica dos estudos, considerando os critérios de elegibilidade, foi constituída a amostra final que contou com 44 ensaios clínicos randomizados. A Figura 1 apresenta o fluxograma detalhado do processo de seleção dos estudos incluídos nesta revisão sistemática.

**Figura 1 -:** Fluxograma PRISMA^(26)^

A síntese descritiva dos ECRs incluídos nesta revisão está apresentada na Figura 2 abaixo.

Os 44 ECRs incluídos nesta revisão envolveram o total de 12.732 pacientes, dos quais 6.233 foram alocados no grupo controle e 6.499 no grupo intervenção. Além do mais, as pesquisas foram realizadas em quatro continentes, com 40,91% (n=18) dos estudos conduzidos na Europa^(32,34-35,38-42,44,48,52,54,56-57,63-64,67,71)^, 36,36% (n=16) na América do Norte^(37,43-46,51-53,59-60,62,65-67,63-64,66,68-70,72-74)^, 13,64% (n=6) na Oceania^(33,36,50,55,58,61)^ e 9,09% (n=4) na Ásia^(31,45,47,49)^. O período de seguimento variou de um a 26 meses, com uma média de acompanhamento de 8,4 meses.

**Figura 2 -:** Descrição das características dos estudos incluídos na revisão sistemática (n = 44)

Autor, ano, país	População, n, seguimento	Intervenção	Recursos	Comparação	Principais desfechos	Resultados principais
Choi, et al. ^( 31 )^ 2023 Coreia	Indivíduos com diagnóstico de IC* n:76 (GC ^†^ :38| GI ^‡^ : 38) Seguimento: 3 meses	Programa de autogerenciamento via aplicativo móvel (“ *Heart Failure-Smart Life* ”)	Aplicativo “ *Heart Failure-Smart Life* ” Conteúdo educativo Bate-papo individual com profissional de saúde Registros diários de saúde para acompanhamento personalizado e interativo, com recursos adaptados às necessidades individuais *Feedback* de autogerenciamento por enfermeira Aplicativo distinto para os profissionais de saúde	Cuidados habituais: consultas com cardiologista e enfermeira cardiológica; breves informações sobre os medicamentos e a evolução da doença.	Medidas antropométricas Classificação funcional NYHA ^§^ Depressão ( *Geriatric* *Depression* *scale* ) QVRS ^||^ ( *MacNew Heart Disease Health-Related QoL* ) Adesão medicamentosa ( *Hill-Bone Medication Adherence* ) Autocuidado ( *European Heart Failure Self-Care Behaviour Scale* )	No comparativo entre grupos houve melhorias significativas na classe funcional NYHA ^§^ (p = 0,003) no GI ^‡^ . A QVRS ^||^ não mostrou diferenças significativas entre os grupos (GC ^†^ =5,34; GI ^‡^ =5,62). O comportamento de autocuidado melhorou ao longo do tempo (p < 0,001), mas sem diferenças significativas entre os grupos
Dalli Peydró, et al. ^( 32 )^ 2022 Espanha	Indivíduos pós-alta hospitalar por SCA ^¶^ n: 59 (GC ^†^ : 28 | GI ^‡^ : 31) Seguimento: 10 meses	Telerreabilitação, precedida por treinamento hospitalar por 2 semanas, após ajustes de ritmo e metas, o aplicativo guiou os participantes através de um programa diário de exercícios	Aplicativo: Programa diário de atividades físicas Registro do estado geral Sinais vitais Adesão medicamentosa Monitor de FC**	Reabilitação cardíaca baseada em centro	Aumento da atividade física referida (Questionário internacional de atividade física) VO _2_^††^ máx. QVRS ^||^ (EQ-5D ^‡‡^ *5-levels* )	O GI ^‡^ apresentou maior aumento na atividade física (1.726 vs. 636 múltiplos de equivalentes metabólicos –mín./semana, p = 0,045) e VO _2_^††^ máx. (1,62 vs. 0,60 ml/kg min), p < 0,004). A adesão à dieta mediterrânea, o sofrimento psicológico e a QVRS ^||^ mostraram maior melhora no GI ^‡^ do que no GC ^†^ . A autoavaliação da saúde melhorou em ambos os grupos, mas só foi significativa no GI ^‡^ (p = 0,008).
Chow, et al. ^( 33 )^ 2022 Austrália	Indivíduos com diagnóstico SCA ^¶^ n:1424 (GC ^†^ :708| GI ^‡^ :716) Seguimento: 12 meses	Programa de SMS ^§§^ personalizadas destinado a melhorar a adesão a medicamentos e a prevenção secundária	Envio de SMS ^§§^ motivacionais e educacionais divididas em três módulos principais: estilo de vida (dieta, exercício e cessação do fumar), medicamentos (informações sobre uso e efeitos colaterais), prevenção secundária geral (metas de saúde, apoio mental e serviços de saúde) Conselheiro de saúde	Os cuidados habituais não foram descritos	Adesão medicamentosa autorreferida Medidas antropométricas e laboratoriais QVRS ^||^ (SF ^||||^ -12)	Não houve diferença significativa na adesão medicamentosa autorrelatada entre os GI ^‡^ e GC ^†^ 0,93 [intervalo de confiança 95%, 0,84–1,03]; (P=0,15). Não houve diferença nas pontuações de depressão e ansiedade, mas houve uma leve melhora na pontuação de QVRS ^§^ (componente físico) para o GI ^‡^ (diferença média [intervalo de confiança 95%], 1,1 [0,0, 2,2]; P=0,045)
Voller, et al. ^( 34 )^ 2022 Alemanha	Indivíduos com diagnóstico de IC* n:621 (GC ^†^ :319 |GI ^‡^ :302) Seguimento: 12 meses	Telemonitoramento domiciliar via sistema Motiva ^®^ , que capacita o paciente a gerenciar sua condição de saúde e facilita o contato diário entre médicos e pacientes	Sistema Motiva ^®^ Registro regular de parâmetros vitais (PA ^¶¶^ , FC** e peso) Material educativo, material de treinamento, questionários, lembretes e *feedback* sobre o estado de saúde via Motiva ^®^ Acompanhamento telefônico de possíveis descompensações	Cuidado usual, baseado nas diretrizes da Sociedade Europeia de Cardiologia	Custo-efetividade incremental Mortalidade QVRS ^||^ (SF ^||||^ -36 V2; WHO-5***; KCCQ ^†††^ ) Capacidade funcional	A intervenção não teve impacto no risco de mortalidade. Todas as escalas de QVRS ^||^ melhoraram de forma consistente e significativa no GI ^‡^ , aos 12 meses, em comparação com o GC ^†^ (todos p < 0,01)
Brouwers, et al. ^( 35 )^ 2021 Holanda	Indivíduos em reabilitação (fase 2) para DAC ^‡‡‡^ n: 300 GC ^†^ : 147 | GI ^‡^ : 153) Seguimento: 12 meses	Telerreabilitação, cujo programa incluiu 6 sessões de exercícios em grupo, consultas semanais por vídeo realizadas por fisioterapeutas até a conclusão das metas individuais ou do programa, além do telemonitoramento semanal	Aplicativo: Monitoramento de sinais vitais Monitor de FC** Acelerômetro Módulo de treinamento físico Vídeo consultas semanais	Reabilitação cardíaca baseada em centro	QVRS ^||^ (EQ-5D ^‡‡^ *5-levels* ; EQ-VAS ^§§§^ ; *MacNew Heart Disease Health-Related Quality of Life Questionnaire* ) Custos em saúde	Os pacientes do GI ^‡^ e do GC ^†^ apresentaram QVRS ^||^ comparáveis (diferença média no EQ-5D-5L ^‡‡^ : −0,004; p = 0,82; diferença média no EQ-VAS ^§§§^ : −0,001). Embora os custos de intervenção tenham sido mais altos, não houve diferenças nos custos totais de saúde cardíaca entre os dois grupos (€4.787 vs. €5.507, p = 0,36)
Maddison, et al. ^( 36 )^ 2021 Nova Zelândia	Indivíduos adultos com SCA ^¶^ n:306 (GC ^†^ :153| GI ^‡^ :153) Seguimento: 13 meses	Programa de SMS ^§§^ automatizadas denominado *Text4Heart II* , promovendo o autogerenciamento e a educação em saúde	Envio de SMS ^§§^ personalizadas Ligações telefônicas para aplicação dos questionários	Cuidados habituais ambulatoriais, sem detalhamento	Adesão medicamentosa ( *Morisky Medication Adherence Scale* ) QVRS ^||^ (EQ-5D ^‡‡^ )	A adesão à medicação em três classes foi menor no GI ^‡^ em comparação com o GC ^†^ , tanto em 24 semanas (56,8% vs. 68,6%) quanto em 52 semanas (67,9% vs. 54,2%). Os escores de autorrelato de adesão à medicação refletiram essa tendência em 52 semanas
Collins, et al. ^( 37 )^ 2021 Estados Unidos da América	Indivíduos com diagnóstico de IC* n:491 (GC ^†^ :245 | GI ^‡^ :246) Seguimento: 12 meses	Cuidados usuais e plano de alta personalizado, *coaching* telefônico de autocuidado, com intervenção composta por visita domiciliar dentro de 7 dias após a alta e treinamento de autocuidado por telefone	Ligações telefônicas Consulta por telessaúde	Cuidados usuais compostos por processo de alta estruturado, incluindo reconciliação e prescrição medicamentosa; e consulta médica para acompanhamento	Morte associada à doença cardiovascular Eventos relacionados à IC* QVRS ^||^ (KCCQ ^†††^ )	Não houve uma diferença significativa entre os grupos em relação à mortalidade, eventos relacionados à IC* e mudanças na pontuação resumida do KCCQ ^†††^ aos 90 dias. Embora a pontuação resumida do KCCQ ^†††^ tenha sido maior no GI ^‡^ , a diferença não foi estatisticamente significativa (intervalo de confiança 95% -1,9 a 7,2; P = 0,25)
Clays, et al. ^( 38 )^ 2021 Bélgica e Itália	Indivíduos com diagnóstico de IC* n:56 (GC ^†^ :22 | GI ^‡^ :34) Seguimento: 6 meses	Sistema de telemonitoramento domiciliar que consistia em uma combinação de dispositivos de monitoramento e um aplicativo móvel, destinados a fornecer suporte abrangente para o gerenciamento da saúde dos pacientes	Sistema *HeartMan* : Monitor vestível de FC** e PA ^¶¶^ Balança Organizador de comprimidos Smartphone com o aplicativo móvel *HeartMan* , com recursos para gerenciamento físico, suporte psicológico e educação sobre IC* Serviço de assistência telefônica operacional	Cuidados habituais com o tratamento padrão de acordo com as diretrizes clínicas oferecidas pelo cardiologista, clínico geral e enfermeira da IC*	QVRS ^||^ (MLHFQ ^||||||^ ) Capacidade de exercício (TC6M ^¶¶¶^ ) Percepção de doença e saúde mental ( *Brief Illness Perception. Questionnaire* ) Autocuidado ( *Self-Care of Heart Failure Index* )	No GI ^‡^ , houve redução significativa da depressão e ansiedade (p < 0,001), enquanto no GC ^†^ a necessidade de aconselhamento sexual diminuiu (p < 0,05). Apenas no GI ^‡^ , o autocuidado aumentou (p < 0,05) e os problemas sexuais diminuíram (p < 0,05), porém não houve impacto significativo na QVRS ^||^ confiança no autocuidado, percepção da doença ou capacidade de exercício entre os grupos
Batalik, et al. ^( 39 )^ 2020 República Tcheca	Indivíduos com doença cardiovascular, submetidos à revascularização cardíaca n: 56 (GC ^†^ : 28 | GI ^‡^ : 28) Seguimento: 3 meses	Telerreabilitação, precedida por treinamento supervisionado em ambulatório	Aplicativo: Monitoramento de sinais vitais Monitor de FC** Módulo de treinamento físico Reavaliação telefônica semanal	Reabilitação cardíaca baseada em centro	Aptidão física (Teste Cardiopulmonar de Exercício) QVRS ^||^ (SF ^||||^ -36) Adesão ao treinamento	Ambos os grupos mostraram melhorias significativas na aptidão física (P < 0,001), com adesão semelhante entre eles. A QVRS ^||^ melhorou significativamente em ambos os grupos, sem diferença significativa entre eles (P < 0,01)
Lunde, et al. ^( 40 )^ 2020 Noruega	Indivíduos em reabilitação (fase 2) para DAC ^‡‡‡^ n: 300 GC ^†^ : 147 |GI ^‡^ :153) Seguimento: 12 meses	Acompanhamento individualizado via aplicativo, com definição de metas, lembretes, avaliação de progresso e *feedback* personalizado	Aplicativo: Orientações comportamentais (atividade física e dieta) Hábitos saudáveis Definição de metas individuais Lembretes *Feedbacks* individuais	Os cuidados usuais não foram detalhados	Diferença no VO _2_^††^ pico Desempenho no exercício Peso corporal PA ^¶¶^ em repouso Hábitos de exercício QVRS ^||^ ( *HeartQoL* ****) Estado de saúde (EQ-5D ^‡‡^ *5-levels* )	No GI ^‡^ , houve diferença significativa no VO _2_^††^ pico em relação ao GC ^†^ (diferença média de 2,2 ml/kg/min, intervalo de confiança 95% 0,9–3,5, p = 0,001). Além disso, o GI ^‡^ apresentou melhor desempenho no exercício, hábitos de exercício e autopercepção do cumprimento de metas, em comparação com o GC ^†^ . Não foram encontradas diferenças significativas entre os grupos em pressão arterial, QVRS ^||^ e estado de saúde
Piotrowicz, et al. ^( 41 )^ 2020 Polônia	Indivíduos com diagnóstico de IC* n:850 (GC ^†^ :425|GI ^‡^ :425) Seguimento: 14 a 26 meses	Telerreabilitação iniciada com treinamento hospitalar, seguida de treinamento domiciliar. Incluiu supervisão médica, educação e planejamento de exercícios personalizados para cada paciente	Central de monitoramento, com disponibilização de telefone celular para: Registro de sinais vitais e dados clínicos Treinamento físico individualizado Programa educacional ECG ^††††^ portátil Monitor de PA ^¶¶^ Balança Monitor de cardiodesfibrilador (apenas para pacientes com o implante)	Os cuidados usuais incluíram avaliações clínicas regulares, participação em programas de reabilitação, monitoramento remoto de dispositivos cardíacos implantáveis, aconselhamento sobre mudanças de estilo de vida e autogerenciamento de acordo com as diretrizes clínicas padrão	Porcentagem de sobrevida fora do hospital Mortalidade Mudança na duração do teste de exercício cardiopulmonar QVRS ^||^ (SF ^||||^ -36)	Não houve diferenças significativas na taxa de mortalidade (12,5% vs. 12,4%, respectivamente; taxa de risco, 1,03 [Intervalo de confiança 95%, 0,70-1,51]) ou hospitalização (média [DP] de dias: 91,9 [19,3] vs. 92,8 [18,3], respectivamente; P = 0,74) entre os grupos. No entanto, o GI ^‡^ apresentou benefícios significativos em termos de melhoria do consumo máximo de oxigênio e QVRS ^||^ (pontuação do SF ^||||^ -36, 1,58 [Intervalo de confiança 95%, 0,74-2,42] vs. 0,00 [Intervalo de confiança 95%, -0,84 a 0,84]; P = 0,008), sendo bem tolerada sem eventos adversos graves durante o exercício
Ávila, et al. ^( 42 )^ 2020 Bélgica	Indivíduos com DAC ^‡‡‡^ n:90 (GC ^†^ cuidados usuais:30| GC ^†^ reabilitação:30 | GI ^‡^ : 30) Seguimento: 3 meses	Programa de telerreabilitação associado ao telemonitoramento e à prescrição individualizada de exercício	Aplicativo Vestível (relógio) *Feedback* por telefone ou e-mail	Reabilitação baseada em centro com treinamento ambulatorial. Cuidados usuais: incentivados a manter um estilo de vida fisicamente ativo e foram convidados para consultas de acompanhamento	Aptidão cardiorrespiratória (VO _2_^††^ pico). Atividade física Fatores de risco cardiovasculares tradicionais QVRS ^||^ (SF ^||||^ -36)	Todos os grupos mantiveram pontuações elevadas para todos os parâmetros de QVRS ^||^ , sem diferenças significativas entre os grupos (p = 0,70). A capacidade de exercício e os resultados secundários foram preservados nos três grupos ao longo do tempo (p > 0,05 para todos), independentemente da intervenção (p > 0,05 para todos)
Cáceres, et al. ^( 43 )^ 2020 Estados Unidos da América	Indivíduos com diagnóstico de FA ^‡‡‡‡^ ou *Flutter* Atrial n:238 (GC ^†^ :123|GI ^‡^ :115) Seguimento: 6 meses	Intervenção *iHEART* de telemonitoramento e orientações via SMS ^§§^	Uso de *iPhone* com sistema de ECG ^††††^ móvel *AliveCor* ^®^ *Kardia* para monitoramento cardíaco remoto Envio de SMS ^§§^ com orientações sobre manejo da FA ^‡‡‡‡^ e estilo de vida	Cuidados habituais com o tratamento-padrão de acordo com as diretrizes vigentes	Recorrência de FA ^‡‡‡‡^ QVRS ^||^ (SF ^||||^ -36; EQ-5D-5L ^‡‡^ ; *Atrial Fibrillation Effect on Quality of Life* ) Gravidade dos sintomas de FA ^‡‡‡‡^ ( *Atrial Fibrilation Severity Scale* )	Melhora em ambos os grupos nas pontuações desde o início até o acompanhamento para as pontuações da escala de efeito da FA ^‡‡‡‡^ na QVRS ^||^ (p < 0,05), com maior repercussão no GI ^‡^ . Não houve diferenças estatisticamente significativas na QVRS ^||^ ou na gravidade dos sintomas de FA ^‡‡‡‡^ entre os grupos
Cichosz, et al. ^( 44 )^ 2019 Dinamarca	Indivíduos com diagnóstico de IC* n:299 (GC ^†^ :154|GI ^‡^ :145) Seguimento: 12 meses	Intervenção *Telekits* , com sistema clínico central que contava com monitoramento de sinais vitais, avaliação de enfermagem, contatos telefônicos para manejo e encaminhamento para consultas médicas, caso necessário	*Tablets* Monitores de PA ^¶¶^ digitais Balanças Contato telefônico	Reabilitação, aconselhamento dietético, treinamento, monitoramento de medicamentos, triagem de fatores de risco e conversas sobre mudanças no estilo de vida	QVRS ^||^ (SF ^||||^ -36): escore físico e mental Escore do questionário específico de IC* (KCCQ ^†††^ )	O GI ^‡^ apresentou melhora significativa no escore mental comparado ao GC ^†^ (p < 0.01). Não houve efeito significativo na melhoria do escore físico (SF ^||||^ -36) ou na qualidade de vida relacionada à IC* (KCCQ ^†††^ )
Mizukawa, et al. ^( 45 )^ 2019 Japão	Indivíduos com diagnóstico de IC* n: 59 (GC ^†^ :19| GI ^‡1^ :20| GI ^‡ 2^ : 20) GI ^‡1^ : Gestão colaborativa GI ^‡2^ : Educação para autogestão Seguimento: 24 meses	Sistema de monitoramento e autogerenciamento, com registro diário de sinais vitais, visitas ao médico, sessões mensais de educação em autogestão e monitoramento remoto telefônico por enfermeira para o GI ^‡1^	Monitor de FC** Monitor de PA ^¶¶^ Monitoramento de peso Intervenção educativa Gerenciamento telefônico via enfermeira, apenas para o GI ^‡1^	Visitas ao médico a cada 2-4 semanas, uma sessão de educação na alta e orientação para registro diário de peso, PA ^¶¶^ e pulso em um caderno de autogerenciamento	QVRS ^||^ (MLHFQ ^||||||^ ) Autoeficácia ( *Chronic Disease Self-Efficacy Scale* ) Autocuidado ( *European Heart Failure Self- care Behaviour ScaleS* ) Reinternação por IC* Mortalidade por todas as causas	A QVRS ^||^ melhorou significativamente no grupo de GI ^‡1^ comparado aos GC ^†^ , aos 18 e 24 meses (P < 0.05). O grupo GI ^‡1^ também apresentou melhorias significativas em autoeficácia e autocuidado (P < 0.01), e teve menores taxas de nova hospitalização (20.0% vs. 57.9% no GC ^†^ ), com maior sobrevivência sem readmissão (P = 0.020)
Pekmezaris, et al. ^( 46 )^ 2019 Estados Unidos da América	Indivíduos negros e hispânicos de comunidades carentes com diagnóstico primário de IC* n: 104 (GC ^†^ :58 | GI ^‡^ :46) Seguimento: 3 meses	Automonitoramento via telessaúde, focada na autogestão diária e visitas semanais de telessaúde, com suporte intensivo e contínuo por parte de um enfermeiro	Monitor de FC** Monitor de PA ^¶¶^ Monitoramento de peso Monitor de saturação de oxigênio Vídeoconsulta semanal com enfermeira	Gerenciamento ambulatorial abrangente, baseado nos cuidados habituais em ambiente ambulatorial, com adesão às diretrizes da *American Heart Association*	Hospitalização e utilização de serviço de emergência QVRS ^||^ (MLHFQ ^||||||^ ) Ansiedade e depressão ( *Patient Health Questionnaire -4* )	Os resultados indicaram que não houve diferença significativa entre o GI ^‡^ e o GC ^†^ nas visitas ao pronto-socorro (Risco Relativo = 1,37, intervalo de confiança = 0,83–2,27), hospitalizações (Risco Relativo = 0,92, intervalo de confiança = 0,57–1,48) ou tempo de internação (GI ^‡^ = 0,54 vs. GC ^†^ = 0,91). Ambos os grupos tiveram um incremento nos valores de QVRS ^||^ , sem diferenças estatísticas
Peng, et al. ^( 47 )^ 2018 China	Indivíduos com diagnóstico de IC* e seus cuidadores n:98 (GC ^†^ :49 | GI ^‡^ :49) Seguimento: 4 meses	Telerreabilitação, programa de treinamento físico por telessaúde com sessões de exercícios monitoradas por fisioterapeutas e acompanhamento regular de enfermeiros cardíacos por meio de telefone ou SMS ^§§^	Programa de treinamento físico por telessaúde Folheto educativo Sessões de exercícios, com supervisão por vídeo Acompanhamento com enfermeiras via ligações telefônicas ou mensagens instantâneas ( *Wechat* )	Cuidados usuais, baseados em sessão educacional na alta hospitalar e visitas regulares de acompanhamento na clínica. Os pacientes deste grupo não receberam instruções específicas sobre exercícios físicos.	QVRS ^||^ (MLHFQ ^||||||^ ) Distância percorrida no TC6M ^¶¶¶^ Medidas fisiológicas Ansiedade e depressão ( *Hospital Anxiety and Depression Scale* )	O GI ^‡^ demonstrou melhorias estatisticamente significativas na QVRS ^||^ (Fb = 8,272, P = 0,005), com uma sustentação de até 4 meses após o pós-teste (Fin = 79,73, P = 0,000) e na distância percorrida no TC6M ^¶¶¶^ em comparação com o GC ^†^ no pós-teste. Não foram observadas melhorias significativas em relação aos demais parâmetros
Wagenaar, et al. ^( 48 )^ 2018 Holanda	Indivíduos com diagnóstico de IC* n:450 (GC ^†^ :150| GI ^‡^ site:150 | GI ^‡^ e-Vita:150) Seguimento: 12 meses	A intervenção foi composta por um *site* que continha conteúdos educativos em saúde. Além disso, os participantes do GI ^‡^ e-Vita seguiram um plano de cuidados em e-saúde com telemonitoramento e ajustes personalizados	*Site* Orientações de enfermagem Plataforma e-Vita Registro regular de parâmetros vitais (peso, PA ^¶¶^ e FC**) Alertas acionados em caso de valores fora dos limites preestabelecidos Atualizações sobre comorbidades e medicamentos Lembretes mensais por e-mail para acompanhamento e adesão ao plano de cuidados	Cuidados habituais de nove equipes ambulatoriais de IC*, incluindo consultas de rotina com um cardiologista e uma enfermeira de IC*, em média, quatro vezes por ano	Autocuidado do paciente ( *European Heart Failure Self-Care Behavior Scale* ) QVRS ^||^ (MLHFQ ^||||||^ ) Conhecimento específico da doença ( *Dutch Heart Failure knowledge Scale* ) Satisfação do paciente com os cuidados de IC* Mortalidade	Após 3 meses, os GI ^‡^ apresentaram melhor autocuidado em comparação ao GC ^†^ (GI ^‡^ site 73,5 vs. 70,8 e GI ^‡^ e-Vita 78,2 vs. 70,8, respectivamente). Além disso, após 3 e 6 meses, houve diferenças significativas na QVRS ^||^ entre o GI ^‡^ e-Vita e o GC ^†^ (mediana GI ^‡^ e-Vita 19,0 vs. GC ^†^ 22,8, p = 0,029 e GI ^‡^ e-Vita 21,0 vs. GC ^†^ 24,0, P = 0,003), respectivamente. Essas diferenças diminuíram ao longo do tempo, sem diferenças após 1 ano
Guo, et al. ^( 49 )^ 2017 China	Indivíduos com FA ^‡‡‡‡^ n: 209 (GC ^†^ :96 | GI ^‡^ :113) Seguimento: 3 meses	Sistema de autogerenciamento baseado em um aplicativo móvel, oferecendo suporte à decisão clínica, aos programas educacionais, ao monitoramento de saúde e ao acompanhamento estruturado	Aplicativo de suporte à decisão clínica: Cálculo automático de escores de risco Programas educacionais Componentes de envolvimento no autocuidado Componentes de seguimento estruturado Registro pessoal de saúde	Os cuidados usuais não foram descritos	Conhecimento dos pacientes sobre FA ^‡‡‡‡^ QVRS ^||^ (EQ-5D ^‡‡^ ) Adesão à medicação ( *Pharmacy Quality Alliance* ) Usabilidade, viabilidade e aceitabilidade do aplicativo	Mais de 90% dos pacientes relataram que o aplicativo era fácil de usar e útil, associado a melhorias significativas no conhecimento (p <0,05). Adesão aos medicamentos e satisfação com anticoagulantes foram significativamente melhores no GI ^‡^ em comparação com o GC ^†^ (p < 0,05). Os escores de QVRS ^||^ aumentaram significativamente com o aplicativo, com redução de ansiedade e depressão (p < 0,05)
Hwang, et al. ^( 50 )^ 2017 Austrália	Indivíduos com diagnóstico de IC* n:53 (GC ^†^ :29 | GI ^‡^ :24) Seguimento: 6 meses	Telerreabilitação, com programa realizado duas vezes por semana com vídeo consultas, supervisão de um fisioterapeuta, exercícios em tempo real, sessões educativas e suporte remoto	Telerreabilitação por meio de uma plataforma de videoconsultas Interação entre o fisioterapeuta e os participantes, *feedback* e modificações de planos terapêuticos *Slides* educacionais com arquivos de áudio incorporados Discussões em grupo *online* Desenho colaborativo Funções de bate-papo Manual de equipamentos com instruções escritas e pictóricas	Reabilitação tradicional baseada em centro	QVRS ^||^ (MLHFQ ^||||||^ ; ^‡‡^ EQ-5D) Distância percorrida no TC6M ^¶¶¶^ Satisfação ( *Client Satisfaction Questionnaire* ) Taxa de eventos adversos	As análises não revelaram diferenças significativas entre os grupos em relação à QVRS ^||^ , indicando uma similaridade nos resultados. Ambos os grupos apresentaram melhorias estatisticamente significativas em sua QVRS ^||^ , com manutenção durante o período de acompanhamento. Em relação à distância percorrida no TC6M ^¶¶¶^ , não foram observadas diferenças significativas entre os grupos, com uma diferença média de 15 metros (intervalo de confiança 95% –28 a 59).
Jayaram, et al. ^( 51 )^ 2017 Estados Unidos da América	Indivíduos com diagnóstico de IC* n:1521 (GC ^†^ :765 | GI ^‡^ :756) Seguimento: 6 meses	Programa Tele-HF de telemonitoramento baseado em ligações telefônicas	Materiais educativos Balança Ligações diárias com resposta a questionários *Feedback* médico, se necessário Lembretes	Os cuidados usuais não foram detalhados, citados como recomendações padrão das diretrizes para o tratamento da IC*, com materiais educativos e recebimento de uma balança, se necessário	QVRS ^||^ (KCCQ ^†††^ )	Durante o acompanhamento de 6 meses, pacientes do GI ^‡^ apresentaram uma média de pontuação resumida geral do KCCQ ^†††^ 2,5 pontos maiores (intervalo de confiança 95% = 0,38, 4,67; p = 0,02) do que aqueles submetidos aos cuidados habituais. Esta diferença foi impulsionada principalmente por melhorias nos sintomas (3,5 pontos; intervalo de confiança 95%= 1,18, 5,82; p = 0,003) e função social (3,1 pontos; intervalo de confiança 95% = 0,30, 6,00; p = 0,03)
Piotrowicz, et al. ^( 52 )^ 2020 Polônia	Indivíduos com diagnóstico de IC* n:111 (GC ^†^ :34| GI ^‡^ : 77) Seguimento: 2 meses	Programa de telerreabilitação associado ao telemonitoramento e à prescrição individualizada de exercício, baseadas em marcha nórdica	Equipamento de telerreabilitação e monitoramento remoto: Mini Dispositivo EHO Monitor de PA ^¶¶^ Balança ECG ^††††^ Celular Questionários Suporte telefônico (apoio psicológico e instruções adicionais)	Cuidados usuais, sem a prescrição formal de treinamento físico ou reabilitação supervisionada. Recomendações para mudanças no estilo de vida e autogestão conforme as diretrizes da Sociedade Europeia de Cardiologia	Capacidade funcional (VO _2_^††^ pico) QVRS ^||^ (SF ^||||^ -36) Avaliação ampliada da eficácia da reabilitação Segurança e adesão ao treinamento	No GI ^‡^ , observou-se melhoria significativa na capacidade funcional (p = 0,0001) com repercussões positivas na QVRS ^||^ , sem diferenças estatísticas. Todos os participantes completaram o programa de reabilitação, com adesão elevada ao treinamento domiciliar de marcha nórdica, sendo 94,7% dos pacientes considerados aderentes
Bekelman, et al. ^( 53 )^ 2015 Estados Unidos da América	Indivíduos com diagnóstico de IC* n:392 (GC ^†^ :193| GI ^‡^ :199) Seguimento: 12 meses	O Programa *Patient-Centered* *Disease Management* combinou cuidados colaborativos multidisciplinares, telemonitoramento e manejo conjunto de IC* e depressão	Cuidados colaborativos multidisciplinar Triagem e tratamento da depressão Telemonitoramento e apoio ao autocuidado do paciente: Monitor de PA ^¶¶^ e FC** Monitoramento de peso e sintomas autorrelatados Lembretes de medicamentos Material de educação sobre IC* e depressão Aconselhamento dietético	Seguimento com a equipe de saúde de cuidados habituais, informações na visita de inscrição que descreviam o autocuidado para IC* e uma balança, se necessário, os médicos foram notificados caso apresentassem sintomas depressivos	QVRS ^||^ (KCCQ ^†††^ ) Sintomas depressivos (PHQ-9 ^§§§§^ ) Hospitalizações Mortalidade	Não houve diferenças significativas nas características basais entre os grupos de intervenção e cuidado usual. Após 1 ano, ambos os grupos apresentaram melhora semelhante nas pontuações do KCCQ ^†††^ (mudança média de 13,5 pontos, p = 0,97). A intervenção foi associada a menos mortes em 1 ano (4,3% vs. 9,6%, p = 0,04)
Frederix, et al. ^( 54 )^ 2015 Bélgica	Indivíduos com DAC ^‡‡‡^ em reabilitação cardíaca ou IC* n: 140 (GC ^†^ : 70| GI ^‡^ :70) Seguimento: 6 meses	Telerreabilitação, composta por telemonitoramento e *tele-coaching* , adicionado ao programa convencional de reabilitação cardíaca de 12 semanas, que foi baseado em centro	Telemonitoramento da atividade física Acelerômetro *Tele-coaching* via *website* Orientações sobre cessação do tabagismo, dieta e atividade física *Feedbacks* por e-mail ou SMS ^§§^	Reabilitação tradicional baseada em centro	Capacidade aeróbica máxima (VO _2_^††^ pico) Atividade física diária Hemoglobina glicada, controle glicêmico e perfil lipídico QVRS ^||^ ( *HeartQoL* ****)	Os pacientes do GI ^‡^ apresentaram um aumento significativo no VO _2_^††^ pico médio de linha de base (média de 22,46) para 24 semanas (média de 24,46, P<0,01), enquanto no GC ^†^ não houve mudança significativa. Além disso, a atividade física autorrelatada e a pontuação global de QVRS ^||^ melhoraram mais no GI ^‡^ em comparação com o GC ^†^ às 24 semanas (P=0,01)
Maddison, et al. ^( 55 )^ 2015 Nova Zelândia	Indivíduos com doença cardíaca isquêmica n: 171 (GC ^†^ :86 | GI ^‡^ :85) Seguimento: 6 meses	Programa HEART, que consistiu em prescrição de exercícios e suporte comportamental via SMS ^§§^ e internet, visando aumentar a atividade física em pacientes	Prescrição de exercícios regulares Fornecimento de estratégias de mudança de comportamento Site com informações sobre a doença e possibilidade de automonitoramento do progresso Envio diário de SMS ^§§^	Os cuidados usuais não foram descritos	Capacidade aeróbica máxima (VO _2_^††^ pico) Atividade física autorreferida (Questionário internacional de atividade física) Autoeficácia e motivação para exercícios QVRS ^||^ (SF ^||||^ -36 versão 2; EQ-5D ^‡‡^ )	Houve um aumento significativo na atividade física de lazer autorreferida (p = 0,05) e caminhada (p = 0,02) no GI ^‡^ . Também houve melhorias significativas na autoeficácia para ser ativo e no domínio de saúde geral do SF ^||||^ -36 no GI ^‡^ , com uma diferença de 2,1, intervalo de confiança 95%: 0,1, 4,1; p = 0,03) em 24 semanas para QVRS ^||^
Piotrowicz, et al. ^( 56 )^ 2015 Polônia	Indivíduos com diagnóstico de IC* n: 152 (GC ^†^ :75 | GI ^‡^ :77) Seguimento: 2 meses	Telerreabilitação, baseada em caminhadas, utilizando um sistema de telemonitoramento e treinamento supervisionado	Telerreabilitação com sessões de treinamento pré-programadas para cada indivíduo Telemonitoramento Envio de ECG ^††††^ de repouso e resposta de um questionário de saúde pré-atividade física Apoio psicológico	Reabilitação tradicional baseada em centro	QVRS ^||^ (SF ^||||^ -36)	Melhora na QVRS ^||^ total em ambos os grupos, entretanto não foi significativa. O GI ^‡^ teve uma melhora principalmente nas categorias mentais e o GC ^†^ melhorou seu bem-estar físico geral.
Kraal, et al. ^( 57 )^ 2014 Holanda	Indivíduos com DAC ^‡‡‡^ que ingressaram na reabilitação cardíaca após internação por IAM ^||||||||^ , angina instável ou revascularização (Risco baixo a moderado) n: 55 (GC ^†^ :26 | GI ^‡^ :29) Seguimento: 3 meses	Telerreabilitação, que iniciava com sessões de treinamento monitorado, estabelecimento de metas e entrevistas motivacionais, com *feedback* telefônico do fisioterapeuta	Telerreabilitação Telemonitoramento com monitor vestível de FC** Aplicativo *Feedback* por telefone	Reabilitação tradicional baseada em centro	VO _2_^††^ pico QVRS ( *MacNew Heart Disease Health-Related QoL* ) Adesão ao treinamento	Melhora significativa do consumo de VO _2_^††^ pico (GC ^†^ :10% e GI ^‡^ :14% respectivamente) em ambos os grupos, sem diferença significativa entre os grupos. A QVRS ^||^ relacionada à saúde melhorou significativamente em ambos os grupos, sem quaisquer diferenças entre eles
Varnfield, et al. ^( 58 )^ 2014 Austrália	Indivíduos pós IAM ^||||||||^ n: 120 (GC ^†^ : 60| GI ^‡^ :60) Seguimento: 6 meses	Telerreabilitação via Plataforma de Avaliação de Cuidados para Reabilitação Cardíaca (CAP-CR), baseada na prescrição de exercícios e envio de conteúdo educacional	Plataforma CAP-CR: Monitoramento de saúde Monitoramento de exercícios SMS ^§§^ e arquivos de áudio com conteúdo motivacional e educacional Portal da *web* Consultas semanais com os mentores	Reabilitação tradicional baseada em centro	Adesão, aderência e conclusão do programa de reabilitação cardíaca Fatores de risco biomédicos (PA ^¶¶^ , FC**, peso, dentre outros) QVRS ^||^ (EQ-5D ^‡‡^ )	O GI ^‡^ teve taxas de adesão (80% vs. 62%) e conclusão (80% vs. 47%) significativamente mais altas do que o GC ^†^ (p<0,05). Ambos os grupos melhoraram significativamente no TC6M ^¶¶¶^ , mantido aos 6 meses. O GI ^‡^ também apresentou repercussões positivas na QVRS (EQ-5D ^‡‡^ : mediana de 0,84 para 0,92)
Blum, et al. ^( 59 )^ 2014 Estados Unidos da América	Indivíduos com diagnóstico de IC* n: 206 (GC ^†^ : 102|GI ^‡^ :104) Seguimento: 12 meses	Sistema de telemonitoramento de sinais vitais, com monitoramento de enfermagem e contatos com o paciente	Sistema *E-care* da *Philips Electronics* para monitoramento remoto dos dados; Balança Monitor de PA ^¶¶^ ECG ^††††^	Acesso fácil aos cuidados especializados de rotina, com consultas ambulatoriais e acesso aos números de telefone, conforme apropriado	QVRS ^||^ (SF ^||||^ -36; MLHFQ ^||||||^ ) Custos médicos Nova hospitalização em até 30 dias Mortalidade	Os escores de QVRS ^||^ em ambos os grupos melhoraram ao longo do ano (p<0.001), mas não houve diferença significativa entre os grupos. Não houve diferenças na mortalidade (p=0.575), na taxa de readmissão (p =0.627) ou nos pagamentos por internações e idas ao pronto socorro entre os grupos
Cui, et al. ^( 60 )^ 2013 Canadá	Indivíduos com diagnóstico de IC* n:174 (GC ^†^ :55| GI ^‡^ Telefone:61 | GI ^‡^ Telemonitoramento:58) Seguimento: 12 meses	Programa de linhas de saúde, composto por: atendimento de enfermagem com orientações em saúde para autogestão e ferramentas de educação em saúde. Além do monitoramento domiciliar para pacientes do GI ^‡^ e telemonitoramento	Ligações telefônicas de enfermagem Monitores de PA ^¶¶^ Balanças Ferramentas de educação em saúde	Os cuidados usuais não foram descritos	Custos e cuidados em saúde QVRS ^||^ (SF ^||||^ -36) Autocuidado ( *Revised Self-Care Behavior scale* ) Satisfação ( *Client Satisfaction Questionnaire* )	Ambas as intervenções foram mais eficazes e menos dispendiosas do que o tratamento padrão. Houve melhoria significativa no comportamento de autocuidado e na QVRS ^||^ (p<0.05), com pontuações de utilidade mais altas e estatisticamente significativas nos grupos de intervenção
Hawkes, et al. ^( 61 )^ 2012 Austrália	Indivíduos com diagnóstico de IAM ^||||||||^ n:430 (GC ^†^ :215 | GI ^‡^ :215) Seguimento: 6 meses	*Coaching* telefônico sobre o manejo dos fatores de risco para doenças cardíacas. Recurso educacional “ *My Heart My Life* ”	Linhas telefônicas de telemedicina ou de suporte	Recurso educacional “M *y Heart My Life* ” e *newsletter* física informativa a cada três meses	QVRS ^||^ (SF ^||||^ -36) Atividade física ( *Active Australia Survey* ) Satisfação Fatores de risco para doenças cardíacas	Em 6 meses, o GI ^‡^ demonstrou escores maiores de QVRS ^||^ em comparação com o GC ^†^ no componente mental (intervalo de confiança 95% 0,5 a 4,9; p = 0,02), físico (intervalo de confiança 95% 0,1 a 4,5; p = 0,04) e emocional (intervalo de confiança 95% 0,2 a 5,2; p = 0,03). Além disso, o GI ^‡^ apresentou maior probabilidade de ser suficientemente ativo (p = 0.02), manter um peso saudável (p = 0.05) em comparação com o GC ^†^
Seto, et al. ^( 62 )^ 2012 Canadá	Indivíduos com diagnóstico de IC* n: 100 (GC ^†^ :50 | GI ^‡^ :50) Seguimento: 6 meses	Sistema de telemonitoramento, além do atendimento-padrão	Sistema de telemonitoramento: Registros diários de peso, de PA ^¶¶^ e de ECG ^††††^ Questionário de sintomas no celular Envio de mensagens *Website* Ligação telefônica de lembrete Envio de e-mails de alerta ao cardiologista Contato por telefone com o paciente	Cuidados regulares, incluindo visitas clínicas programadas e educação sobre IC* durante as consultas. Eles também tiveram acesso ao suporte telefônico, mas não receberam intervenções específicas do estudo além desses cuidados habituais	Níveis de peptídeo natriurético cerebral Autocuidado ( *Self-Care of Heart Failure Index)* QVRS ^||^ (MLHFQ ^||||||^ )	O GI ^‡^ apresentou uma melhoria significativa na QVRS ^||^ (p = 0,05), além de maior manutenção do autocuidado (p = 0,03), comparado ao GC ^†^ . Ambos os grupos tiveram melhorias nos níveis de peptídeo natriurético cerebral, manejo do autocuidado e na fração de ejeção ventricular
Blasco, et al. ^( 63 )^ 2012 Espanha	Indivíduos sobreviventes de SCA ^¶^ n: 203 (GC ^†^ :101 | GI ^‡^ :102) Seguimento: 12 meses	Telemonitoramento e envio de mensagens de texto com recomendações	Aplicação *web* para envio de mensagens de texto Telefone celular Envio de recomendações via celular Monitor de PA ^¶¶^ e glicosímetro	Três consultas com cardiologista; recomendações escritas e verbais sobre prevenção de doenças cardiovasculares	QVRS ^||^ (SF ^||||^ -36) Ansiedade ( *State - Trait Anxiety Inventory* ) Melhora dos fatores de risco cardiovascular	Não houve diferenças significativas entre os grupos nos escores de QVRS ^||^ no início ou no final do estudo. Mas no domínio de “saúde física” do SF ^||||^ -36, houve aumento de 2,8 pontos no GI ^‡^ (p = 0,011). O GI ^‡^ teve maior probabilidade de experimentar melhoria em seu perfil de fator de risco cardiovascular (risco relativo 1.4; intervalo de confiança 95% 1.1-1.7) do que os pacientes no GC ^†^ (p = 0.010)
Koehler, et al. ^( 64 )^ 2011 Alemanha	Indivíduos com diagnóstico de IC* n:710 (GC ^†^ :356|GI ^‡^ :354) Seguimento: 12 meses	Sistema de autogerenciamento remoto baseado em telemedicina	Sistema de telemonitoramento remoto, composto por: Assistente digital pessoal ECG ^††††^ Balança Monitor de PA ^¶¶^ Central de telemedicina, com suporte médico 24 horas	Os cuidados usuais não foram detalhados, foram apenas citados como recomendações-padrão das diretrizes para o tratamento da IC*	Mortalidade Hospitalizações Depressão (PHQ-9 ^§§§§^ ) QVRS ^||^ (SF ^||||^ -36)	Os resultados do estudo mostraram que a intervenção não teve impacto significativo na mortalidade por todas as causas nem na mortalidade cardiovascular ou hospitalização por IC* em comparação com GC ^†^ . Além disso, houve uma melhoria significativa na pontuação de funcionamento físico no SF ^||||^ -36 no GI ^‡^ ao longo de todo o período do estudo (p<0,05).
Baker, et al. ^( 65 )^ 2011 Estados Unidos da América	Indivíduos com diagnóstico de IC* n: 605 (GC ^†^ : 303 | GI ^‡^ : 302) Seguimento: 1 mês	Teleorientação para o autocuidado, iniciada com sessão educacional de 40 minutos sobre o manejo da IC*	Chamadas telefônicas de acompanhamento, orientação e educação em saúde	Sessão educacional de 40 minutos sobre o manejo da IC*, os demais cuidados usuais não foram detalhados	QVRS ^||^ (HFSS ^¶¶¶¶^ ) Conhecimento ( *Improving Chronic Illness Care Evaluation* )	Não houve mudanças significativas na QVRS ^||^ do GC ^†^ do início ao final do seguimento, mas no GI ^‡^ houve diferença de 58,6 (±22,2) no início para 65,3 (±22,4) ao final de 30 dias (p = 0,001). O GI ^‡^ apresentou maior aumento de conhecimento geral (0,70 vs. 0,30, p = 0,008) e na autoeficácia (0,4 vs. 1,0, p = 0,006)
Copeland, et al. ^( 66 )^ 2010 Estados Unidos da América	Indivíduos com diagnóstico de IC* n:458 (GC ^†^ :238|GI ^‡^ :220) Seguimento: 12 meses	Programa de Gerenciamento de Doenças por Telefone, envolvendo a realização de chamadas telefônicas programadas por enfermeiros para fornecer educação, *coaching* para mudança de comportamento e monitoramento de sintomas	Chamadas telefônicas programadas por enfermeiros Plano individualizado de autogestão Linha de aconselhamento de enfermagem disponível 24 horas Alertas por fax para os médicos sobre sinais de descompensação	Os cuidados habituais não foram descritos	QVRS ^||^ (SF ^||||^ -8) Custos em saúde Sobrevida Adesão e satisfação com os cuidados	Após um ano, não houve diferenças clínicas significativas entre os grupos. Os custos relacionados à IC* e os custos globais foram mais elevados no GI ^‡^ . Este grupo também relatou melhor adesão ao monitoramento do peso e às recomendações de exercícios. Não houve diferenças na QVRS ^||^ , uso de internação ou sobrevivência entre os grupos
Balk, et al. ^( 67 )^ 2008 Holanda	Indivíduos com diagnóstico de IC* n: 214 (GC ^†^ :113| GI ^‡^ :101) Seguimento: 9,6 meses	Sistema Motiva ^®^ de educação em saúde e autogerenciamento	Canal de televisão com material educativo Lembretes de medicamentos Questionários relacionados à saúde SMS ^§§^ motivacionais Telemonitoramento de PA ^¶¶^ e peso Contato telefônico por enfermeiras	Acompanhamento-padrão oferecido pelos cardiologistas e enfermeiros de IC* conforme a prática local	Dias de internação/ano QVRS ^||^ (SF ^||||^ -36; MLHFQ ^||||||^ ) Conhecimento da doença Autocuidado ( *European Heart Failure Self- care Behaviour Scale* )	Não houve diferenças significativas para os desfechos primários, QVRS ^||^ ou comportamento de autocuidado. No entanto, houve um aumento maior no conhecimento sobre IC* no GI ^‡^ em comparação com o GC ^†^ (p<0.001)
Schwarz, et al. ^( 68 )^ 2008 Estados Unidos da América	Indivíduos com diagnóstico de IC* ou cuidadores n:102 (GC ^†^ :51| GI ^‡^ :51) Seguimento: 3 meses	Sistema de monitoramento remoto, associado ao acompanhamento regular por parte de profissionais de saúde	Sistema de telemonitoramento vinculado à linha telefônica do paciente: Medida diária de peso Questionário de sintomas, adesão medicamentosa e consumo de sódio Contato telefônico com cuidador, se necessário	Os cuidados usuais não foram detalhados, sendo citados apenas como cuidados pós-alta	Novas hospitalizações Visitas à emergência Custos QVRS ^||^ (MLHFQ ^||||||^ )	O estudo piloto não encontrou diferenças estatisticamente significativas no GI ^‡^ em relação às readmissões hospitalares, visitas ao departamento de emergência, custos ou risco de readmissão hospitalar. A QVRS ^||^ melhorou significativamente em ambos os grupos na visita de acompanhamento de 90 dias (p<0,0001)
Wakefield, et al. ^( 69 )^ 2008 Estados Unidos da América	Indivíduos com diagnóstico de IC* n:148 (GC ^†^ :49|GI ^‡^ Vídeo:52| GI ^‡^ Telefone:47) Seguimento: 12 meses	Programa de telessaúde, baseado em acompanhamento telefônico ou por vídeo, monitoramento de sintomas e educação em saúde	*Checklist* de revisão de sintomas Controle diário de peso, PA ^¶¶^ e circunferência do tornozelo Contato telefônico Contato por chamada de vídeo Treinamentos de habilidades comportamentais Estratégias para maximizar o automonitoramento e autoeficácia Revisão e reforço dos planos de alta	Orientação de alta hospitalar padrão do serviço de saúde e contato telefônico com o enfermeiro gestor de caso, se necessário	Número de readmissões Tempo até a primeira readmissão Atendimentos em unidades de emergência Sobrevida QVRS ^||^ (MLHFQ ^||||||^ )	Não houve diferença em dias de internação ou atendimento de emergência entre os 3 grupos. Para todos os grupos, as pontuações de QVRS ^||^ melhoraram ao longo do tempo (F = 8.90, p = 0.0002). A magnitude da mudança foi maior no GI ^‡^ Telefone, seguido pelo GI ^‡^ Vídeo e GC ^†^
Woodend, et al. ^( 70 )^ 2008 Canadá	Indivíduos com diagnóstico de IC* ou angina n:249 (GC ^†^ :125|GI ^‡^ :124) Seguimento: 12 meses	Programa de telemonitoramento domiciliar composto por vídeo consultas, monitoramento de parâmetros vitais, suporte telefônico e conteúdo educacional estruturado	Equipamentos de monitoramento domiciliar: Balanças Monitor de PA ^¶¶^ ECG ^††††^ de 12 derivações Vídeoconsultas com enfermeiro Registro eletrônico dos dados Linha de aconselhamento de enfermagem disponível 24 horas	Tratamento habitual: cuidados prestados a pacientes com angina ou IC* que recebem alta hospitalar, sem maiores detalhamentos	Status funcional (MLHFQ ^||||||^ ; *Seattle Angina Questionnaire* ) QVRS ^||^ (SF ^||||^ -36) Uso de recursos de saúde (hospitalizações, dias de internação, idas ao pronto socorro)	A QVRS ^||^ foi melhor nos pacientes com IC* do GI ^‡^ em comparação com o GC ^†^ em cinco das oito subescalas do SF ^||||^ -36 aos 3 meses (p < 0,05). As diferenças mais significativas na QVRS ^||^ entre os dois grupos foram detectadas neste período. Pacientes em ambos os grupos demonstraram melhorias significativas na QVRS ^||^ , ao longo do tempo, em todas as subescalas do SF ^||||^ -36
López Cabezas, et al. ^( 71 )^ 2006 Espanha	Indivíduos com diagnóstico de IC* n: 134 (GC ^†^ : 64 | GI ^‡^ :70) Seguimento: 12 meses	Programa de orientações em saúde liderado por farmacêutico, com suporte telefônico para dúvida ou problemas	Material educacional, escrito e audiovisual, sobre os sintomas e a patogênese da IC*; Chamadas telefônicas pelo farmacêutico	Consultas regulares com cardiologista e farmacêutico	Reinternações Adesão ao tratamento QVRS ^||^ (EQ-5D ^‡‡^ ) Satisfação do paciente	No GI ^‡^ , houve maior adesão ao tratamento (85,0 vs. 73,9%), menor número de dias de internação hospitalar (GC ^†^ 9.6 vs. GI ^‡^ 5.9) e maior satisfação (p = 0,026) quando comparado ao GC ^†^ . Houve redução significativa de readmissões hospitalares no GI ^‡^ (54% no início vs. 32% ao final), mas não houve diferenças significativas na QVRS ^||^ .
Riegel et al. ^( 72 )^ 2006 Estados Unidos da América	Indivíduos com diagnóstico de IC* n: 134 (GC ^†^ : 65 | GI ^‡^ : 69) Seguimento: 6 meses	Gerenciamento de caso por telefone, com foco em educação, monitoramento e orientação, além de envio de relatórios e contato com a equipe médica	*Software* de suporte à decisão ( *At Home with Heart Failure* ) Envio mensal de material educativo impresso, pelo correio, aos pacientes Suporte telefônico	Cuidado usual não padronizado baseado em educação sobre o manejo da IC*	Hospitalizações, dias no hospital e custos de cuidados agudos (por IC* e por todas as causas) Readmissões múltiplas Mortalidade por todas as causas QVRS ^||^ (EQ-5D ^‡‡^ )	Não foram encontradas diferenças significativas entre os grupos na taxa de readmissão, dias de internação, custo do cuidado, mortalidade, QVRS ^||^ ou depressão
Benatar, et al. ^( 73 )^ 2003 Estados Unidos da América	Indivíduos com diagnóstico de IC* n:216 (GC ^†^ :108| GI ^‡^ :108) Seguimento: 3 meses	Sistema de telemonitoramento domiciliar, baseado em dispositivos de autogerenciamento, com avaliações por telefone e com definição de metas clínicas individuais	Dispositivos de monitoramento domiciliar para medir peso, PA ^¶¶^ , FC** e nível de saturação de oxigênio Equipe de telegestão com contatos via ligações telefônicas	Os cuidados usuais eram compostos por acompanhamento de saúde via centro e visitas de enfermagem domiciliares	QVRS ^||^ (MLHFQ ^||||||^ | *70-item Quality of Life Index – Cardiac* ) Ansiedade e depressão (HADS*****) Autoeficácia ( *Heart Failure Self-Efficacy* *scale 30* ) Hospitalizações Custos em saúde	O grupo GI ^‡^ teve menos readmissões por IC* (13 vs. 24; P≤0,001), com um tempo de permanência hospitalar mais curto (49,5 vs. 105,0 dias; P≤0,001) e custos hospitalares menores aos 3 meses ($65.023 vs. $177.365; p≤0,02). Ambos os grupos experimentaram uma melhoria na QVRS ^||^ após a intervenção
Barnason, et al. ^( 74 )^ 2003 Estados Unidos da América	Indivíduos com IC* isquêmica, submetidos à revascularização miocárdica n:35 (GC ^†^ :17| GI ^‡^ :18) Seguimento: 3 meses	*Health Buddy* : avaliação dos sintomas e educação sobre recuperação pós-revascularização, manejo de sintomas, funcionamento e adesão à modificação dos fatores de risco da DAC ^‡‡‡^	Dispositivo de telecomunicação conectado ao telefone do paciente ( *Health Buddy* ) *Website* de armazenamento dos dados coletados	Educação e aconselhamento sobre cuidados para recuperação pós-cirurgia, para autocuidado e modificação dos fatores de risco da DAC ^‡‡‡^ , prescrição de exercícios domiciliares, fornecidos a todos os pacientes submetidos à revascularização antes da alta hospitalar	Autoeficácia (Escala de Expectativa de Eficácia de Barnason) QVRS ^||^ (MOS SF-36 ^†††††^ )	O GI ^‡^ teve pontuações médias maiores de autoeficácia ajustadas (p < 0,05), níveis médios ajustados de funcionamento físico, geral, mental e de vitalidade (p < 0.05), adesão ao exercício significativamente maior (p < 0,01) e menor estresse relatado (p < 0,01) após três meses de cirurgia, quando comparado ao GC ^†^ . Houve uma melhoria significativa nos escores de dor e funcionamento emocional ao longo do tempo (p < 0.05)

*IC = Insuficiência cardíaca; ^†^GC = Grupo controle; ^‡^GI = Grupo intervenção; ^§^NYHA = *New York Heart Association*; ^||^QVRS = Qualidade de vida relacionada à saúde; ^¶^SCA = Síndrome coronariana aguda; **FC = Frequência cardíaca; ^††^VO_2_ = Volume de Oxigênio; ^‡‡^EQ-5D = *EuroQol-5 dimension-5 levels*; ^§§^SMS = Mensagens de texto curtas; ^||||^SF - *Short form health survey*; ^¶¶^PA = Pressão arterial; ***WHO-5 = Índice de Bem-estar da *World Health Organization-5 itens*; ^†††^KCCQ = *Kansas City Cardiomyopathy Questionnaire;* ^‡‡‡^DAC = Doença arterial coronariana; ^§§§^EQ-VAS = *EuroQol Visual Analogue Scale*; ^||||||^MLHFQ = *Minnesota Living With Heart Failure Questionnaire*; ^¶¶¶^TC6M = Teste de caminhada de seis minutos; ****HeartQoL = *Health-related quality of life questionnaire*; ^††††^ECG = Eletrocardiograma; ^‡‡‡‡^FA = Fibrilação atrial; ^§§§§^PHQ-9 = *Patient Health Questionnaire-9 items;* ^||||||||^IAM = Infarto agudo do miocárdio; ^¶¶¶¶^HFSS = *Heart Failure Symptom Scale*; *****HADS = *Hospital Anxiety and Depression Scale;* ^†††††^MOS SF-36 = *Medical Outcomes Short-Form Health Survey-36 items*

Ademais, a presente revisão sistemática revela que a literatura científica sobre o tema está em constante expansão, especialmente com a inclusão de estudos de alta qualidade metodológica. Os primeiros artigos foram publicados em 2003^(73-74)^, e desde então não houve redução no número de publicações. Pelo contrário, observou-se um aumento significativo de estudos, especialmente após o advento da pandemia, quando os resultados relacionados ao uso de telemedicina ganharam ainda mais relevância.

Os artigos que cumpriram os critérios de inclusão foram submetidos a uma avaliação crítica de sua qualidade metodológica (n=44). Nesse contexto, os resultados variaram de 53,85% a 92,31% de congruência com o instrumento utilizado^(29)^. É importante observar que as principais inconsistências foram encontradas no cegamento dos avaliadores de resultados, dos aplicadores da intervenção e dos pacientes [o detalhamento completo das avaliações pode ser consultado no Material Suplementar (https://doi.org/10.48331/scielodata.MI2JBD)].

Em relação às características da população dos estudos incluídos, a maioria dos pacientes era acompanhada em função de insuficiência cardíaca (IC) (68,18%, n=30)^(37,43-46,51-53,59-60,62,65-67,63-64,66,68-70,72-74)^, síndrome coronariana aguda (SCA) (15,22%, n=7)^(32-33,36,40,58,61,63)^ ou doença arterial coronariana (DAC) (10,64%, n=5)^(35,40,42,54,57)^. Em quatro estudos^(40,43,54,71)^, a intervenção era voltada para uma população mista de pacientes com diferentes doenças cardíacas.

Ainda, no que diz respeito aos recursos e intervenções adotadas, as modalidades de telemedicina variaram consideravelmente, com destaque para as intervenções híbridas que empregam múltiplas ferramentas para maximizar a eficácia do tratamento. O telemonitoramento foi a intervenção mais frequentemente utilizada, aparecendo em 31 estudos (68,89%)^(31-32,34-35,38-46,48-49,51-53,56-60,62-64,67,68,70,73-74)^. Além do telemonitoramento, outra intervenção que se destacou como um recurso de suporte amplamente utilizado foi a ligação telefônica, que esteve presente em 23 artigos (51,11%)^(34,36,37,39,41-42,45,47,51-52,57,60-62,65-73)^. Na maioria dos casos, o contato por telefone foi utilizado como suporte complementar^(34,39,41-42,45,47,51-52,57,60-62,67-68,70,73)^, auxiliando na comunicação e no acompanhamento dos pacientes. No entanto, em algumas situações as ligações constituíram a principal forma de intervenção^(36-37,65-66,69,71-72)^, destacando sua importância em cenários nos quais outras tecnologias podem não estar disponíveis ou ser viáveis.

Além dessas intervenções, a telerreabilitação foi identificada em 13 estudos (28,88%)^(32,35,39,41-42,47,50,52,54-58)^. Dentre as abordagens híbridas, a combinação de telemonitoramento e telerreabilitação se sobressai, presente em nove estudos (20%)^(32,35,39,41-42,52,56-58)^. Essa abordagem oferece monitoramento em tempo real dos sinais vitais e do progresso do paciente, bem como da prescrição remota de exercícios. Por sua vez, o uso de serviços de mensagens curtas (SMS) isolada é menos comum, citada em apenas um artigo^(33)^, mas frequentemente combinado com outras intervenções^(36,43,47,54-55,62)^ devido à sua capacidade de enviar lembretes, instruções e motivações contínuas.

Dentre os 44 estudos analisados, os desfechos primários ou secundários incluíram uma comparação pré e pós-intervenção da QVRS entre os grupos intervenção e controle. Nesse sentido, observou-se uma diversidade dentre os instrumentos utilizados, com 81,81% (n=36) dos estudos optando pelo uso de um único questionário^(36,38,40,66-67)^, enquanto 11,36% (n=5) empregaram dois questionários^(50,55,59,67,73)^ e 6,81% (n=3) recorreram à aplicação de três questionários^(34-35,43)^.

Entre as escalas empregadas, a escala *Short Form Health Survey*-36 (SF-36) foi a mais comum, sendo adotada em 17 (38,63%) dos ensaios clínicos^(34,39,41-44,52,55,59-61,63-64,67,70-74)^, enquanto 12 (26,66%) estudos adotaram a escala *Minnesota Living With Heart Failure Questionnaire* (MLHFQ)^(38,45-48,50,59,62,67-69,73)^. Já as variações do questionário *EuroQol* foram empregadas em dez estudos (24,44%)^(32,35-36,43,49-50,55,58,71-72)^.

A avaliação GRADE^(30)^ revelou um sério risco de viés, pois a heterogeneidade nos relatórios de medição dos efeitos impediu a realização de uma análise agrupada. A Figura 3 apresenta o perfil de evidências, separando os estudos por questionários utilizados e mostrando que a certeza das evidências variou de baixa a muito baixa.

Em relação aos resultados de QVRS, nenhum dos estudos selecionados apresentou efeito de piora da QVRS do GI baseado em telemedicina ao longo do seguimento. Além disso, 45,45% (n = 20) dos estudos tiveram relação positiva, com significância estatística, nos escores de QVRS no grupo intervenção^(33-34,41,45,47-49,51,54-55-58-60-65-68-70)^. Entretanto, a análise abrangente dos estudos revelou que 11,36% (n = 5) não observaram nenhum impacto na QVRS ao longo do acompanhamento^(36,38,40,66-67)^. Em contrapartida, 43,18% (n = 19) demonstraram melhorias positivas na QVRS, sem alcançar diferença estatisticamente significativa entre os grupos^(31-32,35,37,39,42-44,46,50,52-53,56,57,59,71,74)^. A Tabela 1 fornece uma síntese detalhada dos resultados em QVRS dos estudos avaliados.

**Figura 3 -:** Perfil de evidência GRADE

Avaliação de Certeza							N ^o^ de pacientes		Efeito		Garin, et al	Importância
N ^o^ dos estudos	Delineamento do estudo	Risco de viés	Inconsistência	Evidência indireta	Imprecisão	Outras considerações	Telemedicina	Cuidado usual	Relativo (95% IC*)	Absoluto (95% IC*)		
Qualidade de vida relacionada à saúde (seguimento: média 8.9 meses; avaliado com: EuroQol (EQ-5D ^†^ | EQ-5D-5L ^‡^ | EQ-5D-Y ^§^ ); Escala de: 0 para 100)												
7	ensaios clínicos randomizados	muito grave ^||^	não grave	não grave	não grave	nenhum	649	613	-	0 (0 para 0)	⨁⨁◯◯ | CRÍTICO | Baixa |	
Qualidade de vida relacionada à saúde (seguimento: média 7.8 meses; avaliado com: MLHFQ ^¶^ | HFSS**; Escala de: 0 para 105)												
13	ensaios clínicos randomizados	muito grave ^††^	não grave	não grave	grave ^‡‡^	nenhum	1309	1102	-	0 (0 para 0)	⨁ ◯◯◯ | CRÍTICO | Muito baixa |	
Qualidade de vida relacionada à saúde (seguimento: média 8.8 meses; avaliado com: SF ^§§^ -36 | SF ^§§^ -12 | SF ^§§^ -8; Escala de: 0 para 100)												
16	ensaios clínicos randomizados	muito grave ^||||^	não grave	não grave	não grave	nenhum	2850	2800	-	0 (0 para 0)	⨁ ⨁ ◯◯ | CRÍTICO | Baixa |	
Qualidade de vida relacionada à saúde (seguimento: média 10.5 meses; avaliado com: KCCQ ^¶¶^ ; Escala de: 0 para 100)												
4	ensaios clínicos randomizados	muito grave***	não grave	não grave	não grave	nenhum	1497	1528	-	0 (0 para 0)	⨁ ⨁ ◯◯ | CRÍTICO | Baixa |	
Qualidade de vida relacionada à saúde (seguimento: média 9 meses; avaliado com: HeartQol ^†††^ ; Escala de: 0 para 3)												
2	ensaios clínicos randomizados	muito grave ^‡‡‡^	não grave	não grave	não grave	nenhum	127	126	-	0 (0 para 0)	⨁ ⨁ ◯◯ | CRÍTICO | Baixa |	
Qualidade de vida relacionada à saúde (seguimento: média 3 meses; avaliado com: MacNew; Escala de: 1 para 7)												
2	ensaios clínicos randomizados	muito grave ^‡‡‡^	não grave	não grave	não grave	nenhum	67	64	-	0 (0 para 0)	⨁ ⨁ ◯◯ | CRÍTICO | Baixa |	

*IC = Intervalo de confiança; ^†^EQ-5D = *EuroQol-5 Dimension*; ^‡^EQ-5D-5L= *EuroQol-5 dimension-5 levels*; ^§^EQ-5D-Y = *EuroQol-5 dimension-young*; ^||^Muito grave = Sete estudos com algumas preocupações em relação ao viés de randomização (ocultação de alocação, cegamento dos profissionais que realizaram as intervenções) e cinco estudos com viés relacionado ao cegamento dos avaliadores de resultados; ^¶^MLHFQ = *Minnesota Living with Heart Failure Questionnaire*; **HFSS = *Heart Failure Symptom Scale*; ^††^Muito grave = Doze estudos com algumas preocupações em relação ao viés de randomização (ocultação de alocação, cegamento dos profissionais que realizaram as intervenções e dos avaliadores de resultados); ^‡‡^Grave= Sete estudos com ensaio com alto risco de imprecisão devido à falta de dados de resultados; ^§§^SF = *Short form health survey*; ^||||^Muito Grave = Quinze estudos com algumas preocupações em relação ao viés de randomização (ocultação de alocação, cegamento dos profissionais que realizaram as intervenções) e 11 estudos com viés relacionado ao cegamento dos avaliadores de resultados; ^¶¶^KCCQ= *Kansas City Cardiomyopathy Questionnaire*; ***Muito grave = Quatro estudos com algumas preocupações em relação ao viés de randomização (ocultação de alocação, cegamento dos profissionais que realizaram as intervenções) e três estudos com viés relacionado ao cegamento dos avaliadores de resultados; ^†††^HeartQol = *Health-related Quality of life questionnaire*; ^‡‡‡^Muito grave = Dois estudos com algumas preocupações em relação ao viés de randomização (ocultação de alocação, cegamento dos profissionais que realizaram as intervenções)

**Tabela 1 -:** Síntese dos resultados de QVRS (n = 44). São Paulo, Brasil, 2024

Estudo	Seguimento (meses)	Instrumento	Grupo	n	Escore Inicial Média (DP*)	Escore Final Média (DP*)	p-valor	Mudança Média (DP*)	Diferença entre os grupos (p-valor)
Choi, et al. ^( 31 )^	3	MacNew	GC ^†^	38	5,45 (0,86)	5,34 (1,09)	-	-	0,771
			GI ^‡^	38	5,62 (0,82)	5,62 (0,78)	-	-	
Dalli Peydró, et al. ^( 32 )^	10	EQ-5D-5L ^§^	GC ^†^	28	70 (65-85)	80 (70-90)	0,008	-	>0,800
			GI ^‡^	31	75 (60-90)	88 (68-90)	0,064	-	
Chow, et al. ^( 33 )^	12	SF ^||^ -12	GC ^†^	708	-	PS ^¶^ : 47,6(-) MCS**: 51,2 (-)	-	-	PS ^¶^ : 0,045 MCS**: 0,204
			GI ^‡^	716	-	PS ^¶^ : 47,6 (9,5) MCS**: 48,9 (9,6)	-	-	
Voller, et al. ^( 34 )^	12	KCCQ ^††^	GC ^†^	319	59,3 (24,0)	-	-	-	<0,01
			GI ^‡^	302	59,6 (23,0)	-	-	-	
Brouwers, et al. ^( 35 )^	12	EQ-5D-5L ^§^	GC ^†^	147	0,815 (0,010)	0,848 (0,016)	-	-	0,002
			GI ^‡^	153	0,814 (0,011)	0,851 (0,015)	-	-	
Maddison, et al. ^( 36 )^	13	EQ-5D ^‡‡^	GC ^†^	153	64 (22)	-	-	-	-
			GI ^‡^	153	62 (22)	-	-	-	-
Collins, et al. ^( 37 )^	3	KCCQ ^††^	GC ^†^	245	-	-	-	-	0,25
			GI ^‡^	246	-	-	-	-	
Clays, et al. ^( 38 )^	6	MLHFQ ^§§^	GC ^†^	22	30,0 (13,5)	-	0,58	1,7 (13,8)	0,5
			GI ^‡^	34	32,1 (22,9)	-	0,7	-1,0 (14,4)	
Batalik, et al. ^( 39 )^	3	SF ^||^ -36	GC ^†^	28	50,9 (8,8)	61,5 (7,1)	0,01	-	0,56
			GI ^‡^	28	53,1 (6,7)	62 (7)	0,01	-	
Lunde, et al. ^( 40 )^	12	HeartQoL ^||||^ EQ-5D ^‡‡^	GC ^†^	147	2,48 (0,54)	2,57 (0,51)	-	-	<0,05
			GI ^‡^	153	2,43 (0,59)	2,64 (0,51)	-	-	
			GC ^†^	147	72 (14)	75 (12)	-	-	<0,001
			GI ^‡^	153	69 (18)	78 (16)	-	-	
Piotrowicz, et al. ^( 41 )^	14-26	SF ^||^ -36	GC ^†^	425	88,8 (14,1)	88,9 (14,4)	-	-	0,008
			GI ^‡^	425	89,7 (12,6)	91,2 (12,8)	-	-	
Ávila, et al. ^( 42 )^	3	SF ^||^ -36	GC ^†1^	30	73,3 (15,1)	76,4 (16,4)	-	-	0,06
			GC ^†2^	30	79,8 (16,1)	82,6 (15,8)	-	-	
			GI ^‡^	30	82,2 (13,3)	82,6 (13)	-	-	
Cáceres, et al. ^( 43 )^	6	SF ^||^ -36 AFEQT ^¶¶^ EQ-5D ^‡‡^	GC ^†^	123	PS ^¶^ : 47,6 (9,5) MCS**: 48,9 (9,6)	PS ^¶^ : 50,1 (8,5) MCS**: 52,7 (7,5)	-	-	PS ^¶^ : 0,37 MCS**: 0,74
			GI ^‡^	115	PS ^¶^ : 49,6 (8,6) MCS**: 50,8 (8,5)	PS ^¶^ : 52,5 (9,1) MCS**: 53,1 (8,6)	-	-	
			GC ^†^	123	64,8 (25,2)	80,2 (20,9)	-	-	0,09
			GI ^‡^	115	66,3 (21)	79 (20,3)	-	-	
			GC ^†^	123	0,85 (0,21)	0,91 (0,13)	-	-	0,98
			GI ^‡^	115	0,88 (0,16)	0,94 (0,14)	-	-	
Cichosz, et al. ^( 44 )^	12	SF ^||^ -36 KCCQ ^††^	GC ^†^	154	PS ^¶^ : 40,3 (9,2) MCS**: 48,9 (11,4)	PS ^¶^ : 40,67 (10,2) MCS**: 46,65 (12,1)	-	-	NS***
			GI ^‡^	145	PS ^¶^ : 40,3 (9) MCS** 47,4 (10)	PS ^¶^ : 40,58 (9,7) MCS**: 50,01 (11,5)	-	-	<0,05
			GC ^†^	154	62,5 (20,4)	65,56 (21,2)	-	-	<0,01
			GI‡	145	64,7 (18,8)	66,31 (20,7)	-	-	
Mizukawa, et al. ^( 45 )^	24	MLHFQ ^§§^	GC ^†^	19	32,2 (27,8)	-	-	-	0,564
			GI ^‡1^	20	37,3 (22,7)	-	-	-	
			GI ^‡2^	20	47,5 (26,8)	-	-	-	
Pekmezaris, et al. ^( 46 )^	3	MLHFQ ^§§^	GC ^†^	58	59,9	27,8	-	-	0,5
			GI ^‡^	46	62,7	36,3	-	-	
Peng, et al. ^( 47 )^	4	MLHFQ ^§§^	GC ^†^	49	48,77 (12,21)	49,63 (12,39)	-	-	0,072
			GI ^‡^	49	49,43 (12,25)	42,32 (8,83)	-	--	
Wagenaar, et al. ^( 48 )^	12	MLHFQ ^§§^	GC ^†^	150	23,0 (32,5)	26,5	-	-	0,003
			GI ^‡1^	150	24,0 (31,0)	28,3	-	-	
			GI ^‡2^	150	23,0 (27,8)	25,5	-	-	
Guo, et al. ^( 49 )^	3	EQ-5D-Y ^†††^	GC ^†^	96	71,3	69,9	-	-	<0,05
			GI ^‡^	113	86,5	87,2	-	-	
Hwang, et al. ^( 50 )^	6	MLHFQ ^§§^	GC ^†^	29	47 (19)	33 (21)	-	-	-
			GI ^‡^	24	41 (22)	34 (23)	-	-	
Jayaram, et al. ^( 51 )^	6	KCCQ ^††^	GC ^†^	765	58,6 (24,9)	68,5 (26,4)	0,11	-	0,01
			GI ^‡^	756	60,7 (24,1)	72,3 (24,4)	0,01	-	
Piotrowicz, et al. ^( 52 )^	2	SF ^||^ -36	GC ^†^	34	73,6 (25,6)	67,4 (25,9)	NS**	-	0,0001
			GI ^‡^	77	79,0 (31,3)	70,8 (30,3)	0,001	-	
Bekelman, et al. ^( 53 )^	12	KCCQ ^††^	GC ^†^	193	36,9 (14,6)	54,2	-	-	0,97
			GI ^‡^	199	37,9 (13,3)	53,6	-	-	
Frederix, et al. ^( 54 )^	6	HeartQoL ^||||^	GC ^†^	70	2,31 (0,59)	2,32 (0,58)	0,21	-	0,01
			GI ^‡^	70	2,27 (0,63)	2,53 (0,44)	0,01	-	
Maddison, et al. ^( 55 )^	6	SF ^||^ -36 EQ-5D ^‡‡^	GC ^†^	86	PS ^¶^ : 51,9 (5,8) MCS**: 51,53 (8,4)	PS ^¶^ : 51,9(-) MCS**: 54(-)	-	-	PS ^¶^ : 0,2 MCS**: 0,61
			GI ^‡^	85	PS ^¶^ : 51,6 (5,9) MCS**: 52,87 (6,94)	PS ^¶^ : 52,9(-) MCS**: 54,6(-)	-	-	
			GC ^†^	86	0,8 (0,1)	0,83(-)	-	-	0,23
			GI ^‡^	85	0,8 (0,1)	0,86(-)	-	-	
Piotrowicz, et al. ^( 56 )^	2	SF ^||^ -36	GC ^†^	75	81,6 (27,3)	62,2 (26,4)	-	-	NS***
			GI ^‡^	77	79,3 (25,6)	70,5 (25,4)	-	-	
Kraal, et al. ^( 57 )^	3	MacNew	GC ^†^	26	5,2 (0,8)	5,8 (0,7)	-	-	0,498
			GI ^‡^	29	5,7 (0,7)	6,1 (0,5)	-	-	
Varnfield, et al. ^( 58 )^	1,5	EQ-5D ^‡‡^	GC ^†^	60	0,83 (0,8–0,9)	0,82 (0,7-0,9)	0,7	-	0,01
			GI ^‡^	60	0,84 (0,8–0,9)	0,92 (0,9–1,0)	<0,001	-	
Blum, et al. ^( 59 )^	12	MLHFQ ^§§^ SF ^||^ -36	GC ^†^	102	37 (27)	18 (21)	-	-	NS***
			GI ^‡^	104	42 (23)	24 (24)	-	-	
			GC ^†^	102	PS ^¶^ : 35 (11) MCS**: 49 (13)	PS ^¶^ : 38 (11) MCS**: 55 (9)	-	-	-
			GI‡	104	PS ^¶^ : 37 (90) MCS**: 49 (12)	PS ^¶^ : 38 (10) MCS**: 52 (11)	-	-	
Cui, et al. ^( 60 )^	12	SF ^||^ -36	GC ^†^	55	44,61 (23,3)	49,05 (19,59)	-	-	0,5341
			GI ^‡1^	61	45,93 (19,47)	55,63 (27,51)	-	-	
			GI ^‡2^	58	44,73 (17,79)	40,17 (23,38)	-	-	
Hawkes, et al. ^( 61 )^	6	SF ^||^ -36	GC ^†^	215	46,7 (9,6)	46,7 (11,1)	-	0,3	0,73
			GI ^‡^	215	45,8 (10,1)	46,4 (11,5)	-	-	
Seto, et al. ^( 62 )^	6	MLHFQ ^§§^	GC ^†^	50	47,8 (22,6)	47,3 (23,4)	0,9	-	0,05
			GI ^‡^	50	50,3 (29,1)	41,4 (26,7)	0,02	-	
Blasco, et al. ^( 63 )^	12	SF ^||^ -36	GC ^†^	101	-	-	-	-	NS***
			GI ^‡^	102	-	-	-	-	
Koehler, et al. ^( 64 )^	12	SF ^||^ -36	GC ^†^	356	-	51,7 (1,4)	-	0,3	<0,05
			GI ^‡^	354	-	53,8 (1,4)	-	-	
Baker, et al. ^( 65 )^	1	HFSS ^‡‡‡^	GC ^†^	303	64,8 (22,4)	64,1 (22,8)	-	-0,6	<0,001
			GI ^‡^	302	58,6 (22,2)	65,3 (22,4)	-	6,7	
Copeland, et al. ^( 66 )^	12	SF ^||^ -8	GC ^†^	238	-	-	-	-	NS***
			GI ^‡^	220	-	-	-	-	
Balk, et al. ^( 67 )^	9,6	MLHFQ ^§§^	GC ^†^	113	-	-	-	-	NS***
			GI ^‡^	101	-	-	-	-	
Schwarz, et al. ^( 68 )^	3	MLHFQ ^§§^	GC ^†^	51	35,8 (21,5)	27,3 (21,6)	-	-	NS***
			GI ^‡^	51	39,5 (23,3)	27,4 (21,7)	-	-	
Wakefield, et al. ^( 69 )^	12	MLHFQ ^§§^	GC ^†^	49	60,6 (19,3)	56,6 (23,9)	-	-	0,0002
			GI ^‡1^	52	58,4 (22,9)	41,5 (26,9)	-	-	
			GI ^‡2^	47	60,2 (24,8)	54,0 (26,0)	-	-	
Woodend, et al. ^( 70 )^	12	SF ^||^ -36	GC ^†^	125	-	-	-	-	-
			GI ^‡^	124	-	-	-	-	
López Cabezas, et al. ^( 71 )^	12	EQ-5D ^‡‡^	GC ^†^	64	65 (17,6)	60,6 (17,8)	-	-	NS***
			GI ^‡^	70	62,3 (17,3)	64 (15,4)	-	-	
Riegel, et al. ^( 72 )^	6	EQ-5D ^‡‡^ MLHFQ ^§§^	GC ^†^	65	57,1 (16,7)	73,7 (17,4)	-	-	-
			GI ^‡^	69	60,4 (19,9)	73,4 (17,4)	-	-	-
			GC ^†^	65	56,1 (16,7)	12,9 (10,9)	-	-	
			GI ^‡^	69	52,7 (19,6)	12,1 (12,3)	-	-	
Benatar, et al. ^( 73 )^	3	MLHFQ ^§§^	GC ^†^	108	77,17 (8,52)	57,72 (16,24)	0,47	-	<0,01
			GI ^‡^	108	77,92 (10,3)	51,64 (17,36)	0,98	-	
Barnason, et al. ^( 74 )^	3	SF ^||^ -36	GC ^†^	17	60,7	69,1	-	-	-
			GI ^‡^	18	60,7	78,3	-	-	

*DP = Desvio-padrão; ^†^GC = Grupo controle; ^‡^GI = Grupo intervenção; ^§^EQ-5D-5L = *EuroQol-5 dimension-5 levels*; ^||^SF = *Short form health survey;* ^¶^PS = Sumário do componente físico do *36-Item Short Form Survey*;**MCS = Sumário do componente mental do *36-Item Short Form Survey*; ^††^KCCQ = *Kansas City Cardiomyopathy Questionnaire*; ^‡‡^EQ-5D = *EuroQol-5 dimension*; ^§§^MLHFQ = *Minnesota Living With Heart Failure Questionnaire*; ^||||^HeartQoL = *Health-related quality of life questionnaire*; ^¶¶^AFEQT = *Atrial Fibrillation Effect on Quality-of-life;* ***NS = Não significativo; ^†††^EQ-5D-Y= *EuroQol-5 dimension young*; ^‡‡‡^HFSS =*Heart Failure Symptom Scale*

## Discussão

As intervenções de telemedicina focadas no autogerenciamento demonstraram uma relação positiva na melhora da QVRS de pacientes com doenças cardíacas em 45,45% dos estudos incluídos^(33-34,41,45,47-49,51,54-55-58-60-65-68-70)^. Essas intervenções foram majoritariamente aplicadas a indivíduos com IC^(37,43-46,51-53,59-60,62,65-67,63-64,66,68-70,72-74)^, SCA^(32-33,36,40,58,61,63)^ e DAC^(35,40,42,54,57)^. Entre os principais recursos utilizados estavam telemonitoramento, ligações telefônicas, telerreabilitação, SMS e videoconsultas^(31-74)^.

A literatura científica sobre telemedicina tem mostrado um crescimento constante desde a publicação dos primeiros estudos em 2003, com um aumento significativo de pesquisas durante a pandemia de COVID-19^(11)^. Nesta revisão sistemática, foram analisados 44 ensaios clínicos randomizados, envolvendo o total de 12.732 pacientes. Embora houvesse uma leve disparidade numérica entre os grupos controle (6.233) e intervenção (6.499), a distribuição foi considerada balanceada pelos autores dos estudos incluídos. Os estudos foram conduzidos principalmente na Europa (40,91%) e América do Norte (36,36%), com participação significativa também da Oceania (13,64%) e Ásia (9,09%). Essa distribuição geográfica destaca a ampla aceitação e aplicação das tecnologias de telemedicina em diversos contextos culturais e de infraestrutura.

No entanto, a expansão da telemedicina durante a pandemia também evidenciou fraquezas, desigualdades e limitações nos sistemas de saúde que anteriormente poderiam ser menos perceptíveis^(11)^. Apesar da disseminação geográfica positiva entre continentes, identificam-se lacunas geográficas, especialmente em regiões como América Latina e África. Salienta-se que a escassez pode ser atribuída à limitada infraestrutura tecnológica e aos menores investimentos em pesquisa sobre telemedicina nesses locais^(11)^. Isso destaca a necessidade de abordagens multifacetadas para enfrentar esses desafios e garantir uma representação mais abrangente da pesquisa em telemedicina em escala global.

A mensuração e avaliação da QVRS são desafios frequentemente enfrentados em pesquisas científicas devido à natureza multidimensional e às diversas definições associadas a esse conceito^(75)^. Assim, considerando tal complexidade, uma variedade de instrumentos foi desenvolvida e tem sido empregada para avaliar a QVRS^(75)^. De modo geral, na presente revisão, observou-se a utilização de 17 instrumentos diferentes, com o predomínio de um único questionário^(36,38,40,66-67)^para a avaliação do desfecho de QVRS.

Nesse sentido, a escolha entre o uso de instrumentos genéricos e específicos para condições médicas na mensuração dessa variável apresenta vantagens distintas. Os instrumentos genéricos permitem a comparação da QVRS entre diferentes condições de saúde, oferecendo uma visão ampla e comparativa em diversas situações clínicas. Por outro lado, as medidas específicas para condições médicas concentram-se diretamente na avaliação da QVRS relacionada à condição em estudo, tornando-os instrumentos clinicamente mais relevantes para entender o impacto específico da doença na vida dos pacientes^(76)^.

Portanto, o uso de perspectivas duais proporciona interpretações mais completas e complexas na abordagem da QVRS nas pesquisas em saúde, permitindo uma análise mais precisa e informativa dos desafios enfrentados pelos pacientes em diferentes contextos de vida^(76)^. Por conseguinte, nesta revisão apenas nove estudos realizaram a análise de QVRS a partir de dois ou três questionários^(34-35,43,50,55,59,67,73-74)^.

Na população estudada, observou-se uma predominância de pacientes com IC, representando 68,18% do total^(37,43-46,51-53,59-60,62,65-67,63-64,66,68-70,72-74)^. A QVRS nesses pacientes foi avaliada utilizando 10 questionários, incluindo seis específicos para essa população: MLHFQ^(38,45-48,50,59,62,67-69,73)^, KCCQ^(34,51,53,37)^, MacNew^(31)^, HeartQol^(54)^, HFSS^(65)^ e *70-item Quality of Life Index – Cardiac* ^(73)^; e quatro questionários genéricos: SF-36^(34,41,44,52,56,59-60,64,66,67,70,74)^, SF-8^(66)^, WHO-5^(34)^ e ED-5Q^(50,71-72)^. Considerando o contexto da IC, o uso do instrumento MLHFQ é considerado positivo, conforme mostrado em uma revisão sistemática prévia, que o apontou como a escala mais indicada para medir a QVRS nesses indivíduos, tendo a KCCQ como opção secundária^(77)^.

No que diz respeito às repercussões na QVRS dos pacientes, uma parcela significativa dos estudos incluídos (45,45%) evidenciou resultados positivos com significância estatística no grupo de intervenção^(33-34,41,45,47-49,51,54-55-58-60-65-68-70)^. Cerca de 11,36% dos ECRs não indicaram impacto algum^(36,38,40,66-67)^, enquanto outros 43,18% apontaram melhorias na QVRS, embora sem alcançar diferença estatisticamente significativa^(31-32,35,37,39,42-44,46,50,52-53,56,57,59,71,74)^. Essa diversidade de resultados ressalta a complexidade e a natureza multifacetada da QVRS como um desfecho de saúde, destacando a importância de considerar fatores contextuais e específicos de cada intervenção ao avaliar seu impacto na vida dos pacientes^(75-76)^. Além disso, embora a telemedicina seja promissora, sua eficácia varia devido às diferenças metodológicas, características das populações incluídas e tipos de intervenção. Esse achado está alinhado com uma metanálise sobre a efetividade da telemedicina no manejo das DCNT, que indicou uma melhoria na QVRS em estudos sobre doenças cardiovasculares, embora sem significância estatística^(78)^.

Com isso, outro ponto importante a ser destacado é que os resultados das intervenções em telemedicina podem ser equiparados aos cuidados presenciais tradicionais, sem demonstrar inferioridade. Nenhum dos estudos incluídos identificou deterioração na QVRS entre os participantes. Isso sugere que a implementação da telemedicina no cuidado de condições cardiovasculares não acarreta consequências adversas na QVRS dos pacientes e ainda pode gerar benefícios a outros desfechos de saúde, como repercussões na atividade física^(32,34,39-42,47,52,54,57,61)^, no uso de recursos de saúde (hospitalizações, dias de internação e idas à emergência)^(45,69,71,73)^, nas repercussões na saúde mental^(38,46,53,63,73)^ e no autocuidado^(31,38,48,60,62)^.

Em relação às intervenções utilizadas nos estudos, observou-se que o telemonitoramento foi o recurso mais abrangente. Quando utilizado de forma isolada, apresentou repercussões positivas na QVRS em apenas 36,36% (4 de 11) dos estudos^(48-49,63-64)^. Da mesma forma, os ECRs que abordaram apenas ligações telefônicas demonstraram repercussões positivas na QVRS em apenas 33,33% (1 de 3)^(61)^. No entanto, intervenções combinadas, como a associação do telemonitoramento com ligações telefônicas, vídeo consultas ou SMS, mostraram diferenças estatisticamente significativas em 7 de 11 estudos (63,63%)^(34,45,51,60,62,68,70)^. No caso da telerreabilitação, que sempre estava associada a outro recurso listado, observou-se repercussão positiva na QVRS em 38,46% (5 de 13) dos ECRs^(41,47,54-55,58)^. Esses resultados mistos também foram refletidos em uma revisão sistemática de 19 estudos sobre telemonitoramento na insuficiência cardíaca, que identificou uma heterogeneidade nos estudos que mensuraram a QVRS, bem como uma qualidade metodológica duvidosa e limitações de amostra^(79)^.

Ainda em relação à telerreabilitação, houve melhora da QVRS em oito estudos, mas sem superioridade em relação ao cuidado habitual^(32,35,39,42,50,52,56-57)^, o que é corroborado por uma revisão sistemática comparando a telerreabilitação à reabilitação baseada em centro, a qual demonstrou que essa intervenção é tão efetiva quanto na melhora da QVRS^(23)^. Nesse sentido, considerando que a telerreabilitação cardíaca parece aceitável para os pacientes e comparável ao cuidado usual, pode ser uma forma eficaz de aumentar o alcance e a adesão à reabilitação^(80)^. Entretanto, estudos futuros são necessários para explorar como a telerreabilitação pode ser integrada de forma eficaz nos sistemas de saúde e como os profissionais de saúde podem ser treinados e apoiados para fornecer esse tipo de cuidado de forma adequada.

Ainda, no que tange ao tempo de acompanhamento dos estudos, observou-se uma variação significativa entre os ECRs, com períodos que variam de um a 26 meses, o que pode contribuir para a heterogeneidade dos resultados. Considerando o impacto positivo e com significância estatística na QVRS, 10 estudos realizaram acompanhamento por até seis meses^(47,49,51,54-55,58,61-62,65,68)^. Oito ECRs acompanharam os pacientes por 12 meses^(33-34,48,60,63-64,69-70)^, enquanto um estudo realizou o seguimento por 24 meses^(45)^ e outro por 26 meses^(41)^.

Em uma revisão sistemática prévia, observou-se que a melhoria na QVRS parece ser mais evidente a curto prazo em pacientes com DAC e em pacientes com IC ao longo de três meses^(81)^. Isso sugere a necessidade de novos estudos sobre a relação entre tempo de seguimento, população e recursos de telemedicina para auxiliar na identificação do melhor cuidado para cada paciente, considerando que a literatura ainda é incipiente nesse aspecto. Assim, o uso de metodologias mais robustas e aprimoradas, além da padronização dos protocolos de intervenções em telemedicina nas doenças cardíacas, devem ser incentivadas, uma vez que isso contribuirá para uma compreensão mais sólida dos reais benefícios e entraves da abordagem no contexto clínico dessas condições.

No que se refere às limitações dos estudos selecionados, observou-se uma predominância de questões amostrais, evidenciadas na análise crítica dos estudos. Apenas um estudo relatou o cegamento dos participantes^(63)^, dois estudos mencionaram o cegamento dos profissionais que realizaram as intervenções^(33,47)^ e 12 estudos indicaram que os avaliadores dos resultados estavam cegos para a alocação dos participantes^(32-33,36,40-41,47,50,52-55,64)^. Além disso, outras limitações foram citadas, tais como tamanho da amostra^(31-32,37-40,43-45,58,60,62,67,69,74)^, tempo de seguimento^(31,35,41,44,49,56)^ e viés de seleção ou recrutamento^(34-35,41,46-47,50-54,66-68,73)^, muitas vezes devido ao predomínio de uma doença cardiovascular específica, à presença majoritária da população masculina ou até mesmo relação ao local de recrutamento.

Como implicações para a prática, temos que o uso dos recursos da telemedicina pode ser considerado como ferramenta de suporte essencial para prover o cuidado integral ao indivíduo em ambientes intra e extra-hospitalares, considerando principalmente seus benefícios para diversos desfechos de saúde. Entretanto, ainda são necessários estudos robustos para melhor medir o efeito da estratégia da telemedicina e a influência nos desfechos de saúde, quanto ao tempo de acompanhamento, satisfação dos pacientes e dos profissionais e equidade do acesso, tendo em vista que as interações entre os desfechos são complexas e devem considerar os diversos contextos e populações.

No que se refere à avaliação da QVRS, é importante ressaltar a necessidade de estudos com abordagens multifacetadas considerando a complexidade da mensuração dessa variável, devido à sua natureza multidimensional, a fim de garantir interpretações mais precisas^(75-76)^. Além disso, a importância do aprofundamento das relações entre tempo de seguimento, população e intervenção de telemedicina também pode ser citada. Da mesma forma, políticas públicas eficazes e intervenções adaptadas às necessidades de cada comunidade^(9)^, com base em protocolos padronizados, são de extrema importância, uma vez que tem potencial para expandir o acesso à saúde, superando barreiras geográficas na promoção do cuidado^(15)^.

Por fim, entre as limitações desta revisão, destaca-se a não inclusão da literatura cinzenta, o que poderia ter ampliado a busca por evidências complementares. Além disso, a revisão enfrentou desafios relacionados à heterogeneidade das intervenções e dos recursos utilizados, bem como a especificidade das doenças cardíacas abordadas e dos desfechos avaliados. Essas variações dificultaram a realização de uma metanálise, impedindo uma síntese quantitativa robusta. No entanto, os resultados qualitativos permanecem válidos para avaliar a efetividade das intervenções nos contextos estudados.

## Conclusão

Em suma, a efetividade da telemedicina na QVRS de indivíduos com DCs ainda é inconclusiva. Embora a maioria dos estudos tenha demonstrado impacto positivo, muitos não alcançaram significância estatística. As principais intervenções utilizadas no cuidado e autogerenciamento dessas condições incluem telemonitoramento, contato por telefone e telerreabilitação. A telemedicina tem o potencial para ser uma ferramenta valiosa, comparável às intervenções presenciais em centros de saúde. No entanto, são necessários mais estudos que avaliem sua segurança, custo-efetividade e outros desfechos a longo prazo, especialmente o acompanhamento da QVRS para otimizar a implementação dessas tecnologias e garantir melhores resultados para os pacientes.

# Translation: Efecto de la telemedicina en la calidad de vida de personas con enfermedades cardíacas: una revisión sistemática

**DOI:** 10.1590/1518-8345.7243.4565

**Authors:** Gabriele Cardoso Gonçalves Alves, Fabiola Leticia Damascena Amador, Vagner Rogério dos Santos, Rita Simone Lopes Moreira

**Keywords:** Telemedicina, Cardiopatías, Automanejo, Calidad de Vida, Telerrehabilitación, Autocuidado

## Abstract

sintetizar la evidencia científica sobre la efectividad de las intervenciones basadas en telemedicina en la mejora de la calidad de vida relacionada con la salud y en el autogestión de pacientes con enfermedades cardíacas.

revisión sistemática de efectividad, siguiendo las recomendaciones del *Joanna Briggs Institute* y la guía de reporte *Preferred Reporting Items for Systematic Reviews and Meta-Analyses Checklist*. La búsqueda se realizó en seis bases de datos: *Cochrane Library*, Biblioteca Virtual en Salud, PubMed, CINAHL, *Web of Science Core Collection* y Scopus, sin restricción de período, en los idiomas inglés, portugués o español. La calidad metodológica y el riesgo de sesgo de los estudios fueron evaluados utilizando la herramienta de evaluación crítica de la JBI y la certeza de la evidencia fue clasificada mediante la herramienta GRADE.

de los 44 ensayos clínicos aleatorizados incluidos, las principales intervenciones analizadas fueron telemonitoreo, contacto telefónico y telerrehabilitación. De los estudios evaluados, el 88,63% demostró una mejora en la calidad de vida relacionada con la salud, con el 45,45% de estos presentando una diferencia estadísticamente significativa.

la telemedicina se muestra prometedora como una herramienta valiosa para el cuidado y autogestión de individuos con condiciones cardíacas. Sin embargo, se necesitan más estudios para confirmar su efectividad e impacto en los resultados de salud.

## Destacados

**(1)** Los principales recursos observados fueron: telemonitoreo, llamadas telefónicas y telerrehabilitación. **(2)** La telemedicina puede ser una herramienta efectiva en el cuidado de condiciones cardíacas. **(3)** El impacto real en la calidad de vida en las enfermedades cardíacas aún es incierto. **(4)** Se requieren más estudios relacionados con los aspectos de la calidad de vida.

## Introducción

Las enfermedades crónicas no transmisibles (ECNT) representan una parte significativa de las causas de muerte en el mundo, correspondiendo a cerca del 74% de los decesos a nivel global^(1)^. Entre ellas, las enfermedades cardiovasculares (ECV) asumen una posición alarmante, habiendo sido responsables de aproximadamente dos millones de muertes en la región de las Américas en el año 2019^(2)^.

Frente a este escenario, enfrentar el desafío de las ECV se convierte en una prioridad, especialmente debido al continuo envejecimiento de la población mundial y a la complejidad multifactorial que caracteriza estas enfermedades^(3)^. Además, las ECNT, con énfasis en las enfermedades cardíacas, se están convirtiendo en un marcador creciente de las desigualdades globales, siendo altamente incidentes en los países en desarrollo^(4)^.

Las enfermedades cardíacas son condiciones patológicas que involucran al corazón, en lo que respecta a su estructura y función^(5)^. El desarrollo, la progresión y el empeoramiento de estas condiciones están directamente relacionados con factores de riesgo, que pueden clasificarse como potencialmente modificables, como el control de la presión arterial, la glucemia y el perfil lipídico, o modificables, como la cesación del tabaquismo, la reducción del consumo excesivo de alcohol, el combate a la obesidad, el sedentarismo, entre otros^(6)^. Así, el uso de iniciativas educativas que busquen fomentar la literacidad en salud es recomendable, dado que estas acciones tienen el potencial de actuar sobre tales factores de riesgo, atenuando eventos adversos y readmisiones hospitalarias, al mismo tiempo que pueden incrementar la calidad de vida relacionada con la salud (CVRS) de los individuos afectados^(7)^.

En este contexto, se observa que las poblaciones con alto riesgo de ECV presentan resultados de CVRS más desfavorables^(7)^. De esta forma, esta variable emerge como un indicador crucial, siendo un fuerte predictor tanto para la mortalidad como para la hospitalización por insuficiencia cardíaca (IC), independientemente de la gravedad de los síntomas o de la fracción de eyección^(8)^.

Así, con el objetivo de enfrentar esta realidad, la telemedicina emerge como una estrategia prometedora para disminuir la mortalidad en individuos con problemas cardíacos y promover la CVRS y el autocuidado^(9)^. Además, como se mostró en una revisión sistemática sobre la eficacia, aceptabilidad y costos de la telemedicina realizada en 2015, al considerar estrategias para la mejora de factores de riesgo, hubo mejora de la CVRS, disminución de los niveles de hemoglobina glucosilada, lipoproteínas de baja densidad y presión arterial en individuos con ECNT^(10)^.

Es innegable que durante la pandemia de coronavirus hubo una amplia adopción de este formato de cuidado a distancia como una alternativa a las consultas presenciales, en respuesta a las restricciones al contacto directo, evidenciando la capacidad de adaptación del sistema frente a desafíos emergentes^(11)^.

La telemedicina se define por la prestación de servicios de salud de forma remota^(12)^, es decir, el paciente y el prestador están separados por la distancia, mediados por una herramienta tecnológica^(13)^. Existen diversos recursos en esta modalidad de cuidado en salud, como consultas con profesionales de la salud (teleconsulta, líneas directas y líneas de apoyo), telemonitoreo, telerrehabilitación, almacenamiento y envío de datos de salud (como imágenes, notas y vídeos) a los prestadores de cuidado y teleconsultoría entre profesionales de la salud que prestan el cuidado, comunicándose en busca de otras opiniones para el manejo de casos^(13-14)^.

Frente a esto, la telemedicina presenta un papel esencial en la asistencia a la salud global, siendo capaz de estructurar iniciativas terapéuticas que engloban objetivos educativos, diagnósticos precisos y monitoreo continuo^(15-16)^. Además, al superar limitaciones geográficas^(15)^, presenta el potencial de aumentar el acceso a los cuidados, reducir los costos y mejorar los resultados generales de salud^(16)^. De esta forma, existe la preocupación por parte de los profesionales de la salud en relación con la habilidad de los individuos para autogestionar sus propias condiciones en este formato de cuidado^(9)^. La autogestión es la “habilidad de gestionar síntomas, tratamiento, cambios físicos y psicosociales, así como cambios en el estilo de vida que los pacientes desarrollan al lidiar con enfermedades crónicas”^(17)^, generalmente necesitando el apoyo de un profesional de la salud^(18)^. En este contexto, se cree que la telemedicina tiene un impacto positivo en el apoyo al autogestión, mejorando consecuentemente la CVRS de pacientes con enfermedades cardíacas.

Hasta el momento, las revisiones sistemáticas previas que abordaron enfermedades cardíacas y telemedicina no se concentraron específicamente en el autogestión y en los cambios de la CVRS a lo largo del tiempo, abordando otros desenlaces como mortalidad, hospitalización y otros^(19-23)^. Además, los estudios relacionados con la CVRS percibida por los pacientes han mostrado divergencias en los resultados^(19-20,23-24)^ y, conforme se muestra en una revisión sistemática, se necesitan estudios que utilicen medidas estandarizadas para la evaluación de la CVRS, el autocuidado y la satisfacción^(10)^.

Frente a esto, esta revisión tuvo como objetivo sintetizar la evidencia científica sobre la efectividad de las intervenciones basadas en telemedicina en la mejora de la CVRS y en el autogestión de pacientes con enfermedades cardíacas.

## Método

Se trata de una revisión sistemática, conducida según las recomendaciones del *Joanna Briggs Institute* (JBI): *Evidence Synthesis Groups* ^(25)^ y reportada de acuerdo con las recomendaciones del *Preferred Reporting Items for Systematic Reviews and Meta-Analyses* PRISMA *Checklist* ^(26)^. El protocolo fue previamente publicado en el *Open Science Framework* ^(27)^ el 22 de enero de 2021, bajo el DOI 10.17605/OSF.IO/HQWGT. La búsqueda se realizó en marzo de 2023.

La investigación fue guiada por el acrónimo PICOS (P – *Population*; I – *Intervention*; C – *Comparison*; O - *Outcomes*; S - *Study*)^(25)^, siendo el P (individuos con edad mayor o igual a 18 años con enfermedades cardíacas), el I (recursos de telemedicina que apoyen el autogestión), C (cuidados habituales, basados en consultas ambulatorias), el O (CVRS) y S (ensayos clínicos controlados y aleatorizados). Con base en este acrónimo, se elaboró la siguiente pregunta orientadora: ¿Cuál es la efectividad de las intervenciones de telemedicina basadas en la autogestión en comparación con los cuidados habituales en la CVRS de pacientes adultos con enfermedades cardíacas?

## Criterios de elegibilidad

Se incluyeron ensayos clínicos aleatorizados (ECA) que compararon los cuidados habituales con el uso de la telemedicina en pacientes adultos (≥ 18 años) con enfermedades cardíacas, evaluando los resultados en la CVRS y el autogestión de estos individuos. Los criterios de exclusión fueron: ECA que no detallaron la metodología utilizada y artículos que incluían otras condiciones clínicas (por ejemplo, cáncer y diabetes).

## Fuentes de datos

Para la selección de los artículos, la estrategia de búsqueda fue implementada por el investigador principal en las siguientes bases de datos: *Cochrane Library* (*Wiley*), Biblioteca Virtual en Salud (Portal BVS), *Medical Literature Analysis and Retrieval System Online* (MEDLINE, PubMed), CINAHL (*EBSCOhost*), *Web of Science Core Collection* y *Scopus* (vía *Portal PeriódicosCAPES*).

## Estrategia de búsqueda

La estrategia de búsqueda fue específica para cada base de datos a partir del uso de dos vocabularios controlados en salud: *Medical Subject Headings* (MeSH) y Descriptores en Ciencias de la Salud (DeCS). Los descriptores utilizados fueron: “*Telemedicine*”, “*Heart Diseases*”, “*Self-Management*”, “*Cardiac Rehabilitation*” y “*Health Education*”, adecuados para cada base de datos. Las estrategias de búsqueda completas pueden consultarse en el Material Suplementario (https://doi.org/10.48331/scielodata.MI2JBD).

## Selección y extracción de datos

Dos revisores independientes seleccionaron los estudios, y un tercer evaluador resolvió las discrepancias. En primer lugar, se identificaron los títulos y resúmenes relacionados con la cuestión de investigación y el objetivo del estudio, y aquellos potencialmente elegibles fueron preseleccionados. En la segunda etapa, dos revisores independientes evaluaron los textos completos de los estudios preseleccionados para confirmar su elegibilidad. El proceso de selección se realizó mediante la plataforma *Rayyan QCRI* ^(28)^.

Los datos fueron extraídos de los estudios incluidos en la revisión por dos revisores independientes utilizando un formulario de extracción de datos predeterminado. El formulario incluyó los siguientes ejes: detalles metodológicos, intervención y resultados. El formulario puede consultarse en el Material Suplementario (https://doi.org/10.48331/scielodata.MI2JBD). Debido a la complejidad de las intervenciones, los estudios seleccionados fueron categorizados según la población estudiada y la intervención realizada, considerando los recursos utilizados. Los datos extraídos incluyeron detalles específicos sobre los participantes, métodos del estudio, intervenciones y resultados significativos para el objetivo de la revisión. Cualquier discrepancia surgida entre los revisores fue resuelta mediante discusión o con un tercer revisor. No fue necesario contactar a los autores de los artículos para obtener datos adicionales.

## Síntesis de los datos

Los hallazgos fueron presentados en forma narrativa. La síntesis narrativa incluyó una descripción detallada de los estudios incluidos, categorizados según la población estudiada, los cuestionarios utilizados para la medición de la CVRS, el tiempo de seguimiento y las intervenciones de telemedicina realizadas. Se utilizaron tablas y figuras para ayudar en la presentación de los datos.

## Evaluación crítica de los estudios

Dos revisores independientes realizaron una evaluación crítica de todos los estudios que cumplieron con los criterios de inclusión. Cualquier discrepancia entre los revisores fue resuelta por consenso o mediante la intervención de un tercer revisor. La calidad metodológica de los estudios fue verificada utilizando el *checklist* de evaluación crítica del JBI para ensayos clínicos aleatorizados. Este *checklist* está compuesto por 13 preguntas cuyas respuestas pueden ser “sí”, “no”, “incierto” o “no aplicable”^(29)^. Todos los estudios fueron clasificados en cinco dominios diferentes, resultando en clasificaciones de bajo riesgo de sesgo, riesgo de sesgo moderado o alto riesgo de sesgo. La certeza de la evidencia para el desenlace CVRS fue evaluada utilizando la herramienta *Grading of Recommendations Assessment, Development and Evaluation* (GRADE)^(30)^.

## Resultados

La búsqueda inicial en la literatura identificó un total de 2.341 estudios provenientes de bases de datos, además de cuatro estudios adicionales encontrados mediante búsqueda manual en listas de referencias de artículos incluidos. De estos, dos estudios fueron incluidos en la búsqueda primaria para el desarrollo de la estrategia de investigación, mientras que los otros dos fueron identificados tras el análisis de los protocolos de estudios encontrados en la búsqueda final.

Después de la eliminación de duplicados y la revisión de títulos y resúmenes, se seleccionaron 80 estudios para lectura completa. Las razones para la exclusión en esta etapa estuvieron relacionadas principalmente con criterios como población, intervención, comparación o desenlace. Los detalles sobre los motivos de exclusión pueden encontrarse en el Material Suplementario (https://doi.org/10.48331/scielodata.MI2JBD).

A partir de la lectura completa y el análisis de la calidad metodológica de los estudios, considerando los criterios de elegibilidad, se constituyó la muestra final que contó con 44 ensayos clínicos aleatorizados. La Figura 1 presenta el diagrama de flujo detallado del proceso de selección de los estudios incluidos en esta revisión sistemática.

**Figura 1 -:** Diagrama de flujo PRISMA^(26)^

La síntesis descriptiva de los ECA incluidos en esta revisión se presenta en la Figura 2 a continuación.

Los 44 ECRs incluidos en esta revisión involucraron un total de 12.732 pacientes, de los cuales 6.233 fueron asignados al grupo control y 6.499 al grupo de intervención. Las investigaciones se realizaron en cuatro continentes, con un 40,91% (n=18) de los estudios conducidos en Europa^(32,34-35,38-42,44,48,52,54,56-57,63-64,67,71)^, un 36,36% (n=16) en América del Norte^(37,43-46,51-53,59-60,62,65-67,63-64,66,68-70,72-74)^, un 13,64% (n=6) en Oceanía^(33,36,50,55,58,61)^ y un 9,09% (n=4) en Asia^(31,45,47,49)^. El período de seguimiento varió entre uno y 26 meses, con un promedio de seguimiento de 8,4 meses.

**Figura 2 -:** Descripción de las características de los estudios incluidos en la revisión sistemática (n = 44)

**Autor, año, país**	**Población, n, seguimiento**	**Intervención**	**Recursos**	**Comparación**	**Principales desenlaces**	**Resultados principales**
Choi, et al. ^( 31 )^ 2023 Corea	Individuos con diagnóstico de IC* n:76 (GC ^†^ :38| GI ^‡^ : 38) Seguimiento: 3 meses	Programa de autogestión vía aplicación móvil (“ *Heart Failure-Smart Life* ”)	Aplicación “ *Heart Failure-Smart Life* ”: Contenido educativo *Chat* individual con profesional de salud Registros de salud diarios para seguimiento personalizado e interactivo, con recursos adaptados a las necesidades individuales *Feedback* de autogestión por enfermera Aplicación distinta para los profesionales de la salud	Cuidados habituales: consultas con cardiólogo y enfermera cardiológica; breves informaciones sobre los medicamentos y la evolución de la enfermedad	Medidas antropométricas Clasificación funcional NYHA ^§^ Depresión ( *Geriatric* *Depression* *scale* ) CVRS ^||^ ( *MacNew Heart Disease Health-Related QoL* ) Adherencia a la medicación ( *Hill-Bone Medication Adherence* ) Autocuidado ( *European Heart Failure Self-Care Behaviour Scale* )	En la comparación entre grupos hubo mejoras significativas en la clase funcional NYHA ^§^ (p = 0,003) en el GI ^‡^ . La CVRS ^||^ no mostró diferencias significativas entre los grupos (GC ^†^ =5,34; GI ^‡^ =5,62). El comportamiento de autocuidado mejoró a lo largo del tiempo (p < 0,001), pero sin diferencias significativas entre los grupos.
Dalli Peydró, et al. ^( 32 )^ 2022 España	Individuos post-alta hospitalaria por SCA ^¶^ n: 59 (GC ^†^ : 28 | GI ^‡^ : 31) Seguimiento: 10 meses	Telerrehabilitación, precedida por entrenamiento hospitalario de 2 semanas; tras ajustes de ritmo y metas, la aplicación guió a los participantes a través de un programa diario de ejercicios	Aplicación: Programa diario de actividades físicas Registro del estado general Signos vitales Adherencia a la medicación Monitor de FC**	Rehabilitación cardíaca basada en centro	Aumento de la actividad física referida (Cuestionario Internacional de Actividad Física) VO _2_^††^ máx CVRS ^||^ (EQ-5D ^‡‡^ *5-levels* )	El GI ^‡^ presentó un mayor aumento en la actividad física (1.726 vs. 636 MET-min/semana, p = 0,045) y VO _2_^††^ máx (1,62 vs. 0,60 ml/kg/min, p < 0,004). La adherencia a la dieta mediterránea, el malestar psicológico y la CVRS ^||^ mostraron mayor mejora en el GI ^‡^ que en el GC ^†^ . La autoevaluación de la salud mejoró en ambos grupos, pero solo fue significativa en el GI ^‡^ (p = 0,008).
Chow, et al. ^( 33 )^ 2022 Australia	Individuos con diagnóstico de SCA ^¶^ n:1424 (GC ^†^ :708| GI ^‡^ :716) Seguimiento: 12 meses	Programa de SMS ^§§^ personalizadas destinado a mejorar la adherencia a medicamentos y la prevención secundaria	Envío de SMS ^§§^ motivacionales y educativos divididos en tres módulos principales: Estilo de vida (dieta, ejercicio, cesación del tabaco), Medicamentos (información sobre uso y efectos secundarios), Prevención secundaria general (metas de salud, apoyo mental y servicios de salud) Consejero de salud	Los cuidados habituales no fueron descritos	Adherencia a la medicación autorreferida Medidas antropométricas y de laboratorio CVRS ^||^ (SF ^||||^ -12)	No hubo diferencias significativas en la adherencia a la medicación autorreferida entre el GI ^‡^ y el GC ^†^ (0,93 [IC 95%, 0,84–1,03]; p=0,15). No hubo diferencias en las puntuaciones de depresión y ansiedad, pero se observó una ligera mejora en la puntuación de CVRS ^||^ (componente físico) para el GI ^‡^ (diferencia media [IC 95%], 1,1 [0,0, 2,2]; p=0,045).
Voller, et al. ^( 34 )^ 2022 Alemania	Individuos con diagnóstico de IC* n:621 (GC ^†^ :319 |GI ^‡^ :302) Seguimiento: 12 meses	Telemonitoreo domiciliario vía sistema Motiva ^®^ , que capacita al paciente para gestionar su condición de salud y facilita el contacto diario entre médicos y pacientes	Sistema Motiva ^®^ Registro regular de parámetros vitales (PA ^¶¶^ , FC** y peso) Material educativo, material de entrenamiento, cuestionarios, recordatorios y retroalimentación sobre el estado de salud vía Motiva ^®^ Seguimiento telefónico de posibles descompensaciones	Cuidado habitual, basado en las directrices de la Sociedad Europea de Cardiología	Costo-efectividad incremental Mortalidad CVRS ^||^ (SF ^||||^ -36 V2; WHO-5***; KCCQ ^†††^ ) Capacidad funcional	La intervención no tuvo impacto en el riesgo de mortalidad. Todas las escalas de CVRS ^||^ mejoraron de forma consistente y significativa en el GI ^‡^ a los 12 meses en comparación con el GC ^†^ (todos p < 0,01).
Brouwers, et al. ^( 35 )^ 2021 Países Bajos	Individuos en rehabilitación (fase 2) para EAC ^‡‡‡^ n: 300 GC ^†^ : 147 | GI ^‡^ : 153) Seguimiento: 12 meses	Telerrehabilitación, que incluyó 6 sesiones de ejercicios en grupo, consultas semanales por vídeo realizadas por fisioterapeutas hasta la consecución de las metas individuales o finalización del programa, además del telemonitoreo semanal	Aplicación: Monitoreo de signos vitales Monitor de FC** Acelerómetro Módulo de entrenamiento físico Videoconsultas semanales	Rehabilitación cardíaca basada en centro	CVRS ^||^ (EQ-5D ^‡‡^ *5-levels* ; EQ-VAS ^§§§^ ; *MacNew Heart Disease Health-Related Quality of Life Questionnaire* ) Costos en salud	Los pacientes del GI ^‡^ y del GC ^†^ presentaron CVRS ^||^ comparable (diferencia media en EQ-5D-5L ^‡‡^ : −0,004; p = 0,82; diferencia media en EQ-VAS ^§§§^ : −0,001). Aunque los costos de intervención fueron más altos, no hubo diferencias en los costos totales de salud cardíaca entre los dos grupos (€4.787 vs. €5.507, p = 0,36).
Maddison, et al. ^( 36 )^ 2021 Nueva Zelanda	Individuos adultos con SCA ^¶^ n:306 (GC ^†^ :153| GI ^‡^ :153) Seguimiento: 13 meses	Programa de SMS ^§§^ automatizadas denominado *Text4Heart II* , promoviendo el autogestión y la educación en salud	Envío de SMS ^§§^ personalizados Llamadas telefónicas para aplicación de cuestionarios	Cuidados habituales ambulatorios, sin detalles específicos	Adherencia a la medicación ( *Morisky Medication Adherence Scale* ) CVRS ^||^ (EQ-5D ^‡‡^ )	La adherencia a la medicación en tres clases fue menor en el GI ^‡^ en comparación con el GC ^†^ , tanto a las 24 semanas (56,8% vs 68,6%) como a las 52 semanas (67,9% vs 54,2%). Las puntuaciones de autorreporte de adherencia a la medicación reflejaron esta tendencia a las 52 semanas.
Collins, et al. ^( 37 )^ 2021 Estados Unidos de América	Individuos con diagnóstico de IC* n:491 (GC ^†^ :245 | GI ^‡^ :246) Seguimiento: 12 meses	Cuidados habituales y plan de alta personalizado, *coaching* telefónico de autocuidado, con intervención compuesta por visita domiciliaria dentro de los 7 días posteriores al alta y entrenamiento de autocuidado por teléfono	Llamadas telefónicas Consulta por telesalud	Cuidados habituales compuestos por proceso de alta estructurado, incluyendo reconciliación y prescripción de medicación, y consulta médica de seguimiento	Muerte asociada a enfermedad cardiovascular Eventos relacionados con IC* CVRS ^||^ (KCCQ ^†††^ )	No hubo diferencia significativa entre los grupos en cuanto a mortalidad, eventos relacionados con la IC* y cambios en la puntuación resumida del KCCQ ^†††^ a los 90 días. Aunque la puntuación del KCCQ ^†††^ fue mayor en el GI ^‡^ , la diferencia no fue estadísticamente significativa (IC 95% -1,9 a 7,2; p = 0,25).
Clays, et al. ^( 38 )^ 2021 Bélgica e Italia	Individuos con diagnóstico de IC* n:56 (GC ^†^ :22 | GI ^‡^ :34) Seguimiento: 6 meses	Sistema de telemonitoreo domiciliario que consistía en una combinación de dispositivos de monitoreo y una aplicación móvil, destinados a brindar soporte integral para la gestión de la salud de los pacientes	Sistema *HeartMan* : Monitor vestible de FC** y PA ^¶¶^ Balanza Organizador de comprimidos *Smartphone* con la aplicación móvil *HeartMan* , con recursos para manejo físico, soporte psicológico y educación sobre IC* Servicio de asistencia telefónica operativa	Cuidados habituales con tratamiento estándar según las guías clínicas ofrecidas por el cardiólogo, médico general y enfermera de IC*	CVRS ^||^ (MLHFQ ^||||||^ ) Capacidad de ejercicio (TC6M ^¶¶¶^ ) Percepción de enfermedad y salud mental ( *Brief Illness Perception Questionnaire* ) Autocuidado ( *Self-Care of Heart Failure Index* )	En el GI ^‡^ , hubo reducción significativa de la depresión y ansiedad (p < 0,001), mientras que en el GC ^†^ disminuyó la necesidad de asesoramiento sexual (p < 0,05). Solo en el GI ^‡^ , el autocuidado aumentó (p < 0,05) y los problemas sexuales disminuyeron (p < 0,05); sin embargo, no hubo impacto significativo en la CVRS ^||^ , confianza en el autocuidado, percepción de la enfermedad o capacidad de ejercicio entre los grupos.
Batalik, et al. ^( 39 )^ 2020 República Checa	Individuos con enfermedad cardiovascular sometidos a revascularización cardíaca n: 56 (GC ^†^ : 28 | GI ^‡^ : 28) Seguimiento: 3 meses	Telerrehabilitación, precedida por entrenamiento supervisado en ambulatorio	Aplicación: Monitoreo de signos vitales Monitor de FC ^**^ Módulo de entrenamiento físico Reevaluación telefónica semanal	Rehabilitación cardíaca basada en centro	Aptitud física (Prueba Cardiopulmonar de Ejercicio) CVRS ^||^ (SF ^||||^ -36) Adherencia al entrenamiento	Ambos grupos mostraron mejoras significativas en la aptitud física (p < 0,001), con adherencia similar entre ellos. La CVRS ^||^ mejoró significativamente en ambos grupos, sin diferencias significativas entre ellos (p < 0,01).
Lunde, et al. ^( 40 )^ 2020 Noruega	Individuos en rehabilitación(fase 2) para EAC ^‡‡‡^ n: 300 GC ^†^ : 147 |GI ^‡^ :153) Seguimiento: 12 meses	Seguimiento individualizado vía aplicación, con definición de metas, recordatorios, evaluación de progreso y *feedback* personalizado	Aplicación: Orientaciones conductuales (actividad física y dieta) Hábitos saludables Definición de metas individuales Recordatorios *Feedback* individual	Cuidados habituales no detallados	Diferencia en VO _2_^††^ pico Desempeño en el ejercicio Peso corporal PA ^¶¶^ en reposo Hábitos de ejercicio CVRS ^||^ ( *HeartQoL* ^****^ ) Estado de salud (EQ-5D ^‡‡^ 5 *levels* )	En el GI ^‡^ , hubo diferencia significativa en el VO _2_^††^ pico en comparación con el GC ^†^ (diferencia media de 2,2 ml/kg/min, IC 95% 0,9–3,5, p = 0,001). Además, el GI ^‡^ presentó mejor desempeño en el ejercicio, hábitos de ejercicio y autopercepción del cumplimiento de metas. No se encontraron diferencias significativas en la PA ^¶¶^ , CVRS ^||^ ni estado de salud entre los grupos.
Piotrowicz, et al. ^( 41 )^ 2020 Polonia	Individuos con diagnóstico de IC* n:850 (GC ^†^ :425|GI ^‡^ :425) Seguimiento: 14 a 26 meses	Telerrehabilitación iniciada con entrenamiento hospitalario, seguida de entrenamiento domiciliario. Incluyó supervisión médica, educación y planificación de ejercicios personalizados para cada paciente.	Centro de monitoreo con disponibilidad de teléfono celular para: Registro de signos vitales y datos clínicos Entrenamiento físico individualizado Programa educativo ECG ^††††^ portátil Monitor de PA ^¶¶^ Báscula Monitor de cardiodesfibrilador (solo para pacientes con implante)	Los cuidados habituales incluyeron evaluaciones clínicas regulares, participación en programas de rehabilitación, monitoreo remoto de dispositivos cardíacos implantables y asesoramiento sobre cambios en el estilo de vida y autogestión según las directrices clínicas estándar.	Porcentaje de supervivencia fuera del hospital Mortalidad Cambio en la duración de la prueba de ejercicio cardiopulmonar CVRS ^||^ (SF ^||||^ -36)	No hubo diferencias significativas en la tasa de mortalidad (12,5% vs 12,4%, respectivamente; tasa de riesgo, 1,03 [Intervalo de confianza 95%, 0,70-1,51]) ni en hospitalización (media [DE] de días: 91,9 [19,3] vs 92,8 [18,3], respectivamente; P = 0,74) entre los grupos. Sin embargo, el GI ^‡^ presentó beneficios significativos en términos de mejora del consumo máximo de oxígeno y de la CVRS ^||^ (puntuación del SF ^||||^ -36, 1,58 [Intervalo de confianza 95%, 0,74-2,42] vs 0,00 [Intervalo de confianza 95%, -0,84 a 0,84]; P = 0,008), siendo bien tolerada sin eventos adversos graves durante el ejercicio.
Ávila, et al. ^( 42 )^ 2020 Bélgica	Individuos con EAC ^‡‡‡^ n:90 (GC ^†^ cuidados habituales:30| GC ^†^ rehabilitación:30 | GI ^‡^ : 30) Seguimiento: 3 meses	Programa de telerrehabilitación asociado a telemonitoreo y prescripción de ejercicio individualizada	Aplicación Dispositivo vestible (reloj) *Feedback* por teléfono o correo electrónico	Rehabilitación basada en centro con entrenamiento ambulatorio Cuidados habituales: incentivados a mantener un estilo de vida físicamente activo y consultas de seguimiento	Aptitud cardiorrespiratoria (VO _2_^††^ pico) Actividad física Factores de riesgo cardiovasculares tradicionales CVRS ^||^ (SF ^||||^ -36)	Todos los grupos mantuvieron puntuaciones elevadas en todos los parámetros de CVRS ^||^ , sin diferencias significativas entre los grupos (p = 0,70). La capacidad de ejercicio y los resultados secundarios se preservaron en los tres grupos a lo largo del tiempo (p > 0,05 para todos), independientemente de la intervención.
Cáceres, et al. ^( 43 )^ 2020 Estados Unidos de América	Individuos con diagnóstico de FA ^‡‡‡‡^ o *Flutter* Atrial n:238 (GC ^†^ :123|GI ^‡^ :115) Seguimiento: 6 meses	Intervención *iHEART* de telemonitoreo y orientaciones vía SMS ^§§^	Uso de *iPhone* con sistema de ECG ^††††^ móvil *AliveCor* ^®^ *Kardia* para monitoreo cardíaco remoto Envío de SMS ^§§^ con orientaciones sobre manejo de la FA ^‡‡‡‡^ y estilo de vida	Cuidados habituales con tratamiento estándar según las guías vigentes	Recurrencia de FA ^‡‡‡‡^ CVRS ^||^ (SF ^||||^ -36; EQ-5D-5L ^‡‡^ ; *Atrial Fibrillation Effect on Quality of Life* ) Gravedad de los síntomas de FA ^‡‡‡‡^ ( *Atrial Fibrillation Severity Scale* )	Hubo mejora en ambos grupos en las puntuaciones desde el inicio hasta el seguimiento para las escalas de efecto de la FA ^‡‡‡‡^ en la CVRS ^||^ (p < 0,05), con mayor impacto en el GI ^‡^ . No hubo diferencias estadísticamente significativas en la CVRS ^||^ ni en la gravedad de los síntomas de FA ^‡‡‡‡^ entre los grupos.
Cichosz, et al. ^( 44 )^ 2019 Dinamarca	Individuos con diagnóstico de IC* n:299 (GC ^†^ :154|GI ^‡^ :145) Seguimiento: 12 meses	Intervención *Telekits* , con sistema clínico central que incluía monitoreo de signos vitales, evaluación de enfermería, contactos telefónicos para manejo y derivación a consultas médicas si fuera necesario	*Tablets* Monitores digitales de PA ^¶¶^ Balanza Contacto telefónico	Rehabilitación, asesoramiento dietético, entrenamiento, monitoreo de medicamentos, evaluación de factores de riesgo y orientación sobre cambios en el estilo de vida	CVRS ^||^ (SF ^||||^ -36): componente físico y mental Puntuación del cuestionario específico de IC* (KCCQ ^†††^ )	El GI ^‡^ presentó una mejora significativa en las puntuaciones mentales en comparación con el GC ^†^ (p < 0,01). No hubo efecto significativo en la mejora de la puntuación física (SF ^||||^ -36) ni en la calidad de vida relacionada con la IC* (KCCQ ^†††^ ).
Mizukawa, et al. ^( 45 )^ 2019 Japón	Individuos con diagnóstico de IC* n: 59 (GC ^†^ :19| GI ^‡1^ :20| GI ^‡ 2^ : 20) GI ^‡1^ : Gestión colaborativa GI ^‡2^ : Educación para autogestión Seguimiento: 24 meses	Sistema de monitoreo y autogestión, con registro diario de signos vitales, visitas al médico, sesiones mensuales de educación en autogestión y monitoreo remoto telefónico por enfermera para el GI ^‡1^	Monitor de FC** Monitor de PA ^¶¶^ Monitoreo de peso Intervención educativa Gestión telefónica por enfermera, solo para GI ^‡1^	Visitas al médico cada 2-4 semanas, sesión educativa al alta y orientación para el registro diario de peso, PA ^¶¶^ y pulso en un cuaderno de autogestión	CVRS ^||^ (MLHFQ ^||||||^ ) Autoeficacia ( *Chronic Disease Self-Efficacy Scale* ) Autocuidado ( *European Heart Failure Self-Care Behaviour Scale* ) Rehospitalización por IC* Mortalidad por todas las causas	La CVRS ^||^ mejoró significativamente en el grupo GI ^‡1^ en comparación con el GC ^†^ a los 18 y 24 meses (p < 0,05). El GI ^‡1^ también mostró mejoras significativas en autoeficacia y autocuidado (p < 0,01) y tuvo menores tasas de rehospitalización (20,0% vs 57,9% en el GC ^†^ ), con mayor supervivencia sin readmisión (p = 0,020).
Pekmezaris, et al. ^( 46 )^ 2019 Estados Unidos de América	Individuos afroamericanos e hispanos de comunidades desfavorecidas con diagnóstico primario de IC* n: 104 (GC ^†^ :58 | GI ^‡^ :46) Seguimiento: 3 meses	Automonitoreo vía telesalud, centrado en la autogestión diaria y visitas semanales de telesalud con apoyo intensivo y continuo por parte de una enfermera	Monitor de FC** Monitor de PA ^¶¶^ Monitoreo de peso Monitor de saturación de oxígeno Videoconsulta semanal con enfermera	Manejo ambulatorio integral, basado en los cuidados habituales en un entorno ambulatorio, con adherencia a las directrices de la *American Heart Association*	Hospitalización y uso de servicios de emergencia CVRS ^||^ (MLHFQ ^||||||^ ) Ansiedad y depresión ( *Patient Health Questionnaire -4* )	No hubo diferencias significativas entre el GI ^‡^ y el GC ^†^ en visitas a urgencias (Riesgo Relativo = 1,37, IC = 0,83–2,27), hospitalizaciones (Riesgo Relativo = 0,92, IC = 0,57–1,48) o tiempo de hospitalización (GI ^‡^ = 0,54 vs. GC ^†^ = 0,91). Ambos grupos mostraron mejoras en la CVRS ^||^ , sin diferencias estadísticas.
Peng, et al. ^( 47 )^ 2018 China	Individuos con diagnóstico de IC* y sus cuidadores n:98 (GC ^†^ :49 | GI ^‡^ :49) Seguimiento: 4 meses	Telerrehabilitación, programa de entrenamiento físico vía telesalud con sesiones de ejercicios monitorizadas por fisioterapeutas y seguimiento regular de enfermeros cardíacos por teléfono o SMS ^§§^	Programa de entrenamiento físico por telesalud Folleto educativo Sesiones de ejercicios supervisadas por video Seguimiento por enfermeras vía llamadas telefónicas o mensajes instantáneos ( *Wechat* )	Cuidados habituales, basados en una sesión educativa al alta hospitalaria y visitas regulares de seguimiento en la clínica. Los pacientes de este grupo no recibieron instrucciones específicas sobre ejercicios físicos.	CVRS ^||^ (MLHFQ ^||||||^ ) Distancia recorrida en TC6M ^¶¶¶^ Medidas fisiológicas Ansiedad y depresión ( *Hospital Anxiety and Depression Scale* )	El GI ^‡^ demostró mejoras estadísticamente significativas en la CVRS ^||^ (Fb = 8,272, p = 0,005), sostenidas hasta 4 meses después del post-test (Fin = 79,73, p = 0,000) y en la distancia recorrida en el TC6M ^¶¶¶^ en comparación con el GC ^†^ . No se observaron mejoras significativas en otros parámetros.
Wagenaar, et al. ^( 48 )^ 2018 Países Bajos	Individuos con diagnóstico de IC* n:450 (GC ^†^ :150| GI ^‡^ site:150 | GI ^‡^ e-Vita:150) Seguimiento: 12 meses	La intervención consistió en un sitio *web* con contenido educativo en salud. Además, los participantes del GI ^‡^ e-Vita siguieron un plan de cuidados en e-salud con telemonitoreo y ajustes personalizados.	Sitio *web* Orientaciones de enfermería Plataforma e-Vita Registro regular de parámetros vitales (peso, PA ^¶¶^ y FC**) Alertas en caso de valores fuera de los límites preestablecidos Actualizaciones sobre comorbilidades y medicamentos Recordatorios mensuales por correo electrónico para seguimiento y adherencia al plan de cuidados	Cuidados habituales de nueve equipos ambulatorios de IC*, incluyendo consultas de rutina con un cardiólogo y una enfermera de IC*, en promedio, cuatro veces al año.	Autocuidado del paciente ( *European Heart Failure Self-Care Behavior Scale* ) CVRS ^||^ (MLHFQ ^||||||^ ) Conocimiento específico de la enfermedad ( *Dutch Heart Failure Knowledge Scale* ) Satisfacción del paciente con los cuidados de IC* Mortalidad	Después de 3 meses, los GI ^‡^ mostraron mejor autocuidado en comparación con el GC ^†^ (GI ^‡^ sitio 73,5 vs. 70,8 y GI ^‡^ e-Vita 78,2 vs. 70,8). Además, después de 3 y 6 meses, hubo diferencias significativas en la CVRS ^||^ entre el GI ^‡^ e-Vita y el GC ^†^ (mediana GI ^‡^ e-Vita 19,0 vs. GC ^†^ 22,8, p = 0,029 y GI ^‡^ e-Vita 21,0 vs. GC ^†^ 24,0, p = 0,003). Estas diferencias disminuyeron con el tiempo, sin diferencias significativas después de un año.
Guo, et al. ^( 49 )^ 2017 China	Individuos con FA ^‡‡‡‡^ n: 209 (GC ^†^ :96 | GI ^‡^ :113) Seguimiento: 3 meses	Sistema de autogestión basado en una aplicación móvil, que ofrece soporte para la toma de decisiones clínicas, programas educativos, monitoreo de salud y seguimiento estructurado	Aplicación de soporte para la toma de decisiones clínicas: Cálculo automático de puntuaciones de riesgo Programas educativos Componentes de involucramiento en el autocuidado Componentes de seguimiento estructurado Registro personal de salud	Los cuidados habituales no fueron descritos	Conocimiento de los pacientes sobre FA ^‡‡‡‡^ CVRS ^||^ (EQ-5D ^‡‡^ ) Adherencia a la medicación ( *Pharmacy Quality Alliance* ) Usabilidad, viabilidad y aceptabilidad de la aplicación	Más del 90% de los pacientes informaron que la aplicación era fácil de usar y útil, asociada a mejoras significativas en el conocimiento (p < 0,05). La adherencia a los medicamentos y la satisfacción con los anticoagulantes fueron significativamente mejores en el GI ^‡^ en comparación con el GC ^†^ (p < 0,05). Las puntuaciones de CVRS ^||^ aumentaron significativamente con la aplicación, con reducción de la ansiedad y la depresión (p < 0,05).
Hwang, et al. ^( 50 )^ 2017 Australia	Individuos con diagnóstico de IC* n:53 (GC ^†^ :29 | GI ^‡^ :24) Seguimiento: 6 meses	Telerehabilitación con un programa realizado dos veces por semana, videollamadas, supervisión de un fisioterapeuta, ejercicios en tiempo real, sesiones educativas y apoyo remoto	Plataforma de videollamadas para telerehabilitación Interacción entre el fisioterapeuta y los participantes, con *feedback* y modificaciones en los planes terapéuticos Presentaciones educativas con archivos de audio integrados Discusiones grupales en línea Diseño colaborativo Funciones de *chat* Manual de equipos con instrucciones escritas e ilustradas	Rehabilitación tradicional basada en centro	CVRS ^||^ (MLHFQ ^||||||^ ; EQ-5D ^‡‡^ ) Distancia recorrida en el TC6M ^¶¶¶^ Satisfacción ( *Client Satisfaction Questionnaire* ) Tasa de eventos adversos	No se encontraron diferencias significativas entre los grupos en relación con la CVRS ^||^ , indicando resultados similares. Ambos grupos mostraron mejoras estadísticamente significativas en la CVRS ^||^ , mantenidas durante el seguimiento. En cuanto a la distancia recorrida en el TC6M ^¶¶¶^ , no se observaron diferencias significativas entre los grupos, con una diferencia media de 15 metros (IC 95% –28 a 59).
Jayaram, et al. ^( 51 )^ 2017 Estados Unidos de América	Individuos con diagnóstico de IC* n:1521 (GC ^†^ :765 | GI ^‡^ :756) Seguimiento: 6 meses	Programa Tele-HF de telemonitoreo basado en llamadas telefónicas	Materiales educativos Balanza Llamadas diarias con respuestas a cuestionarios *Feedback* médico cuando fuera necesario Recordatorios	Cuidados habituales no detallados, referidos como recomendaciones estándar de las guías para el tratamiento de la IC*, con materiales educativos y balanza cuando fuera necesario	CVRS ^||^ (KCCQ ^†††^ )	Durante el seguimiento de 6 meses, los pacientes del GI ^‡^ presentaron una puntuación media del KCCQ ^†††^ 2,5 puntos mayor (IC 95% = 0,38–4,67; p = 0,02) que aquellos con cuidados habituales. Esta diferencia se debió principalmente a mejoras en los síntomas (3,5 puntos; IC 95% = 1,18–5,82; p = 0,003) y la función social (3,1 puntos; IC 95% = 0,30–6,00; p = 0,03).
Piotrowicz, et al. ^( 52 )^ 2020 Polonia	Individuos con diagnóstico de IC* n:111 (GC ^†^ :34| GI ^‡^ : 77) Seguimiento: 2 meses	Programa de telerehabilitación asociado a telemonitoreo y prescripción de ejercicio individualizado, basado en marcha nórdica	Equipo de telerrehabilitación y monitoreo remoto: Mini dispositivo EHO Monitor de PA ^¶¶^ Balanza ECG ^††††^ Teléfono móvil Cuestionarios Soporte telefónico (apoyo psicológico e instrucciones adicionales)	Cuidados habituales sin prescripción formal de entrenamiento físico o rehabilitación supervisada. Recomendaciones sobre cambios de estilo de vida y autogestión según las directrices de la Sociedad Europea de Cardiología	Capacidad funcional (VO _2_^††^ pico) CVRS ^||^ (SF ^||||^ -36) Evaluación ampliada de la eficacia de la rehabilitación Seguridad y adherencia al entrenamiento	El GI ^‡^ mostró una mejora significativa en la capacidad funcional (p = 0,0001) con repercusiones positivas en la CVRS ^||^ , aunque sin diferencias estadísticas significativas. Todos los participantes completaron el programa de rehabilitación, con una alta adherencia al entrenamiento domiciliario de marcha nórdica (94,7% de adherencia).
Bekelman, et al. ^( 53 )^ 2015 Estados Unidos de América	Individuos con diagnóstico de IC* n:392 (GC ^†^ :193| GI ^‡^ :199) Seguimiento: 12 meses	El Programa *Patient-Centered Disease Management* que combinó cuidados colaborativos multidisciplinarios, telemonitoreo y manejo conjunto de IC* y depresión	Cuidados colaborativos multidisciplinarios Cribado y tratamiento de la depresión Telemonitoreo y apoyo al autocuidado del paciente: Monitor de PA ^¶¶^ y FC** Monitoreo de peso y síntomas autorreportados Recordatorios de medicación Material educativo sobre IC* y depresión Asesoramiento dietético	Seguimiento con el equipo de salud de cuidados habituales, información en la visita de inscripción que describía el autocuidado para IC*, y una balanza si era necesario. Los médicos eran notificados si el paciente presentaba síntomas depresivos.	CVRS ^||^ (KCCQ ^†††^ ) Síntomas depresivos (PHQ-9 ^§§§§^ ) Hospitalizaciones Mortalidad	No hubo diferencias significativas en las características basales entre los grupos de intervención y de cuidados habituales. Después de un año, ambos grupos mostraron mejoras similares en las puntuaciones del KCCQ ^†††^ (cambio medio de 13,5 puntos, p = 0,97). Sin embargo, la intervención se asoció a una menor mortalidad en un año (4,3% vs 9,6%, p = 0,04).
Frederix, et al. ^( 54 )^ 2015 Bélgica	Individuos con EAC ^‡‡‡^ en rehabilitación cardíaca o IC* n: 140 (GC ^†^ : 70| GI ^‡^ :70) Seguimiento: 6 meses	Telerrehabilitación compuesta por telemonitoreo y *tele-coaching* , añadido al programa convencional de rehabilitación cardíaca de 12 semanas basado en centro	Telemonitoreo de la actividad física Acelerómetro *Tele-coaching* vía sitio *web* Orientaciones sobre cesación del tabaquismo, dieta y actividad física *Feedback* por correo electrónico o SMS ^§§^	Rehabilitación tradicional basada en centro	Capacidad aeróbica máxima (VO _2_^††^ pico) Actividad física diaria Hemoglobina glicosilada, control glucémico y perfil lipídico CVRS ^||^ (HeartQoL****)	Los pacientes del GI ^‡^ mostraron un aumento significativo en el VO _2_^††^ pico desde la línea base (media de 22,46) hasta las 24 semanas (media de 24,46, p < 0,01), mientras que en el GC ^†^ no hubo cambios significativos. Además, la actividad física autorreportada y la puntuación global de CVRS ^||^ mejoraron más en el GI ^‡^ en comparación con el GC ^†^ a las 24 semanas (p = 0,01).
Maddison, et al. ^( 55 )^ 2015 Nueva Zelanda	Individuos con enfermedad cardíaca isquémica n: 171 (GC ^†^ :86 | GI ^‡^ :85) Seguimiento: 6 meses	Programa *HEART* , que consistió en la prescripción de ejercicios y apoyo conductual mediante SMS ^§§^ e internet, con el objetivo de aumentar la actividad física en los pacientes	Prescripción de ejercicios regulares Estrategias para el cambio de comportamiento Sitio *web* con información sobre la enfermedad y opción de automonitoreo del progreso Envío diario de SMS ^§§^	Los cuidados habituales no fueron descritos	Capacidad aeróbica máxima (VO _2_^††^ pico) Actividad física autorreportada (Cuestionario Internacional de Actividad Física) Autoeficacia y motivación para el ejercicio CVRS ^||^ (SF ^||||^ -36 versión 2; EQ-5D ^‡‡^ )	Hubo un aumento significativo en la actividad física recreativa autorreportada (p = 0,05) y en caminatas (p = 0,02) en el GI ^‡^ . También se observaron mejoras significativas en la autoeficacia para mantenerse activo y en el dominio de salud general del SF ^||||^ -36 en el GI ^‡^ , con una diferencia de 2,1 puntos (IC 95%: 0,1-4,1; p = 0,03) a las 24 semanas para la CVRS ^||^ .
Piotrowicz, et al. ^( 56 )^ 2015 Polonia	Individuos con diagnóstico de IC* n: 152 (GC ^†^ :75 | GI ^‡^ :77) Seguimiento: 2 meses	Telerrehabilitación basada en caminatas, utilizando un sistema de telemonitoreo y entrenamiento supervisado	Telerrehabilitación con sesiones de entrenamiento preprogramadas para cada individuo Telemonitoreo Envío de ECG ^††††^ en reposo y respuesta de un cuestionario de salud antes de la actividad física Apoyo psicológico	Rehabilitación tradicional basada en centro	CVRS ^||^ (SF ^||||^ -36)	Se observó una mejora en la CVRS ^||^ total en ambos grupos, pero no fue estadísticamente significativa. El GI ^‡^ mostró una mejora principalmente en las categorías mentales, mientras que el GC ^†^ mejoró su bienestar físico general.
Kraal, et al. ^( 57 )^ 2014 Países Bajos	Individuos con EAC ^‡‡‡^ que ingresaron en la rehabilitación cardíaca tras hospitalización por IAM ^||||||||^ , angina inestable o revascularización (Riesgo bajo a moderado) n: 55 (GC ^†^ :26 | GI ^‡^ :29) Seguimiento: 3 meses	Telerrehabilitación, que comenzaba con sesiones de entrenamiento monitorizado, establecimiento de metas y entrevistas motivacionales, *feedback* telefónico del fisioterapeuta	Telerrehabilitación Telemonitoreo con monitor vestible de FC** Aplicación Retroalimentación telefónica	Rehabilitación tradicional basada en centro	VO _2_^††^ pico CVRS ^||^ ( *MacNew Heart Disease Health-Related QoL* ) Adherencia al entrenamiento	Se observó una mejora significativa en el consumo de VO _2_^††^ pico en ambos grupos (GC ^†^ : 10% y GI ^‡^ : 14%), sin diferencias significativas entre los grupos. La CVRS ^||^ mejoró significativamente en ambos grupos, sin diferencias entre ellos.
Varnfield, et al. ^( 58 )^ 2014 Australia	Individuos post IAM ^||||||||^ n: 120 (GC ^†^ : 60| GI ^‡^ :60) Seguimiento: 6 meses	Telerrehabilitación mediante la Plataforma de Evaluación de Cuidados para Rehabilitación Cardíaca (CAP-CR), basada en la prescripción de ejercicios y el envío de contenido educativo	Plataforma CAP-CR: Monitoreo de salud Monitoreo de ejercicios SMS ^§§^ y archivos de audio con contenido motivacional y educativo Portal *web* Consultas semanales con mentores	Rehabilitación tradicional basada en centro	Adherencia, cumplimiento y finalización del programa de rehabilitación cardíaca Factores de riesgo biomédicos (PA ^¶¶^ , FC**, peso, entre otros) CVRS ^||^ (EQ-5D ^‡‡^ )	El GI ^‡^ tuvo tasas significativamente más altas de adherencia (80% vs 62%) y finalización (80% vs 47%) del programa en comparación con el GC ^†^ (p < 0,05). Ambos grupos mejoraron significativamente en el TC6M ^¶¶¶^ , manteniéndose a los 6 meses. El GI ^‡^ también mostró mejoras en la CVRS ^||^ (EQ-5D ^‡‡^ : mediana de 0,84 a 0,92).
Blum et al. ^( 59 )^ 2014 Estados Unidos de América	Individuos con diagnóstico de IC* n: 206 (GC ^†^ : 102|GI ^‡^ :104) Seguimiento: 12 meses	Sistema de telemonitoreo de signos vitales, con monitoreo de enfermería y contactos con el paciente	Sistema *E-care de Philips Electronics* para el monitoreo remoto de datos Balanza Monitor de PA ^¶¶^ ECG ^††††^	Acceso fácil a cuidados especializados de rutina, con consultas ambulatorias y acceso a números de contacto según corresponda	CVRS ^||^ (SF ^||||^ -36; MLHFQ ^||||||^ ) Costos médicos Rehospitalización dentro de los 30 días Mortalidad	Los puntajes de la CVRS ^||^ mejoraron en ambos grupos a lo largo del año (p < 0,001), pero no hubo diferencias significativas entre los grupos. No se observaron diferencias en la mortalidad (p = 0,575), la tasa de readmisión (p = 0,627) o los costos relacionados con hospitalizaciones y visitas a urgencias entre los grupos.
Cui, et al. ^( 60 )^ 2013 Canadá	Individuos con diagnóstico de IC* n:174 (GC ^†^ :55| GI ^‡^ Teléfono:61 | GI ^‡^ Telemonitoreo:58) Seguimiento: 12 meses	Programa de líneas de salud, compuesto por atención de enfermería con orientación en salud para autogestión y herramientas educativas. El GI ^‡^ Telemonitoreo incluyó monitoreo domiciliario.	Llamadas telefónicas de enfermería Monitores de PA ^¶¶^ Balanza Herramientas educativas en salud	Los cuidados habituales no fueron descritos	Costos y cuidados en salud CVRS ^||^ (SF ^||||^ -36) Autocuidado ( *Revised Self-Care Behavior Scale* ) Satisfacción ( *Client Satisfaction Questionnaire* )	Ambas intervenciones fueron más eficaces y menos costosas que el tratamiento estándar. Hubo una mejora significativa en el comportamiento de autocuidado y en la CVRS ^||^ (p < 0,05), con puntuaciones de utilidad más altas y significativas en los grupos de intervención.
Hawkes, et al. ^( 61 )^ 2012 Australia	Individuos con diagnóstico de IAM ^||||||||^ n:430 (GC ^†^ :215 | GI ^‡^ :215) Seguimiento: 6 meses	*Coaching* telefónico sobre el manejo de factores de riesgo para enfermedades cardíacas Recurso educativo “ *My Heart My Life* ”	Líneas telefónicas de telemedicina o soporte	Recurso educativo “ *My Heart My Life* ” y boletines informativos cada tres meses	CVRS ^||^ (SF ^||||^ -36) Actividad física ( *Active Australia Survey* ) Satisfacción Factores de riesgo para enfermedades cardíacas	A los 6 meses, el GI ^‡^ mostró puntuaciones más altas de CVRS ^||^ en comparación con el GC ^†^ en los componentes mental (IC 95% 0,5 a 4,9; p = 0,02), físico (IC 95% 0,1 a 4,5; p = 0,04) y emocional (IC 95% 0,2 a 5,2; p = 0,03). El GI ^‡^ también fue más propenso a estar suficientemente activo (p = 0,02) y mantener un peso saludable (p = 0,05) en comparación con el GC ^†^ .
Seto, et al. ^( 62 )^ 2012 Canadá	Individuos con diagnóstico de IC* n: 100 (GC ^†^ :50 | GI ^‡^ :50) Seguimiento: 6 meses	Sistema de telemonitoreo además de la atención estándar	Sistema de telemonitoreo: Registros diarios de peso, PA ^¶¶^ y ECG ^††††^ Cuestionario de síntomas en el móvil Envío de mensajes Sitio web Llamadas telefónicas de recordatorio Envío de alertas por correo electrónico al cardiólogo Contacto telefónico con el paciente	Cuidados regulares que incluían visitas clínicas programadas, educación sobre IC* durante las consultas y acceso a soporte telefónico sin intervenciones adicionales del estudio.	Niveles de péptido natriurético cerebral Autocuidado ( *Self-Care of Heart Failure Index)* CVRS ^||^ (MLHFQ ^||||||^ )	El GI ^‡^ mostró una mejora significativa en la CVRS ^||^ (p = 0,05) y un mayor mantenimiento del autocuidado (p = 0,03) en comparación con el GC ^†^ . Ambos grupos mejoraron los niveles de péptido natriurético cerebral, el manejo del autocuidado y la fracción de eyección ventricular.
Blasco, et al. ^( 63 )^ 2012 España	Individuos sobrevivientes de SCA ^¶^ n: 203 (GC ^†^ :101 | GI ^‡^ :102) Seguimiento: 12 meses	Telemonitoreo y envío de mensajes de texto con recomendaciones	Aplicación *web* para envío de mensajes de texto Teléfono móvil Envío de recomendaciones por celular Monitor de PA ^¶¶^ y glucómetro	Tres consultas con cardiólogo; recomendaciones escritas y verbales sobre prevención de enfermedades cardiovasculares	CVRS ^||^ (SF ^||||^ -36) Ansiedad ( *State - Trait Anxiety Inventory* ) Mejora de factores de riesgo cardiovascular	No se encontraron diferencias significativas en las puntuaciones de CVRS ^||^ entre los grupos al inicio o al final del estudio. Sin embargo, en el dominio de “salud física” del SF ^||||^ -36, el GI ^‡^ tuvo un aumento de 2,8 puntos (p = 0,011). El GI ^‡^ también tuvo una mayor probabilidad de mejorar su perfil de factores de riesgo cardiovascular (riesgo relativo 1,4; IC 95% 1,1-1,7; p = 0,010) en comparación con el GC ^†^ .
Koehler, et al. ^( 64 )^ 2011 Alemania	Individuos con diagnóstico de IC* n:710 (GC ^†^ :356|GI ^‡^ :354) Seguimiento: 12 meses	Sistema de autogestión remoto basado en telemedicina	Sistema de telemonitoreo remoto, compuesto por: Asistente digital personal ECG ^††††^ Balanza Monitor de PA ^¶¶^ Centro de telemedicina con soporte médico las 24 horas	No detallada, citada como recomendaciones estándar de las guías para el tratamiento de la IC*	Mortalidad Hospitalizaciones Depresión (PHQ-9 ^§§§§^ ) CVRS ^||^ (SF ^||||^ -36)	La intervención no tuvo un impacto significativo en la mortalidad por todas las causas ni en la mortalidad cardiovascular o las hospitalizaciones por IC* en comparación con el GC ^†^ . Sin embargo, hubo una mejora significativa en la puntuación de funcionamiento físico en el SF ^||||^ -36 en el GI ^‡^ durante el período del estudio (p < 0,05).
Baker, et al. ^( 65 )^ 2011 Estados Unidos de América	Individuos con diagnóstico de IC* n: 605 (GC ^†^ : 303 | GI ^‡^ : 302) Seguimiento: 1 mes	Teleorientación para el autocuidado, iniciada con una sesión educativa de 40 minutos sobre el manejo de la IC*	Llamadas telefónicas de seguimiento con orientación y educación en salud	Sesión educativa de 40 minutos sobre el manejo de la IC*, otros cuidados habituales no detallados.	CVRS ^||^ (HFSS ^¶¶¶¶^ ) Conocimiento ( *Improving Chronic Illness Care Evaluation* )	No hubo cambios significativos en la CVRS ^||^ del GC ^†^ desde el inicio hasta el final del seguimiento, pero en el GI ^‡^ hubo una mejora de 58,6 (±22,2) al inicio a 65,3 (±22,4) al final de 30 días (p = 0,001). El GI ^‡^ también mostró un mayor aumento en el conocimiento general (0,70 vs. 0,30; p = 0,008) y en la autoeficacia (0,4 vs. 1,0; p = 0,006).
Copeland, et al. ^( 66 )^ 2010 Estados Unidos de América	Individuos con diagnóstico de IC* n:458 (GC ^†^ :238|GI ^‡^ :220) Seguimiento: 12 meses	Programa de Gestión de Enfermedades por Teléfono, que incluía llamadas programadas por enfermeros para educación, *coaching* para el cambio de comportamiento y monitoreo de síntomas	Llamadas telefónicas programadas por enfermeros Plan de autogestión individualizado Línea de asesoramiento de enfermería disponible 24 horas Alertas por fax a los médicos sobre signos de descompensación	Cuidados habituales no descritos	CVRS ^||^ (SF ^||||^ -8) Costos en salud Supervivencia Adherencia y satisfacción con los cuidados	Después de un año, no hubo diferencias clínicas significativas entre los grupos. Los costos relacionados con la IC* y los costos totales fueron más altos en el GI ^‡^ . Este grupo también informó una mejor adherencia al monitoreo del peso y a las recomendaciones de ejercicio. No hubo diferencias en la CVRS ^||^ , las hospitalizaciones o la supervivencia entre los grupos.
Balk, et al. ^( 67 )^ 2008 Países Bajo	Individuos con diagnóstico de IC* n: 214 (GC ^†^ :113| GI ^‡^ :101) Seguimiento: 9,6 meses	Sistema Motiva ^®^ de educación en salud y autogestión	Canal de televisión con material educativo Recordatorios de medicación Cuestionarios relacionados con la salud SMS ^§§^ motivacionales Telemonitoreo de PA ^¶¶^ y peso Contacto telefónico por parte de enfermeras	Seguimiento estándar ofrecido por cardiólogos y enfermeras de IC* según la práctica local	Días de hospitalización/año CVRS ^||^ (SF ^||||^ -36; MLHFQ ^||||||^ ) Conocimiento de la enfermedad Autocuidado ( *European Heart Failure Self- care Behaviour Scale* )	No se encontraron diferencias significativas en los desenlaces primarios, la CVRS ^||^ o el comportamiento de autocuidado. Sin embargo, el conocimiento sobre IC* aumentó significativamente más en el GI ^‡^ en comparación con el GC ^†^ (p < 0,001).
Schwarz, et al. ^( 68 )^ 2008 Estados Unidos de América	Individuos con diagnóstico de IC* o cuidadores n:102 (GC ^†^ :51| GI ^‡^ :51) Seguimiento: 3 meses	Sistema de monitoreo remoto, asociado a seguimiento regular por profesionales de la salud	Sistema de telemonitoreo vinculado a la línea telefónica del paciente: Medición diaria de peso Cuestionario sobre síntomas, adherencia a la medicación y consumo de sodio Contacto telefónico con el cuidador si era necesario	Cuidados habituales no detallados, mencionados como cuidados post-alta	Rehospitalizaciones Visitas a urgencias Costos CVRS ^||^ (MLHFQ ^||||||^ )	El estudio piloto no encontró diferencias estadísticamente significativas en el GI ^‡^ respecto a rehospitalizaciones, visitas a urgencias, costos o riesgo de rehospitalización. La CVRS ^||^ mejoró significativamente en ambos grupos en la visita de seguimiento a los 90 días (p < 0,0001).
Wakefield, et al. ^( 69 )^ 2008 Estados Unidos de América	Individuos con diagnóstico de IC* n:148 (GC ^†^ :49|GI ^‡^ Vídeo:52| GI ^‡^ Teléfono:47) Seguimiento: 12 meses	Programa de telesalud basado en seguimiento telefónico o por video, monitoreo de síntomas y educación en salud	*Checklist* de revisión de síntomas Control diario de peso, PA ^¶¶^ y circunferencia del tobillo Contacto telefónico y videollamadas Entrenamiento en habilidades conductuales Estrategias para maximizar el automonitoreo y la autoeficacia Revisión y refuerzo de planes de alta	Orientación estándar al alta hospitalaria y contacto telefónico con enfermería de caso si era necesario	Número de rehospitalizaciones Tiempo hasta la primera rehospitalización Visitas a urgencias Supervivencia CVRS ^||^ (MLHFQ ^||||||^ )	No hubo diferencias en días de hospitalización o visitas a urgencias entre los tres grupos. Las puntuaciones de CVRS ^||^ mejoraron en todos los grupos a lo largo del tiempo (F = 8,90; p = 0,0002). La magnitud de la mejora fue mayor en el GI ^‡^ Teléfono, seguido por el GI ^‡^ Vídeo y el GC ^†^ .
Woodend, et al. ^( 70 )^ 2008 Canadá	Individuos con diagnóstico de IC* o angina n:249 (GC ^†^ :125|GI ^‡^ :124) Seguimiento: 12 meses	Programa de telemonitoreo domiciliario compuesto por videollamadas, monitoreo de parámetros vitales, soporte telefónico y contenido educativo estructurado	Equipos de monitoreo domiciliario (balanza, monitor de PA ^¶¶^ , ECG ^††††^ de 12 derivaciones) Videollamadas con enfermería Registro electrónico de datos Línea de asesoramiento de enfermería disponible 24 horas	Tratamiento habitual tras el alta hospitalaria para pacientes con angina o IC*, sin mayores detalles	Status funcional (MLHFQ ^||||||^ ; *Seattle Angina Questionnaire* ) CVRS ^||^ (SF ^||||^ -36) Uso de recursos de salud (hospitalizaciones, días de internación, visitas a urgencias)	La CVRS|| fue mejor en pacientes con IC* del GI ^‡^ en comparación con el GC ^†^ en cinco de las ocho subescalas del SF ^||||^ -36 a los 3 meses (p < 0,05). Las diferencias más significativas se detectaron en este período. Ambos grupos mostraron mejoras significativas en la CVRS ^||^ a lo largo del tiempo en todas las subescalas del SF ^||||^ -36.
López Cabezas, et al. ^( 71 )^ 2006 España	Individuos con diagnóstico de IC* n: 134 (GC ^†^ : 64 | GI ^‡^ :70) Seguimiento: 12 meses	Programa de orientación en salud liderado por farmacéutico, con soporte telefónico para dudas o problemas	Material educativo escrito y audiovisual sobre síntomas y patogénesis de la IC* Llamadas telefónicas por parte del farmacéutico	Consultas regulares con cardiólogo y farmacéutico	Rehospitalizaciones Adherencia al tratamiento CVRS ^||^ (EQ-5D ^‡‡^ ) Satisfacción del paciente	El GI ^‡^ mostró mayor adherencia al tratamiento (85,0% vs 73,9%), menor número de días de hospitalización (GC ^†^ : 9,6 días vs GI ^‡^ : 5,9 días) y mayor satisfacción (p = 0,026). Hubo una reducción significativa de rehospitalizaciones en el GI ^‡^ (54% al inicio vs 32% al final), aunque no hubo diferencias significativas en la CVRS ^||^ .
Riegel et al. ^( 72 )^ 2006 Estados Unidos de América	Individuos con diagnóstico de IC* n: 134 (GC ^†^ : 65 | GI ^‡^ : 69) Seguimiento: 6 meses	Gestión de casos por teléfono, con enfoque en educación, monitoreo y orientación, además de envío de informes y contacto con el equipo médico	*Software* de soporte a la decisión *(At Home with Heart Failure)* Envío mensual de material educativo impreso Soporte telefónico	Cuidado habitual no estandarizado basado en educación sobre el manejo de la IC*	Hospitalizaciones, días en el hospital y costos de cuidados agudos (por IC* y por todas las causas) Rehospitalizaciones múltiples Mortalidad por todas las causas CVRS ^||^ (EQ-5D ^‡‡^ )	No se encontraron diferencias significativas entre los grupos en la tasa de rehospitalización, días de hospitalización, costos de cuidado, mortalidad, CVRS ^||^ o depresión.
Benatar, et al. ^( 73 )^ 2003 Estados Unidos de América	Individuos con diagnóstico de IC* n:216 (GC ^†^ :108| GI ^‡^ :108) Seguimiento: 3 meses	Sistema de telemonitoreo domiciliario, basado en dispositivos de autogestión con evaluaciones telefónicas y establecimiento de metas clínicas individuales	Dispositivos de monitoreo domiciliario para medir peso, PA ^¶¶^ , FC** y nivel de saturación de oxígeno Equipo de telegestión con contactos vía llamadas telefónicas	Cuidados habituales compuestos por seguimiento en centros de salud y visitas de enfermería domiciliarias	CVRS ^||^ (MLHFQ ^||||||^ | *70-item Quality of Life Index – Cardiac* ) Ansiedad y depresión (HADS*****) Autoeficacia ( *Heart Failure Self-Efficacy Scale 30* ) Hospitalizaciones Costos en salud	El GI ^‡^ tuvo menos readmisiones por IC* (13 vs. 24; p ≤ 0,001), menor tiempo de hospitalización (49,5 vs. 105,0 días; p ≤ 0,001) y costos hospitalarios más bajos a los 3 meses ($65.023 vs. $177.365; p ≤ 0,02). Ambos grupos experimentaron mejoras en la CVRS ^||^ tras la intervención.
Barnason, et al. ^( 74 )^ 2003 Estados Unidos de América	Individuos con IC* isquémica sometidos a revascularización miocárdica n:35 (GC ^†^ :17| GI ^‡^ :18) Seguimiento: 3 meses	*Health Buddy,* evaluación de síntomas y educación sobre la recuperación post-revascularización, manejo de síntomas, funcionamiento y adhesión a la modificación de factores de riesgo de la EAC ^‡‡‡^	Dispositivo de telecomunicación conectado al teléfono del paciente ( *Health Buddy* ) Sitio *web* para almacenamiento de los datos recolectados	Educación y asesoramiento sobre cuidados para la recuperación postquirúrgica, autocuidado y modificación de factores de riesgo de la EAC ^‡‡‡^ , y prescripción de ejercicios domiciliarios proporcionados a todos los pacientes sometidos a revascularización antes del alta hospitalaria	Autoeficacia (Escala de Expectativa de Eficacia de Barnason) CVRS ^||^ (MOS SF-36 ^†††††^ )	El GI ^‡^ presentó puntuaciones medias ajustadas de autoeficacia significativamente mayores (p < 0,05), así como niveles medios ajustados de funcionamiento físico, general, mental y de vitalidad superiores (p < 0,05). También mostró mayor adhesión al ejercicio (p < 0,01) y menor estrés reportado (p < 0,01) después de tres meses de la cirugía en comparación con el GC ^†^ . Se observó una mejora significativa en los puntajes de dolor y funcionamiento emocional a lo largo del tiempo (p < 0,05).

*IC = Insuficiencia cardíaca; ^†^GC = Grupo control; ^‡^GI = Grupo intervención; ^§^NYHA = *New York Heart Association*; ^||^CVRS = Calidad de vida relacionada con la salud; ^¶^SCA = Síndrome coronariano agudo; **FC = Frecuencia cardíaca; ^††^VO_2_ = Volumen de Oxígeno; ^‡‡^EQ-5D = *EuroQol-5 dimension-5 levels*; ^§§^SMS = Mensajes de texto curtos; ^||||^SF - *Short form health survey*; ^¶¶^PA = Presión arterial; ***WHO-5 = Índice de Bienestar de la *World Health Organization-5 ítems*; ^†††^KCCQ = *Kansas City Cardiomyopathy Questionnaire;* ^‡‡‡^EAC = Enfermedad arterial coronaria; ^§§§^EQ-VAS = *EuroQol Visual Analogue Scale*; ^||||||^MLHFQ = *Minnesota Living With Heart Failure Questionnaire*; ^¶¶¶^TC6M = Test de marcha de 6 minutos; ****HeartQoL = *Health-related quality of life questionnaire*; ^††††^ECG = Electrocardiograma; ^‡‡‡‡^FA = Fibrilación auricular; ^§§§§^PHQ-9 = *Patient Health Questionnaire-9 ítems;* ^||||||||^IAM = Infarto agudo de miocardio; ^¶¶¶¶^HFSS = *Heart Failure Symptom Scale*; *****HADS = *Hospital Anxiety and Depression Scale;* ^†††††^MOS SF-36 = *Medical Outcomes Short-Form Health Survey*-36 ítems

Esta revisión sistemática revela que la literatura científica sobre el tema está en constante expansión, especialmente con la inclusión de estudios de alta calidad metodológica. Los primeros artículos fueron publicados en 2003^(73-74)^, y desde entonces no ha habido una reducción en el número de publicaciones. Por el contrario, se observó un aumento significativo de estudios, especialmente tras el inicio de la pandemia, cuando los resultados relacionados con el uso de la telemedicina ganaron aún más relevancia.

Los artículos que cumplieron con los criterios de inclusión fueron sometidos a una evaluación crítica de su calidad metodológica (n=44). Los resultados variaron entre un 53,85% y un 92,31% de congruencia con el instrumento utilizado^(29)^. Es importante señalar que las principales inconsistencias se encontraron en el cegamiento de los evaluadores de resultados, de los aplicadores de la intervención y de los pacientes. El detalle completo de las evaluaciones puede consultarse en el Material Suplementario (https://doi.org/10.48331/scielodata.MI2JBD).

En relación con las características de la población de los estudios incluidos, la mayoría de los pacientes fue seguida por insuficiencia cardíaca (IC) (68,18%, n=30)^(37,43-46,51-53,59-60,62,65-67,63-64,66,68-70,72-74)^, síndrome coronario agudo (SCA) (15,22%, n=7)^(32-33,36,40,58,61,63)^ o enfermedad arterial coronaria (EAC) (10,64%, n=5)^(35,40,42,54,57)^. En cuatro estudios^(40,43,54,71)^, la intervención estuvo dirigida a una población mixta de pacientes con diferentes enfermedades cardíacas.

Respecto a los recursos e intervenciones adoptadas, las modalidades de telemedicina variaron considerablemente, destacándose las intervenciones híbridas que emplean múltiples herramientas para maximizar la eficacia del tratamiento. El telemonitoreo fue la intervención más frecuentemente utilizada, presente en 31 estudios (68,89%)^(31-32,34-35,38-46,48-49,51-53,56-60,62-64,67,68,70,73-74)^. Además del telemonitoreo, otra intervención que destacó como recurso de apoyo ampliamente utilizado fue la llamada telefónica, presente en 23 artículos (51,11%)^(34,36,37,39,41-42,45,47,51-52,57,60-62,65-73)^. En la mayoría de los casos, estas llamadas fueron utilizadas como apoyo complementario^(34,39,41-42,45,47,51-52,57,60-62,67-68,70,73)^, facilitando la comunicación y el seguimiento de los pacientes. Sin embargo, en algunas situaciones, constituyeron la principal forma de intervención^(36-37,65-66,69,71-72)^, lo que resalta su importancia en contextos donde otras tecnologías pueden no estar disponibles o ser viables.

Además de estas intervenciones, la telerrehabilitación fue identificada en 13 estudios (28,88%)^(32,35,39,41-42,47,50,52,54-58)^. Entre los enfoques híbridos, la combinación de telemonitoreo y telerrehabilitación fue predominante, presente en nueve estudios (20%)^(32,35,39,41-42,52,56-58)^. Este enfoque ofrece monitoreo en tiempo real de los signos vitales y el progreso del paciente, además de la prescripción remota de ejercicios. Por su parte, el uso de servicios de mensajes cortos (SMS) de manera aislada es menos común, citado en solo un artículo^(33)^, pero frecuentemente combinado con otras intervenciones^(36,43,47,54-55,62)^ debido a su capacidad de enviar recordatorios, instrucciones y motivación continua.

De los 44 estudios analizados, los desenlaces primarios o secundarios incluyeron una comparación pre y postintervención de la CVRS entre los grupos de intervención y control. En este sentido, se observó una diversidad en los instrumentos utilizados, con un 81,81% (n=36) de los estudios optando por el uso de un único cuestionario^(31-33,36-42,44 - 49, 51- 54, 56- 58, 60- 66, 68- 72, 74)^, mientras que el 11,36% (n=5) empleó dos cuestionarios^(50,55,59,67,73)^ y el 6,81% (n=3) recurrió a la aplicación de tres cuestionarios^(34-35,43)^.

Entre las escalas empleadas, la *Short Form Health Survey-36* (SF-36) fue la más común, adoptada en 17 (38,63%) de los ensayos clínicos^(34,39,41-44,52,55,59-61,63-64,67,70-74)^. Por otro lado, 12 (26,66%) estudios utilizaron la escala *Minnesota Living With Heart Failure Questionnaire* (MLHFQ)^(38,45-48,50,59,62,67-69,73)^. Las variaciones del cuestionario *EuroQol* fueron empleadas en diez estudios (24,44%)^(32,35-36,43,49-50,55,58,71-72)^.

La evaluación GRADE^(30)^ reveló un riesgo serio de sesgo, y la heterogeneidad en los informes de medición de los efectos impidió la realización de un análisis agrupado. La Figura 3 presenta el perfil de evidencias, separando los estudios según los cuestionarios utilizados y mostrando que la certeza de las evidencias varió de baja a muy baja.

Con respecto a los resultados de la CVRS, ninguno de los estudios seleccionados presentó un efecto de empeoramiento de la CVRS en el GI basado en telemedicina a lo largo del seguimiento. Además, el 45,45% (n = 20) de los estudios mostraron una relación positiva, con significancia estadística, en los puntajes de CVRS en el grupo de intervención^(33-34,41,45,47-49,51,54-55-58-60-65-68-70)^. Sin embargo, el análisis exhaustivo de los estudios reveló que el 11,36% (n = 5) no observó ningún impacto en la CVRS durante el seguimiento^(36,38,40,66-67)^. Por otro lado, el 43,18% (n = 19) demostraron mejoras positivas en la CVRS, sin alcanzar una diferencia estadísticamente significativa entre los grupos^(31-32,35,37,39,42-44,46,50,52-53,56,57,59,71,74)^. La Tabla 1 proporciona una síntesis detallada de los resultados en CVRS de los estudios evaluados.

**Figura 3 -:** Perfil de evidencia GRADE

**Evaluación de Certeza**							**N** ^o^ **de pacientes**		**Efecto**		**Garin, et al**	**Importancia**
**N** ^o^ **de los estudios**	**Diseñodel estudio**	**Riesgo de sesgo**	**Inconsistencia**	**Evidencia indirecta**	**Imprecisión**	**Otras consideraciones**	**Telemedicina**	**Cuidado habitual**	Relativo (95% IC*)	Absoluto (95% IC*)		
Calidad de vida relacionada con la salud (seguimiento: media 8.9 meses; evaluado con: EuroQol (EQ-5D ^†^ | EQ-5D-5L ^‡^ | EQ-5D-Y ^§^ ); Escala de: 0 para 100)												
7	ensayos clínicos aleatorizados	muy grave ^||^	no grave	no grave	no grave	ninguno	649	613	-	**0** (0 para 0)	⨁⨁◯◯ | CRÍTICO | Baja |	
Calidad de vida relacionada con la salud (seguimiento: media 7.8 meses; evaluado con: MLHFQ ^¶^ | HFSS**; Escala de: 0 para 105)												
13	ensayos clínicos aleatorizados	muy grave ^††^	no grave	no grave	grave ^‡‡^	ninguno	1309	1102	-	**0** (0 para 0)	⨁ ◯◯◯ | CRÍTICO | Muy baja |	
Calidad de vida relacionada con la salud (seguimiento: media 8.8 meses; evaluado con: SF ^§§^ -36 | SF ^§§^ -12 | SF ^§§^ -8; Escala de: 0 para 100)												
16	ensayos clínicos aleatorizados	muy grave ^||||^	no grave	no grave	no grave	ninguno	2850	2800	-	**0** (0 para 0)	⨁ ⨁ ◯◯ | CRÍTICO | Baja |	
Calidad de vida relacionada con la salud (seguimiento: media 10.5 meses; evaluado con: KCCQ ^¶¶^ ; Escala de: 0 para 100)												
4	ensayos clínicos aleatorizados	muy grave***	no grave	no grave	no grave	ninguno	1497	1528	-	**0** (0 para 0)	⨁ ⨁ ◯◯ | CRÍTICO | Baja |	
Calidad de vida relacionada con la salud (seguimiento: media 9 meses; evaluado con: HeartQol ^†††^ ; Escala de: 0 para 3)												
2	ensayos clínicos aleatorizados	muy grave ^‡‡‡^	no grave	no grave	no grave	ninguno	127	126	-	**0** (0 para 0)	⨁ ⨁ ◯◯ | CRÍTICO | Baja |	
Calidad de vida relacionada con la salud (seguimiento: media 3 meses; evaluado con: MacNew; Escala de: 1 para 7)												
2	ensayos clínicos aleatorizados	muy grave ^‡‡‡^	no grave	no grave	no grave	ninguno	67	64	-	**0** (0 para 0)	⨁ ⨁ ◯◯ | CRÍTICO | Baja |	

*IC = Intervalo de confianza; ^†^EQ-5D = *EuroQol-5 Dimension*; ^‡^EQ-5D-5L= *EuroQol-5 dimension-5 levels*; ^§^EQ-5D-Y = *EuroQol-5 dimension-young*; ^||^Muy grave = 7 estudios con preocupaciones sobre sesgo de aleatorización (ocultación de la asignación, cegamiento de los profesionales que realizaron las intervenciones) y 5 estudios con sesgo en el cegamiento de los evaluadores de resultados; ^¶^MLHFQ = *Minnesota Living with Heart Failure Questionnaire*; **HFSS = *Heart Failure Symptom Scale*; ^††^Muy grave = 12 estudios con preocupaciones sobre sesgo de aleatorización (ocultación de la asignación, cegamiento de los profesionales y evaluadores de resultados); ^‡‡^Grave= 7 estudios con alto riesgo de imprecisión debido a falta de datos de resultados; ^§§^SF = *Short form health survey*; ^||||^Muy Grave = 15 estudios con algunas preocupaciones respecto al sesgo de aleatorización (ocultación de la asignación, cegamiento de los profesionales que realizaron las intervenciones) y 11 estudios con sesgo relacionado con el cegamiento de los evaluadores de resultados; ^¶¶^KCCQ= *Kansas City Cardiomyopathy Questionnaire*; ***Muy grave = 4 estudios con algunas preocupaciones respecto al sesgo de aleatorización (ocultación de la asignación, cegamiento de los profesionales que realizaron las intervenciones) y 3 estudios con sesgo relacionado con el cegamiento de los evaluadores de resultados; ^†††^HeartQol = *Health-related Quality of life questionnaire*; ^‡‡‡^Muy grave = 2 estudios con algunas preocupaciones respecto al sesgo de aleatorización (ocultación de la asignación, cegamiento de los profesionales que realizaron las intervenciones)

**Tabla 1 -:** Síntesis de los resultados de calidad de vida relacionada con la salud (CVRS) (n = 44). São Paulo, Brasil, 2024

**Estudio**	**Seguimiento (meses)**	**Instrumento**	**Grupo**	**n**	Puntaje Inicial Media (DE*)	Puntaje Final Media (DE*)	**p-valor**	Cambio Media (DE*)	**Diferencia entre los grupos (p-valor)**
Choi, et al. ^( 31 )^	3	MacNew	GC ^†^	38	5,45 (0,86)	5,34 (1,09)	-	-	0,771
			GI ^‡^	38	5,62 (0,82)	5,62 (0,78)	-	-	
Dalli Peydró, et al. ^( 32 )^	10	EQ-5D-5L ^§^	GC ^†^	28	70 (65-85)	80 (70-90)	0,008	-	>0,800
			GI ^‡^	31	75 (60-90)	88 (68-90)	0,064	-	
Chow, et al. ^( 33 )^	12	SF ^||^ -12	GC ^†^	708	-	PS ^¶^ : 47,6(-)MCS**: 51,2 (-)	-	-	PS ^¶^ : 0,045MCS**: 0,204
			GI ^‡^	716	-	PS ^¶^ : 47,6 (9,5)MCS**: 48,9 (9,6)	-	-	
Voller, et al. ^( 34 )^	12	KCCQ ^††^	GC ^†^	319	59,3 (24,0)	-	-	-	<0,01
			GI ^‡^	302	59,6 (23,0)	-	-	-	
Brouwers, et al. ^( 35 )^	12	EQ-5D-5L ^§^	GC ^†^	147	0,815 (0,010)	0,848 (0,016)	-	-	0,002
			GI ^‡^	153	0,814 (0,011)	0,851 (0,015)	-	-	
Maddison, et al. ^( 36 )^	13	EQ-5D ^‡‡^	GC ^†^	153	64 (22)	-	-	-	-
			GI ^‡^	153	62 (22)	-	-	-	-
Collins, et al. ^( 37 )^	3	KCCQ ^††^	GC ^†^	245	-	-	-	-	0,25
			GI ^‡^	246	-	-	-	-	
Clays, et al. ^( 38 )^	6	MLHFQ ^§§^	GC ^†^	22	30,0 (13,5)	-	0,58	1,7 (13,8)	0,5
			GI ^‡^	34	32,1 (22,9)	-	0,7	-1,0 (14,4)	
Batalik, et al. ^( 39 )^	3	SF ^||^ -36	GC ^†^	28	50,9 (8,8)	61,5 (7,1)	0,01	-	0,56
			GI ^‡^	28	53,1 (6,7)	62 (7)	0,01	-	
Lunde, et al. ^( 40 )^	12	HeartQoL ^||||^	GC ^†^	147	2,48 (0,54)	2,57 (0,51)	-	-	<0,05
			GI ^‡^	153	2,43 (0,59)	2,64 (0,51)	-	-	
		EQ-5D ^‡‡^	GC ^†^	147	72 (14)	75 (12)	-	-	<0,001
			GI ^‡^	153	69 (18)	78 (16)	-	-	
Piotrowicz, et al. ^( 41 )^	14-26	SF ^||^ -36	GC ^†^	425	88,8 (14,1)	88,9 (14,4)	-	-	0,008
			GI ^‡^	425	89,7 (12,6)	91,2 (12,8)	-	-	
Ávila, et al. ^( 42 )^	3	SF ^||^ -36	GC ^†1^	30	73,3 (15,1)	76,4 (16,4)	-	-	0,06
			GC ^†2^	30	79,8 (16,1)	82,6 (15,8)	-	-	
			GI ^‡^	30	82,2 (13,3)	82,6 (13)	-	-	
Cáceres, et al. ^( 43 )^	6	SF ^||^ -36	GC ^†^	123	PS ^¶^ : 47,6 (9,5)MCS**: 48,9 (9,6)	PS ^¶^ : 50,1 (8,5)MCS**: 52,7 (7,5)	-	-	PS ^¶^ : 0,37MCS**: 0,74
			GI ^‡^	115	PS ^¶^ : 49,6 (8,6)MCS**: 50,8 (8,5)	PS ^¶^ : 52,5 (9,1)MCS**: 53,1 (8,6)	-	-	
		AFEQT ^¶¶^	GC ^†^	123	64,8 (25,2)	80,2 (20,9)	-	-	0,09
			GI ^‡^	115	66,3 (21)	79 (20,3)	-	-	
		EQ-5D ^‡‡^	GC ^†^	123	0,85 (0,21)	0,91 (0,13)	-	-	0,98
			GI ^‡^	115	0,88 (0,16)	0,94 (0,14)	-	-	
Cichosz, et al. ^( 44 )^	12	SF ^||^ -36	GC ^†^	154	PS ^¶^ : 40,3 (9,2)MCS**: 48,9 (11,4)	PS ^¶^ : 40,67 (10,2)MCS**: 46,65 (12,1)	-	-	NS***
			GI ^‡^	145	PS ^¶^ : 40,3 (9)MCS** 47,4 (10)	PS ^¶^ : 40,58 (9,7)MCS**: 50,01 (11,5)	-	-	<0,05
		KCCQ ^††^	GC ^†^	154	62,5 (20,4)	65,56 (21,2)	-	-	<0,01
			GI‡	145	64,7 (18,8)	66,31 (20,7)	-	-	
Mizukawa, et al. ^( 45 )^	24	MLHFQ ^§§^	GC ^†^	19	32,2 (27,8)	-	-	-	0,564
			GI ^‡1^	20	37,3 (22,7)	-	-	-	
			GI ^‡2^	20	47,5 (26,8)	-	-	-	
Pekmezaris, et al. ^( 46 )^	3	MLHFQ ^§§^	GC ^†^	58	59,9	27,8	-	-	0,5
			GI ^‡^	46	62,7	36,3	-	-	
Peng, et al. ^( 47 )^	4	MLHFQ ^§§^	GC ^†^	49	48,77 (12,21)	49,63 (12,39)	-	-	0,072
			GI ^‡^	49	49,43 (12,25)	42,32 (8,83)	-	--	
Wagenaar, et al. ^( 48 )^	12	MLHFQ ^§§^	GC ^†^	150	23,0 (32,5)	26,5	-	-	0,003
			GI ^‡1^	150	24,0 (31,0)	28,3	-	-	
			GI ^‡2^	150	23,0 (27,8)	25,5	-	-	
Guo, et al. ^( 49 )^	3	EQ-5D-Y ^†††^	GC ^†^	96	71,3	69,9	-	-	<0,05
			GI ^‡^	113	86,5	87,2	-	-	
Hwang, et al. ^( 50 )^	6	MLHFQ ^§§^	GC ^†^	29	47 (19)	33 (21)	-	-	-
			GI ^‡^	24	41 (22)	34 (23)	-	-	
Jayaram, et al. ^( 51 )^	6	KCCQ ^††^	GC ^†^	765	58,6 (24,9)	68,5 (26,4)	0,11	-	0,01
			GI ^‡^	756	60,7 (24,1)	72,3 (24,4)	0,01	-	
Piotrowicz, et al. ^( 52 )^	2	SF ^||^ -36	GC ^†^	34	73,6 (25,6)	67,4 (25,9)	NS**	-	0,0001
			GI ^‡^	77	79,0 (31,3)	70,8 (30,3)	0,001	-	
Bekelman, et al. ^( 53 )^	12	KCCQ ^††^	GC ^†^	193	36,9 (14,6)	54,2	-	-	0,97
			GI ^‡^	199	37,9 (13,3)	53,6	-	-	
Frederix, et al. ^( 54 )^	6	HeartQoL ^||||^	GC ^†^	70	2,31 (0,59)	2,32 (0,58)	0,21	-	0,01
			GI ^‡^	70	2,27 (0,63)	2,53 (0,44)	0,01	-	
Maddison, et al. ^( 55 )^	6	SF ^||^ -36	GC ^†^	86	PS ^¶^ : 51,9 (5,8)MCS**: 51,53 (8,4)	PS ^¶^ : 51,9(-)MCS**: 54(-)	-	-	PS ^¶^ : 0,2MCS**: 0,61
			GI ^‡^	85	PS ^¶^ : 51,6 (5,9)MCS**: 52,87 (6,94)	PS ^¶^ : 52,9(-)MCS**: 54,6(-)	-	-	
		EQ-5D ^‡‡^	GC ^†^	86	0,8 (0,1)	0,83(-)	-	-	0,23
			GI ^‡^	85	0,8 (0,1)	0,86(-)	-	-	
Piotrowicz, et al. ^( 56 )^	2	SF ^||^ -36	GC ^†^	75	81,6 (27,3)	62,2 (26,4)	-	-	NS***
			GI ^‡^	77	79,3 (25,6)	70,5 (25,4)	-	-	
Kraal, et al. ^( 57 )^	3	MacNew	GC ^†^	26	5,2 (0,8)	5,8 (0,7)	-	-	0,498
			GI ^‡^	29	5,7 (0,7)	6,1 (0,5)	-	-	
Varnfield, et al. ^( 58 )^	1,5	EQ-5D ^‡‡^	GC ^†^	60	0,83 (0,8–0,9)	0,82 (0,7-0,9)	0,7	-	0,01
			GI ^‡^	60	0,84 (0,8–0,9)	0,92 (0,9–1,0)	<0,001	-	
Blum, et al. ^( 59 )^	12	MLHFQ ^§§^	GC ^†^	102	37 (27)	18 (21)	-	-	NS***
			GI ^‡^	104	42 (23)	24 (24)	-	-	
		SF ^||^ -36	GC ^†^	102	PS ^¶^ : 35 (11)MCS**: 49 (13)	PS ^¶^ : 38 (11)MCS**: 55 (9)	-	-	-
			GI‡	104	PS ^¶^ : 37 (90)MCS**: 49 (12)	PS ^¶^ : 38 (10)MCS**: 52 (11)	-	-	
Cui, et al. ^( 60 )^	12	SF ^||^ -36	GC ^†^	55	44,61 (23,3)	49,05 (19,59)	-	-	0,5341
			GI ^‡1^	61	45,93 (19,47)	55,63 (27,51)	-	-	
			GI ^‡2^	58	44,73 (17,79)	40,17 (23,38)	-	-	
Hawkes, et al. ^( 61 )^	6	SF ^||^ -36	GC ^†^	215	46,7 (9,6)	46,7 (11,1)	-	0,3	0,73
			GI ^‡^	215	45,8 (10,1)	46,4 (11,5)	-	-	
Seto, et al. ^( 62 )^	6	MLHFQ ^§§^	GC ^†^	50	47,8 (22,6)	47,3 (23,4)	0,9	-	0,05
			GI ^‡^	50	50,3 (29,1)	41,4 (26,7)	0,02	-	
Blasco, et al. ^( 63 )^	12	SF ^||^ -36	GC ^†^	101	-	-	-	-	NS***
			GI ^‡^	102	-	-	-	-	
Koehler, et al. ^( 64 )^	12	SF ^||^ -36	GC ^†^	356	-	51,7 (1,4)	-	0,3	<0,05
			GI ^‡^	354	-	53,8 (1,4)	-	-	
Baker, et al. ^( 65 )^	1	HFSS ^‡‡‡^	GC ^†^	303	64,8 (22,4)	64,1 (22,8)	-	-0,6	<0,001
			GI ^‡^	302	58,6 (22,2)	65,3 (22,4)	-	6,7	
Copeland, et al. ^( 66 )^	12	SF ^||^ -8	GC ^†^	238	-	-	-	-	NS***
			GI ^‡^	220	-	-	-	-	
Balk, et al. ^( 67 )^	9,6	MLHFQ ^§§^	GC ^†^	113	-	-	-	-	NS***
			GI ^‡^	101	-	-	-	-	
Schwarz, et al. ^( 68 )^	3	MLHFQ ^§§^	GC ^†^	51	35,8 (21,5)	27,3 (21,6)	-	-	NS***
			GI ^‡^	51	39,5 (23,3)	27,4 (21,7)	-	-	
Wakefield, et al. ^( 69 )^	12	MLHFQ ^§§^	GC ^†^	49	60,6 (19,3)	56,6 (23,9)	-	-	0,0002
			GI ^‡1^	52	58,4 (22,9)	41,5 (26,9)	-	-	
			GI ^‡2^	47	60,2 (24,8)	54,0 (26,0)	-	-	
Woodend, et al. ^( 70 )^	12	SF ^||^ -36	GC ^†^	125	-	-	-	-	-
			GI ^‡^	124	-	-	-	-	
López Cabezas, et al. ^( 71 )^	12	EQ-5D ^‡‡^	GC ^†^	64	65 (17,6)	60,6 (17,8)	-	-	NS***
			GI ^‡^	70	62,3 (17,3)	64 (15,4)	-	-	
Riegel, et al. ^( 72 )^	6	EQ-5D ^‡‡^	GC ^†^	65	57,1 (16,7)	73,7 (17,4)	-	-	-
			GI ^‡^	69	60,4 (19,9)	73,4 (17,4)	-	-	-
		MLHFQ ^§§^	GC ^†^	65	56,1 (16,7)	12,9 (10,9)	-	-	
			GI ^‡^	69	52,7 (19,6)	12,1 (12,3)	-	-	
Benatar, et al. ^( 73 )^	3	MLHFQ ^§§^	GC ^†^	108	77,17 (8,52)	57,72 (16,24)	0,47	-	<0,01
			GI ^‡^	108	77,92 (10,3)	51,64 (17,36)	0,98	-	
Barnason, et al. ^( 74 )^	3	SF ^||^ -36	GC ^†^	17	60,7	69,1	-	-	-
			GI ^‡^	18	60,7	78,3	-	-	

*DE = Desviación estándar; ^†^GC = Grupo control; ^‡^GI = Grupo intervención; ^§^EQ-5D-5L = *EuroQol-5 dimension-5 levels*; ^||^SF = *Short form health survey*; ^¶^PS = Resumen del componente físico del *36-Item Short Form Survey*;**MCS = Resumen del componente mental del *36-Item Short Form Survey*; ^††^KCCQ = *Kansas City Cardiomyopathy Questionnaire*; ^‡‡^EQ-5D = *EuroQol-5 dimension*; ^§§^MLHFQ = *Minnesota Living With Heart Failure Questionnaire*; ^||||^HeartQoL = *Health-related quality of life questionnaire*; ^¶¶^AFEQT = *Atrial Fibrillation Effect on Quality-of-life;* ***NS = No significativo; ^†††^EQ-5D-Y= *EuroQol-5 dimension young*; ^‡‡‡^HFSS =*Heart Failure Symptom Scale*

## Discusión

Las intervenciones de telemedicina enfocadas en el autogestión demostraron una relación positiva en la mejora de la CVRS de pacientes con enfermedades cardíacas en el 45,45% de los estudios incluidos^(33-34,41,45,47-49,51,54-55-58-60-65-68-70)^. Estas intervenciones se aplicaron mayoritariamente a individuos con IC^(37,43-46,51-53,59-60,62,65-67,63-64,66,68-70,72-74)^, SCA^(32-33,36,40,58,61,63)^ y EAC^(35,40,42,54,57)^. Entre los principales recursos utilizados se encontraron el telemonitoreo, las llamadas telefónicas, la telerrehabilitación, los SMS y las videoconsultas^(31-74)^.

La literatura científica sobre telemedicina ha mostrado un crecimiento constante desde la publicación de los primeros estudios en 2003, con un aumento significativo de investigaciones durante la pandemia de COVID-19^(11)^. Esta revisión sistemática analizó 44 ensayos clínicos aleatorizados, que involucraron un total de 12.732 pacientes. A pesar de una ligera disparidad numérica entre los grupos control (6.233) e intervención (6.499), la distribución fue considerada equilibrada por los autores de los estudios incluidos. Los estudios se llevaron a cabo principalmente en Europa (40,91%) y América del Norte (36,36%), con una participación significativa también de Oceanía (13,64%) y Asia (9,09%). Esta distribución geográfica destaca la amplia aceptación y aplicación de las tecnologías de telemedicina en diversos contextos culturales y de infraestructura.

Sin embargo, la expansión de la telemedicina durante la pandemia también evidenció debilidades, desigualdades y limitaciones en los sistemas de salud que anteriormente podrían haber sido menos perceptibles^(11)^. A pesar de la distribución geográfica positiva entre continentes, se identifican brechas geográficas, especialmente en regiones como América Latina y África. La escasez puede atribuirse a la limitada infraestructura tecnológica y a las menores inversiones en investigación sobre telemedicina en estos lugares^(11)^. Esto destaca la necesidad de enfoques multifacéticos para enfrentar estos desafíos y garantizar una representación más amplia de la investigación en telemedicina a escala global.

La medición y evaluación de la CVRS son desafíos frecuentemente enfrentados en investigaciones científicas debido a la naturaleza multidimensional y a las diversas definiciones asociadas a este concepto^(75)^. Así, considerando esta complejidad, se han desarrollado y empleado una variedad de instrumentos para evaluar la CVRS^(75)^. En general, en la presente revisión, se observó el uso de 17 instrumentos diferentes, con el predominio de un único cuestionario^(31-33,36-42,44 - 49, 51- 54, 56- 58, 60- 66, 68- 72, 74)^para la evaluación del desenlace de CVRS.

En este sentido, la elección entre el uso de instrumentos genéricos y específicos para condiciones médicas en la medición de esta variable presenta ventajas distintas. Los instrumentos genéricos permiten la comparación de la CVRS entre diferentes condiciones de salud, ofreciendo una visión amplia y comparativa en diversas situaciones clínicas. Por otro lado, las medidas específicas para condiciones médicas se concentran directamente en la evaluación de la CVRS relacionada con la condición en estudio, lo que los convierte en instrumentos clínicamente más relevantes para comprender el impacto específico de la enfermedad en la vida de los pacientes^(76)^.

De este modo, el uso de perspectivas duales proporciona interpretaciones más completas y complejas en el abordaje de la CVRS en las investigaciones en salud, permitiendo un análisis más preciso e informativo de los desafíos enfrentados por los pacientes en diferentes contextos de vida^(76)^. En consecuencia, en esta revisión, solo nueve estudios realizaron el análisis de la CVRS a partir de dos o tres cuestionarios^(34-35,43,50,55,59,67,73-74)^.

En la población estudiada, se observó una predominancia de pacientes con IC, representando el 68,18% del total^(37,43-46,51-53,59-60,62,65-67,63-64,66,68-70,72-74)^. La CVRS en estos pacientes fue evaluada utilizando 10 cuestionarios, incluidos seis específicos para esta población: MLHFQ^(38,45-48,50,59,62,67-69,73)^, KCCQ^(34,51,53,37)^, *MacNew* ^(31)^, *HeartQol* ^(54)^, HFSS^(65)^, 70-item *Quality of Life Index – Cardiac* ^(73)^, y cuatro cuestionarios genéricos: SF-36^(34,41,44,52,56,59-60,64,66,67,70,74)^, SF-8^(66)^, WHO-5^(34)^, y ED-5Q^(50,71-72)^. Considerando el contexto de la IC, el uso del instrumento MLHFQ se considera positivo, como se muestra en una revisión sistemática previa, que lo señaló como la escala más indicada para medir la CVRS en estos individuos, siendo el KCCQ una opción secundaria^(77)^.

Con respecto a las repercusiones en la CVRS de los pacientes, una parte significativa de los estudios incluidos (45,45%) evidenció resultados positivos, con significancia estadística, en el grupo de intervención^(33-34,41,45,47-49,51,54-55-58-60-65-68-70)^. Alrededor del 11,36% de los ECR no indicaron ningún impacto^(36,38,40,66-67)^, mientras que otro 43,18% mostró mejoras en la CVRS, aunque sin alcanzar diferencias estadísticamente significativas^(31-32,35,37,39,42-44,46,50,52-53,56,57,59,71,74)^. Esta diversidad de resultados resalta la complejidad y la naturaleza multifacética de la CVRS como un desenlace de salud, destacando la importancia de considerar factores contextuales y específicos de cada intervención al evaluar su impacto en la vida de los pacientes^(75-76)^. Además, aunque la telemedicina es prometedora, su eficacia varía debido a diferencias metodológicas, características de las poblaciones incluidas y tipos de intervención. Este hallazgo está en línea con un metaanálisis sobre la efectividad de la telemedicina en el manejo de las ECNT, que indicó una mejora en la CVRS en estudios sobre enfermedades cardiovasculares, aunque sin significancia estadística^(78)^.

En este sentido, otro aspecto importante a destacar es que los resultados de las intervenciones en telemedicina pueden equipararse a los cuidados presenciales tradicionales, sin demostrar inferioridad. Ninguno de los estudios incluidos identificó deterioro en la CVRS entre los participantes. Esto sugiere que la implementación de la telemedicina en el cuidado de condiciones cardiovasculares no conlleva consecuencias adversas en la CVRS de los pacientes y puede generar beneficios en otros desenlaces de salud, como la actividad física^(32,34,39-42,47,52,54,57,61)^, el uso de recursos de salud (hospitalizaciones, días de internación, visitas a emergencias)^(45,69,71,73)^, la salud mental^(38,46,53,63,73)^ y el autocuidado^(31,38,48,60,62)^.

Con respecto a las intervenciones utilizadas en los estudios, se observó que el telemonitoreo fue el recurso más utilizado. Cuando se empleó de forma aislada, mostró repercusiones positivas en la CVRS en solo el 36,36% (4 de 11) de los estudios^(48-49,63-64)^. De manera similar, los ECA que utilizaron únicamente llamadas telefónicas demostraron repercusiones positivas en la CVRS en solo el 33,33% (1 de 3)^(61)^. Sin embargo, las intervenciones combinadas, como la asociación del telemonitoreo con llamadas telefónicas, videoconsultas o SMS, mostraron diferencias estadísticamente significativas en 7 de 11 estudios (63,63%)^(34,45,51,60,62,68,70)^. En el caso de la telerrehabilitación, que siempre estuvo asociada a otro recurso mencionado, se observó una repercusión positiva en la CVRS en el 38,46% (5 de 13) de los ECA^(41,47,54-55,58)^. Estos resultados mixtos también se reflejaron en una revisión sistemática de 19 estudios sobre telemonitoreo en la insuficiencia cardíaca, que identificó heterogeneidad en los estudios que midieron la CVRS, así como una calidad metodológica dudosa y limitaciones en el tamaño de la muestra^(79)^.

Asimismo, con respecto a la telerrehabilitación, hubo mejoras en la CVRS en ocho estudios, pero sin superioridad en relación con el cuidado habitual^(32,35,39,42,50,52,56-57)^, lo cual es corroborado por una revisión sistemática que comparó la telerrehabilitación con la rehabilitación basada en centros, demostrando que esta intervención es igualmente efectiva en la mejora de la CVRS^(23)^.

En este sentido, considerando que la telerrehabilitación cardíaca parece aceptable para los pacientes y comparable al cuidado habitual, puede ser una forma eficaz de aumentar el alcance y la adherencia a la rehabilitación^(80)^. Sin embargo, se necesitan estudios futuros para explorar cómo la telerrehabilitación puede integrarse de forma eficaz en los sistemas de salud y cómo los profesionales de la salud pueden ser capacitados y apoyados para brindar este tipo de cuidado de manera adecuada.

Con respecto al tiempo de seguimiento de los estudios, se observó una variación significativa entre los ECA, con períodos que varían de uno a 26 meses, lo que puede contribuir a la heterogeneidad de los resultados. Considerando el impacto positivo y con significancia estadística en la CVRS, 10 estudios realizaron seguimientos de hasta seis meses^(47,49,51,54-55,58,61-62,65,68)^. Ocho ECA siguieron a los pacientes durante 12 meses^(33-34,48,60,63-64,69-70)^, mientras que un estudio realizó el seguimiento durante 24 meses^(45)^ y otro durante 26 meses^(41)^.

En una revisión sistemática previa, se observó que la mejora en la CVRS parece ser más evidente a corto plazo en pacientes con EAC y en pacientes con IC a lo largo de tres meses^(81)^. Esto sugiere la necesidad de nuevos estudios sobre la relación entre el tiempo de seguimiento, la población y los recursos de telemedicina para ayudar a identificar el mejor cuidado para cada paciente, considerando que la literatura aún es incipiente en este aspecto.

Así, el uso de metodologías más robustas y mejoradas, además de la estandarización de los protocolos de intervenciones en telemedicina para enfermedades cardíacas, debe ser incentivado, ya que esto contribuirá a una comprensión más sólida de los beneficios y desafíos reales de este enfoque en el contexto clínico de estas condiciones.

En cuanto a las limitaciones de los estudios seleccionados, se observó una predominancia de cuestiones relacionadas con el tamaño de la muestra, evidenciadas en el análisis crítico de los estudios. Solo un estudio informó el cegamiento de los participantes^(63)^, dos estudios mencionaron el cegamiento de los profesionales que realizaron las intervenciones^(33,47)^, y 12 estudios indicaron que los evaluadores de los resultados estaban cegados respecto a la asignación de los participantes^(32-33,36,40-41,47,50,52-55,64)^. Además, se mencionaron otras limitaciones, como el tamaño de la muestra^(31-32,37-40,43-45,58,60,62,67,69,74)^, el tiempo de seguimiento^(31,35,41,44,49,56)^ y el sesgo de selección o reclutamiento^(34-35,41,46-47,50-54,66-68,73)^, muchas veces debido a la predominancia de una enfermedad cardiovascular específica, a la presencia mayoritaria de la población masculina o incluso a la ubicación del reclutamiento.

Como implicaciones para la práctica, el uso de los recursos de la telemedicina puede considerarse una herramienta de apoyo esencial para proporcionar cuidado integral al individuo en entornos intra y extrahospitalarios, considerando principalmente sus beneficios en diversos desenlaces de salud. Sin embargo, aún se necesitan estudios robustos para medir mejor el efecto de la estrategia de telemedicina y su influencia en los desenlaces de salud, en cuanto al tiempo de seguimiento, la satisfacción de los pacientes y los profesionales, y la equidad en el acceso, teniendo en cuenta que las interacciones entre los desenlaces son complejas y deben considerar los diversos contextos y poblaciones.

En lo que respecta a la evaluación de la CVRS, es importante destacar la necesidad de estudios con enfoques multifacéticos, considerando la complejidad de la medición de esta variable, debido a su naturaleza multidimensional, con el fin de garantizar interpretaciones más precisas^(75-76)^. Además, también es relevante profundizar en las relaciones entre el tiempo de seguimiento, la población y la intervención de telemedicina.

Asimismo, políticas públicas eficaces e intervenciones adaptadas a las necesidades de cada comunidad^(9)^, basadas en protocolos estandarizados, son de extrema importancia, ya que tienen el potencial de ampliar el acceso a la salud, superando las barreras geográficas en la promoción del cuidado^(15)^.

Entre las limitaciones de esta revisión, destaca la no inclusión de la literatura gris, lo que podría haber ampliado la búsqueda de evidencia complementaria. Además, la revisión enfrentó desafíos relacionados con la heterogeneidad de las intervenciones y los recursos utilizados, así como la especificidad de las enfermedades cardíacas abordadas y los desenlaces evaluados. Estas variaciones dificultaron la realización de un metaanálisis, impidiendo una síntesis cuantitativa robusta. No obstante, los resultados cualitativos siguen siendo válidos para evaluar la efectividad de las intervenciones en los contextos estudiados.

## Conclusión

La efectividad de la telemedicina en la CVRS de individuos con EC sigue siendo inconclusa. Aunque la mayoría de los estudios ha demostrado un impacto positivo, muchos no alcanzaron significancia estadística. Las principales intervenciones utilizadas en el cuidado y autogestión de estas condiciones incluyen telemonitoreo, contacto telefónico y telerrehabilitación. La telemedicina tiene el potencial de ser una herramienta valiosa, comparable a las intervenciones presenciales en centros de salud. No obstante, se necesitan más estudios que evalúen su seguridad, costo-efectividad y otros desenlaces a largo plazo, especialmente el seguimiento de la CVRS, para optimizar la implementación de estas tecnologías y garantizar mejores resultados para los pacientes.
